# In Vitro and In Silico Studies of the Antimicrobial Activity of Prenylated Phenylpropanoids of Green Propolis and Their Derivatives against Oral Bacteria

**DOI:** 10.3390/antibiotics13080787

**Published:** 2024-08-22

**Authors:** Tatiana M. Vieira, Julia G. Barco, Sara L. de Souza, Anna L. O. Santos, Ismail Daoud, Seyfeddine Rahali, Noureddine Amdouni, Jairo K. Bastos, Carlos H. G. Martins, Ridha Ben Said, Antônio E. M. Crotti

**Affiliations:** 1 Department of Chemistry, Faculty of Philosophy, Science and Letters at Ribeirão Preto, University of São Paulo, Ribeirão Preto 14040-901, SP, Brazil; tati.manzini@gmail.com (T.M.V.); juliagrassibarco@usp.br (J.G.B.); 2 Department of Microbiology, Institute of Biomedical Sciences, Federal University of Uberlândia, Uberlândia 38405320, MG, Brazil; saralemessouza@ufmg.br (S.L.d.S.); anna.livia@ufu.br (A.L.O.S.); carlos.martins2@ufu.br (C.H.G.M.); 3 Department of Matter Sciences, University Mohamed Khider, BP 145 RP, Biskra 07000, Algeria; i.daoud@univ-biskra.dz; 4 Laboratory of Natural and Bio-Active Substances, Faculty of Science, Tlemcen University, Tlemcen P.O. Box 119, Algeria; 5 Department of Chemistry, College of Science, Qassim University, Qassim 51452, Saudi Arabia; s.rahali@qu.edu.sa; 6 Laboratoire de Caractérisations, Applications et Modélisations des Matériaux, Faculté des Sciences de Tunis, Université Tunis El Manar, Tunis 1068, Tunisia; noureddine.amdouni@fst.utm.tn; 7 School of Pharmaceutical Sciences of Ribeirão Preto, University of São Paulo, Ribeirão Preto 14040-903, SP, Brazil; jkbastos@fcfrp.usp.br

**Keywords:** artepillin C, molecular docking, oral bacteria, oral pathogens, plicatin B

## Abstract

Artepillin C, drupanin, and plicatin B are prenylated phenylpropanoids that naturally occur in Brazilian green propolis. In this study, these compounds and eleven of their derivatives were synthesized and evaluated for their in vitro antimicrobial activity against a representative panel of oral bacteria in terms of their minimum inhibitory concentration (MIC) and minimum bactericidal concentration (MBC) values. Plicatin B (**2**) and its hydrogenated derivative **8** (2′,3′,7,8-tetrahydro-plicatin B) were the most active compounds. Plicatin B (**2**) displayed strong activity against all the bacteria tested, with an MIC of 31.2 μg/mL against *Streptococcus mutans, S. sanguinis*, and *S. mitis.* On the other hand, compound **8** displayed strong activity against *S. mutans*, *S. salivarius*, *S. sobrinus*, *Lactobacillus paracasei* (MIC = 62.5 μg/mL), and *S. mitis* (MIC = 31.2 μg/mL), as well as moderate activity against *Enterococcus faecalis* and *S. sanguinis* (MIC = 125 μg/mL). Compounds **2** and **8** displayed bactericidal effects (MBC: MIC ≤ 4) against all the tested bacteria. In silico studies showed that the complexes formed by compounds **2** and **8** with the *S. mitis*, *S. sanguinis*, and *S. mutans* targets (**3LE0**, **4N82**, and **3AIC**, respectively) had energy score values similar to those of the native *S. mitis*, *S. sanguinis*, and *S. mutans* ligands due to the formation of strong hydrogen bonds. Moreover, all the estimated physicochemical parameters satisfied the drug-likeness criteria without violating the Lipinski, Veber, and Egan rules, so these compounds are not expected to cause problems with oral bioavailability and pharmacokinetics. Compounds **2** and **8** also had suitable ADMET parameters, as the online server pkCSM calculates. These results make compounds **2** and **8** good candidates as antibacterial agents against oral bacteria.

## 1. Introduction

Oral pathogens comprise a group of microorganisms that adhere to and colonize the oral cavity, causing periodontal inflammatory diseases, caries, or infective endocarditis [1]. Oral bacteria are responsible for dental caries, an infectious disease that affects people worldwide, especially in low-income regions of developing countries [2]. These bacteria adhere to the mature acquired salivary pellicle [3,4], which provides specific receptors for bacteria to attach and initiate colonization of the tooth surface [5]. The initial colonizers that adhere to acquired salivary pellicles are usually *Streptococcus* species and *Actinomyces* species [6,7]. *Streptococcus mutans*, one of the primary bacteria associated with dental caries, can establish a strongly acidic microenvironment (pH below 5.0) due to acidic by-products of the bacterial fermentation of different types of carbohydrates from the diet [8], thereby forming biofilms on the tooth surface (i.e., the dental plaque), which culminates in teeth cavitation (i.e., caries) and the onset of tooth decay [9]. However, oral diseases generally have a polymicrobial origin, and together with *S. mutans*, other acid-resistant bacteria can cause cavities, including *S. sobrinus* and *S. sanguinis* [10].

The most effective method of preventing dental biofilm is through mechanical removal by brushing and flossing [11]. However, in most cases, this mechanical method is performed improperly, especially in hard-to-reach places, such as sub-gums and fissures, which end up favoring biofilm accumulation. Thus, the use of chemical methods, such as mouthwashes with antimicrobial action, becomes necessary to promote the reduction of the adhesion of bacteria to the dental surface and inhibit cariogenic bacteria growth and proliferation [12,13]. Chlorhexidine is the gold standard anti-cariogenic agent used in mouthwash formulations to reduce plaque [13,14]. However, despite its effectiveness, chlorhexidine has shown adverse effects, especially in terms of its daily use, in addition to the emergence of resistant strains [15,16]. In this scenario, the interest in new compounds with antimicrobial action as effective as chlorhexidine in controlling biofilm but with fewer adverse effects has grown in the last few years [17,18].

Propolis is a resinous product formed by plant material collected and deposited in hives by bees. The chemical composition of propolis depends on the botanical sources of each region [19]. Brazilian green propolis (BGP) is produced by *Apis mellifera* using shoot apices of *Baccharis dracucunlifolia* (Asteraceae) in the country’s southeast region [20]. Because of its wide diversity of biological and pharmacological activities [21,22,23,24,25,26,27,28], BGP has also been used in folk medicine and exported to several countries (e.g., Japan, China, Russia, France, and Germany) as sprays, toothpaste, soaps, ointments, and creams for skin [29]. The antimicrobial properties of BGP are of particular interest and include activity against several Gram-positive (*Staphylococcus aureus* [30,31,32,33], *Staphylococcus saprophyticus* [30], *Listeria monocytogenes* [30], *Enterococcus faecalis* [30]) and Gram-negative bacteria (*Enterobacter aerogenes* [30], *Salmonella typhimurium* [30], *Shigella flexneri* [30], *Escherichia coli* [30,33], *Pseudomonas aeruginosa* [30,33,34], *Providencia rettgeri* [30], and *Klebsiella pneumoniae* [30]) and *Candida albicans* [33]. BGP also has bacteriostatic effects on *Streptococcus mutans* cultures [35] and is a potent antibacterial agent against *S. mutans* [36].

**Figure 1:**
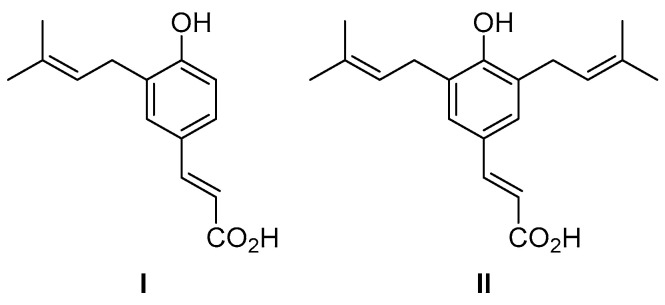
Chemical structures of drupanin (**I**) and artepillin C (**II**).

Chemically, Brazilian green propolis is rich in prenylated compounds [37,38], mainly phenylpropanoids like drupanin (**I**), and artepillin C (**II,** Figure 1). These phenolic acids are commonly detected and identified in Brazilian green propolis [32,39,40,41]. In the literature, many studies have reported the biological and pharmacological activities of artepillin C [32,39,42,43] and drupanin [40,41,44]. However, data on the antibacterial activity of these prenylated compounds against oral bacteria are still scarce [45].

In this study, we investigated the antibacterial activity of a series of synthetic prenylated phenylpropanoids, including the naturally occurring compounds artepillin C and drupanin and their derivatives, against a representative panel of oral bacteria. In silico studies with the most active compounds aiming to understand the target–compound interactions and to estimate the physicochemical, drug-likeness, and ADMET/pharmacokinetic properties were also performed.

## 2. Results

### 2.1. In Vitro Antibacterial Activity of Compounds ***1***–***14***

**Scheme 1:**
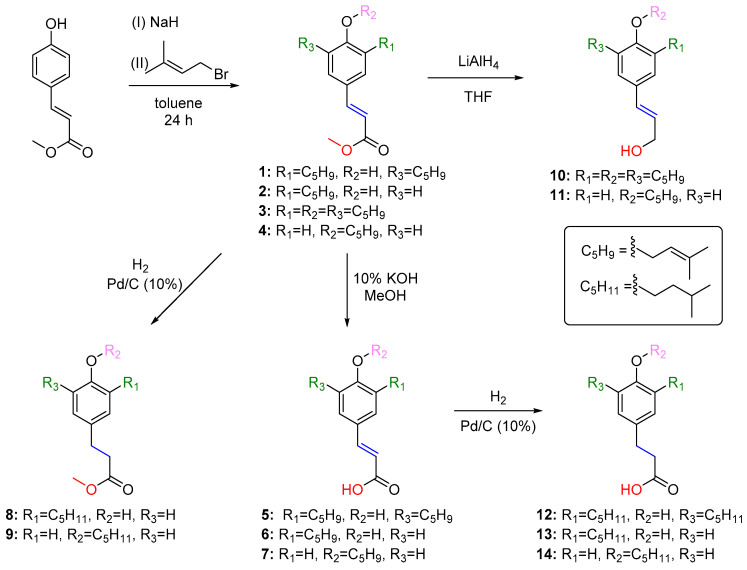
Synthesis of compounds **1**–**14**.

Artepillin C (**5**), drupanin (**6**), and compounds **7**–**14** were obtained by hydrolysis, reduction with LiAlH_4_, and/or catalytic hydrogenation of prenylation products of methyl *p*-coumarate **1**–**4**, as shown in Scheme 1. Table 1 summarizes the results of the antimicrobial assays with the fourteen compounds against a representative panel of oral bacteria regarding their minimum inhibitory concentration (MIC) and minimum bactericidal concentration (MBC). The lowest MIC values (31.2 µg/mL) were observed for compound **2** (plicatin B) against *Streptococcus mutans* (*S. mutans*), *Streptococcus mitis* (*S. mitis*), and *Streptococcus sanguinis* (*S. sanguinis*) and for compound **8** against *S. mitis*.

### 2.2. In Silico Studies on the Antibacterial Activity of ***2*** and ***8***

#### 2.2.1. Target–Compound Interactions

Some information related to the crystal structures of the *S. mitis* (PDB ID: **3LE0**) [46], *S. sanguinis* (PDB ID: **4N82**) [47], and *S. mutans* (PDB ID: **3AIC**) [48] targets are given in Table 2. The docking simulation results for compounds **2** and **8**, the most active compounds against the selected oral bacteria, along with both X-ray crystals of the studied targets, are listed in Table 3, Tables S1 and S2.

**Figure 2:**
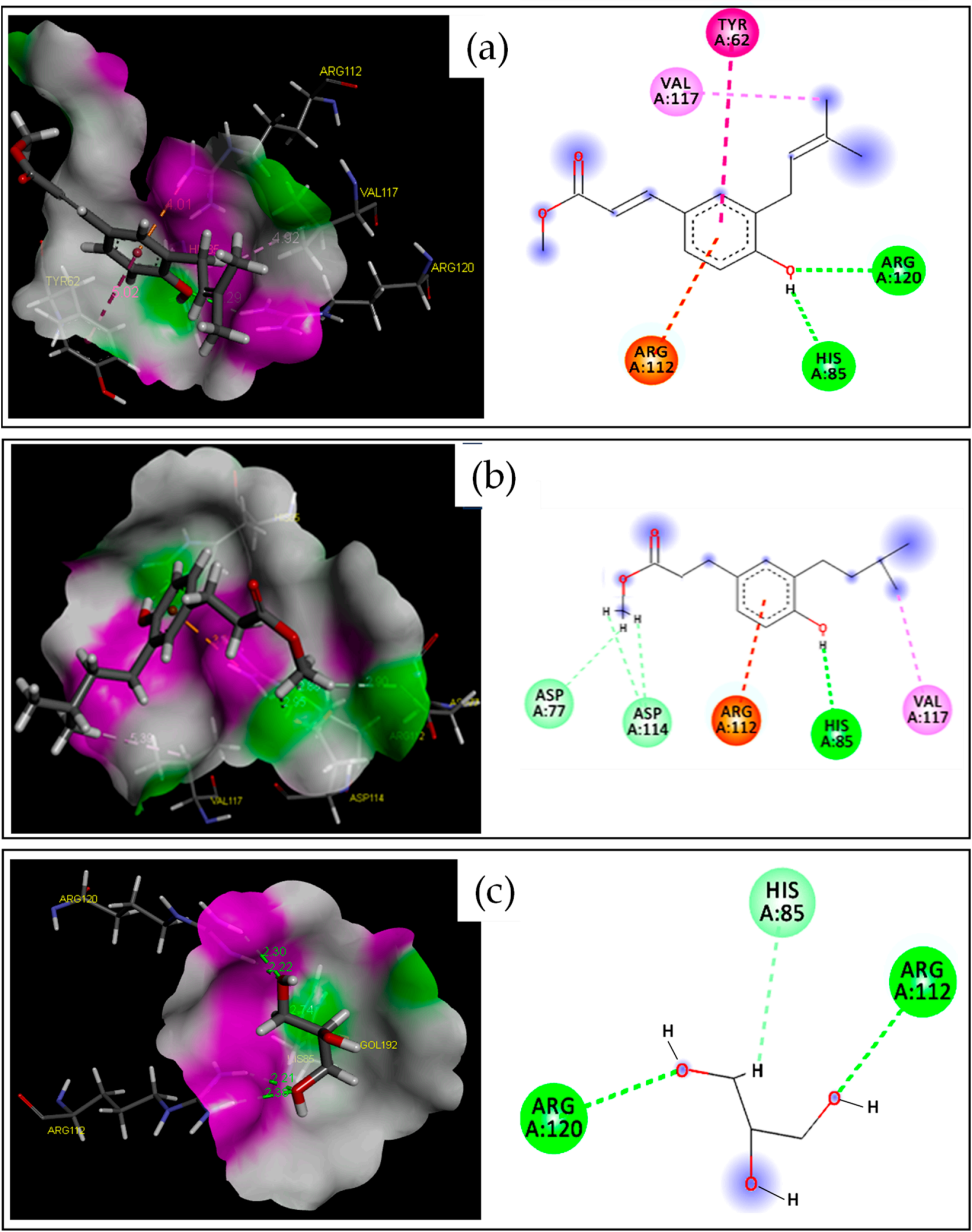
Three- and two-dimensional diagrams of the interaction between the *S. mitis* target (**3LE0**) and compound **2** (**a**), compound **8** (**b**), and the native ligand (GOL) (**c**).

The complexes formed by compounds **2** and **8** with the active site residue of *S. mitis* (**3LE0**) have low energy values (−4.228 and −4.476 kcal/mol, respectively, Table 3) compared to the native ligand glycerol (GOL, −3.655 kcal/mol). Four strong hydrogen bonds between compound **8** and the active site residue of the *S. mitis* (**3LE0**) target were observed: one conventional H-bond type (H/HIS85(A)-NE2/bond distance = 2.23 Å) and three other carbon H-bonds (H/ASP114(A)-OD1/bond distance = 2.87 Å, H/ASP77(A)-OD2/bond distance = 2.90 Å), and H/ASP114(A)-OD1/bond distance = 2.95 Å). One electrostatic interaction between compound **8** and ARG112(A) was also formed. On the other hand, this compound formed one hydrophobic interaction with VAL117(A) (Table 3 and Figure 2b). In addition, the native ligand (GOL) formed three strong hydrogen bonds, two conventional hydrogen bonds with CYS241 and one carbon hydrogen bond with HIS85(A) (Figure 2c). Noteworthily, the residues ARG120(A), ARG112(A), and HIS85(A) were found in the binding site of the *S. mitis* target and they were involved in different interactions in the compounds **2**, **8**, and native ligand (GOL) (Figure 2a–c).

**Figure 3:**
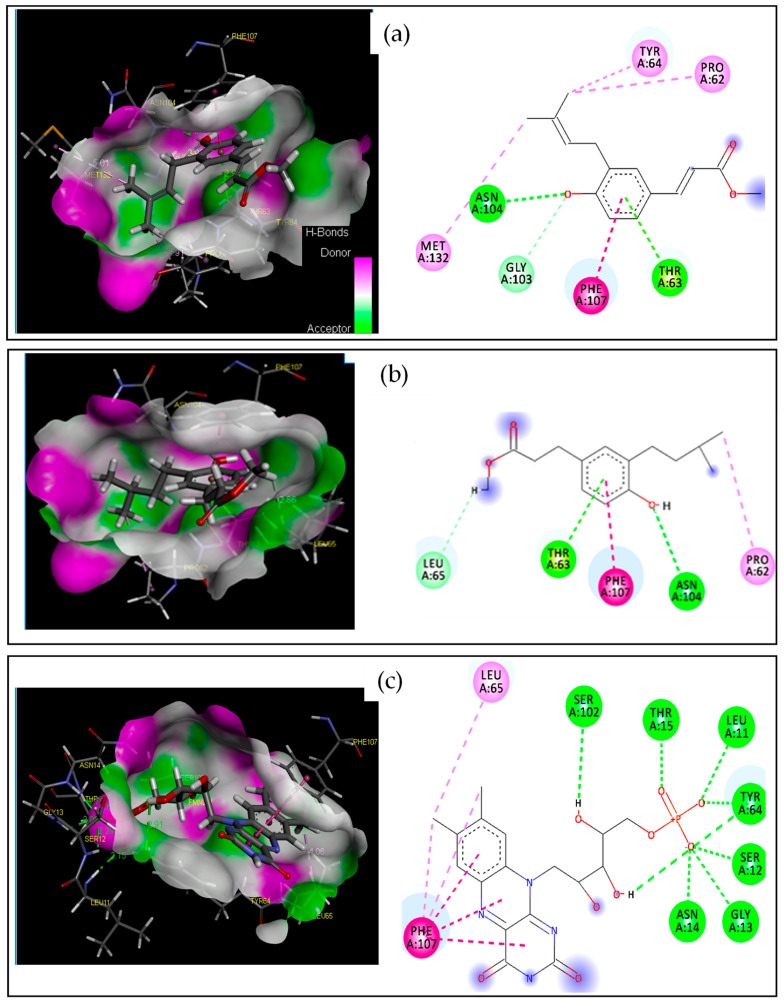
Three- and two-dimensional diagrams of the interactions between the *S. sanguinis* target (**4N82**) and compound **2** (**a**), compound **8** (**b**), and the native ligand (FMN) (**c**).

In the case of the *S. sanguinis* (**4N82**) target, the complex formed by compound **2** gave a high negative energy value (−6.156 kcal/mol) as compared to compound **8** (Table S1). In addition, the score value of compound **2** (−6.156 kcal/mol) was very close to the native ligand FMN (−6.671 kcal/mol) (Table S1). The docked conformation of compound **2** with the *S. sanguinis* (**4N82**) target is shown in Figure 3a. It can be noted that this compound makes two strong carbon H-bonds (O/ASN104(A)-H/bond distance = 2.37 Å, and O/GLY103(A)-HA3/bond distance = 2.93 Å), besides one Pi–lone pair interaction (with TYR63(A)) and four hydrophobic interactions with residues PHE107(A), MET132(A), PRO62(A), and TYR64(A) (Table S1 and Figure 3a). Moreover, the native ligand (flavin mononucleotide, or FMN) forms ten strong hydrogen bonds with the following residues: LEU11(A), SER12(A), GLY13(A), ASN14(A), THR15(A), TYR64(A), and SER102(A) (Figure 3c). In addition, the native ligand (FMN) established six hydrophobic interactions with two residues: PHE107(A), and LEU65(A) (Table S1 and Figure 3c). Lastly, it is noteworthy that (Figure 3a–c) the residues PHE107(A), and LEU65(A) appeared in the pocket site of the *S. sanguinis* target and were involved in different interactions in compounds **2**, **8**, and the native ligand (FMN) (Figure 2a–c).

**Figure 4:**
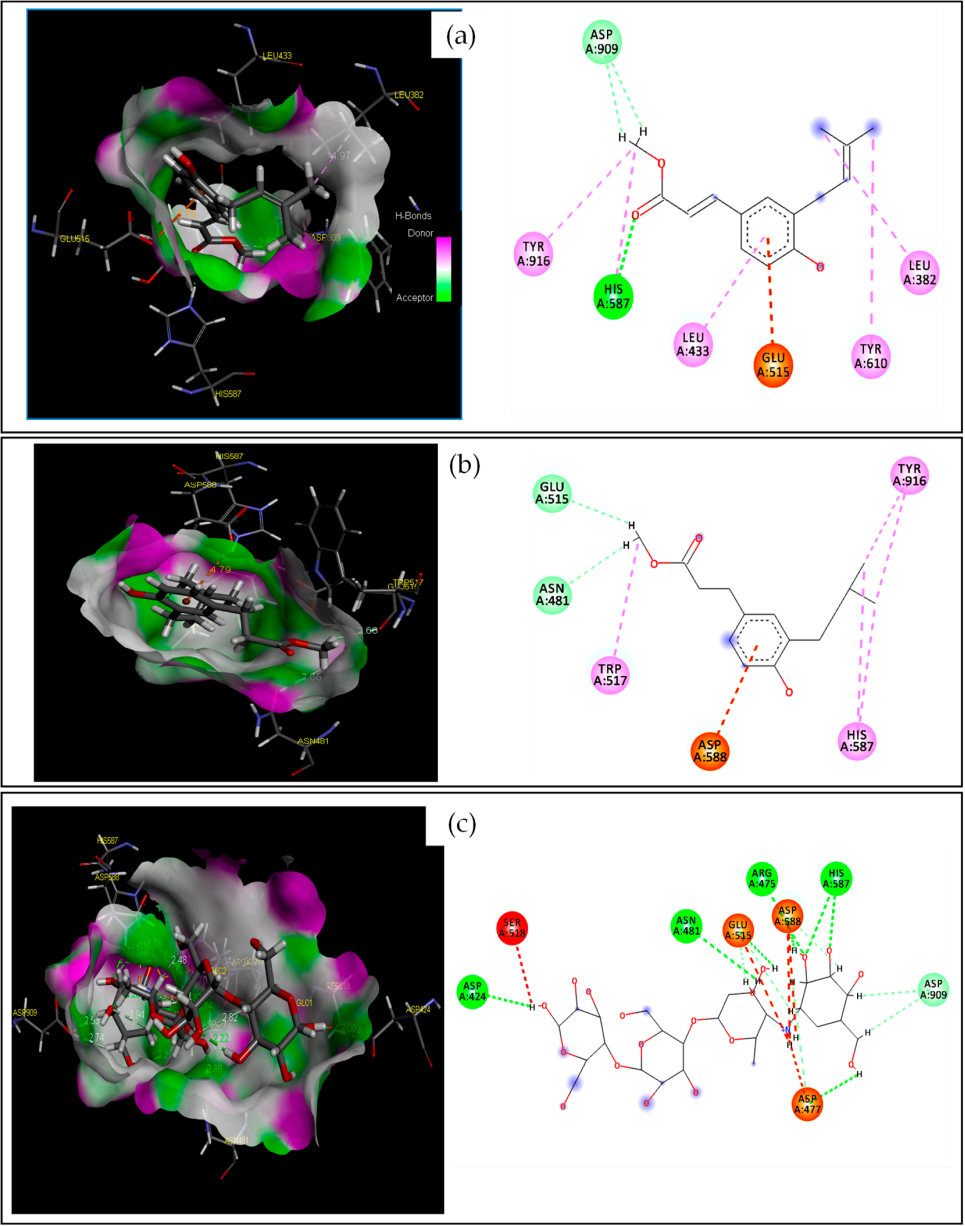
Three- and two-dimensional diagrams of the interactions between the *S. mutans* target (**4N82**) and compound **2** (**a**), compound **8** (**b**), and the native ligand (FMN) (**c**).

The complexes formed by compounds **2** and **8** with the *S. mutans* (**3AIC**) target had low energy values of −5.049 and −5.042 kcal/mol, respectively, which were similar to those of the native ligand, acarbose (ACA, −6.674 kcal/mol, Table S2). Furthermore, three strong hydrogen bonds between compound **2** and the active site residue of the *S. mutans* (**3AIC**) target were observed: one conventional H-bond type (O/HIS587(A)-HE2/bond distance = 2.04 Å) and two other carbon H-bonds (H/ASP909(A)-OD1/bond distance = 2.85 Å, and H/ASP909(A)-OD1/bond distance = 2.74 Å). An electrostatic interaction with GLU515(A) was also observed. In contrast, this compound formed five hydrophobic interactions with the following active site residues: LEU382(A), HIS587(A), TYR610(A), TYR916(A), and LEU433(A) (Table S2 and Figure 4a). This result was supported by several recent studies [49,50,51].

#### 2.2.2. Molecular Dynamics Simulation

**Figure 5:**
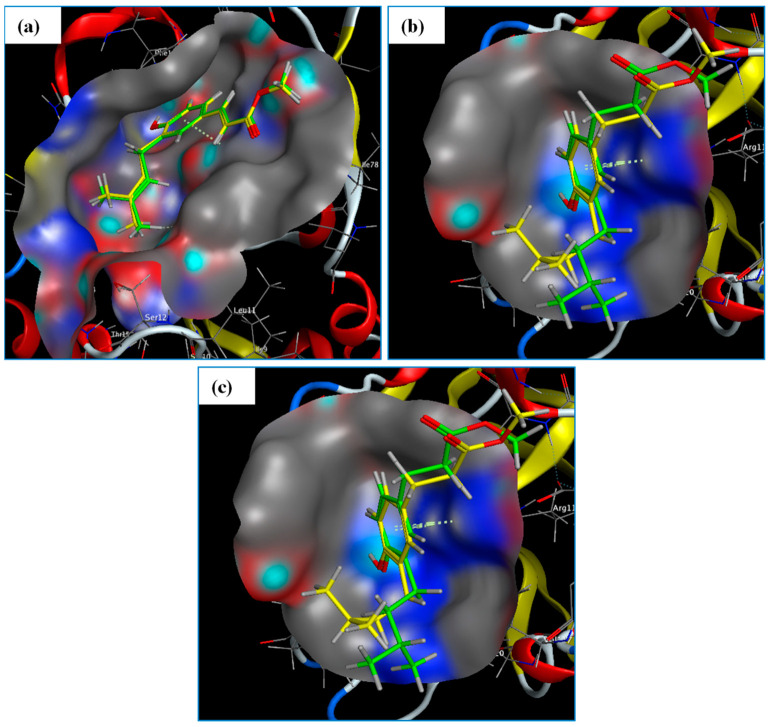
Three-dimensional diagrams of the best pose found by molecular docking (green) and molecular dynamics (yellow) for **3LE0**–compound **8** (**a**), **4N82**–compound **2** (**b**), and **3AIC**–compound **2** (**c**).

**Figure 6:**
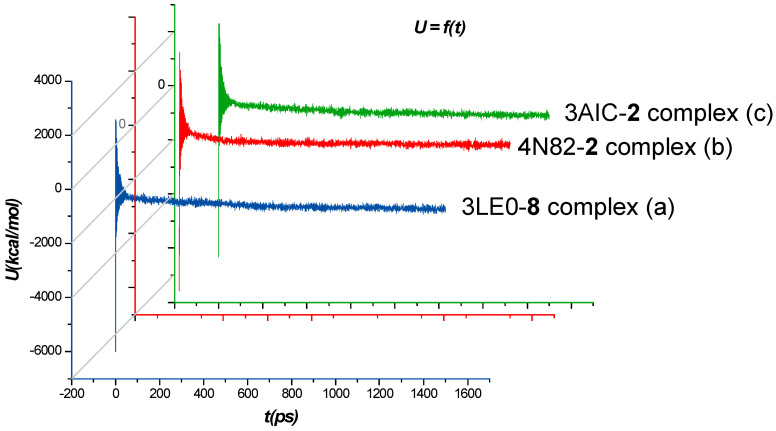
Potential energy (U) as a function of time for three complexes; (a) **3LE0**–compound **8**, (b) **4N82**–compound **2**, and (c) **3AIC**–compound **2**.

Figure 5 presents the 3D diagrams of the best pose for compounds **2** and **8** with the active binding sites **3LE0**, **4N82**, and **3AIC**, as determined by molecular docking (green) and molecular dynamics (yellow) studies. As shown in Figure 6, a fluctuation in all the curves was initially observed during the first 100 picoseconds. A slight variation in the potential energy was observed between 100 and 800 ps in curve (a), and between 100 and 400 ps in curve (e), and between 100 and 900 ps in curve (c). Finally, the **3LE0**–compound **8**, **4N82**–compound **2**, and **3AIC**–compound **2** complexes retained their stabilities in the last intervals (between 800 and 1500 ps, between 400 and 1500 ps, and between 900 and 1500 ps, respectively) (Figure 6a–c).

#### 2.2.3. Drug-Likeness Prediction and ADMET Properties

##### Drug-Likeness Evaluation

Different physicochemical properties were calculated for compounds **2** and **8**, aiming to verify the drug-likeness rules using the SwissADME online server. As shown in Table 4, it is apparent that compounds **2** and **8** have hydrogen bond donors < 5 (n-HD: 0~7) and hydrogen bond acceptors < 10 (n-HA: 0~10). In addition, the molecular weight values of these compounds belong to the interval 100~500 g/mol, and their MLogP and WLogP values are <5. Also, the nROTB values are <11, which denotes the flexibility of these compounds. Moreover, the TPSA values obtained for both compounds are less than 140 Å. 

##### ADMET Properties

The absorption, distribution, metabolism, excretion, and toxicity (ADMET) were calculated for compounds **2** and **8** using the online server pkCSM (Table 5). The Caco-2 values obtained for **2** and **8** are 1.178 and 1.417, respectively, whereas the human intestinal absorption (HIA) values are 99.207% and 97.682%, respectively. In terms of the distribution, the logPS values of **2** and **8** are 1.800 and −1.692, respectively, while their logBB values are 0.311 and 0.030, respectively (Table 5). Compounds **2** and **8** are CYP1A2 inhibitors, but not CYP2C19 and CYP2D6 inhibitors. In addition, these compounds are not CYP2D6 and CYP3A4 substrates and are likely not OCT2 substrates. These compounds have a total clearance of 0.744 and 0.780, respectively. Furthermore, compounds **2** and **8** are neither hERG I nor hERG II inhibitors.

## 3. Discussion

### 3.1. In Vitro Antibacterial Activity of Compounds ***1***–***14***

The antimicrobial activity of compounds **1**–**14** was classified based on the MIC (minimum inhibitory concentration) values as follows: MIC values lower than 10 μg/mL, between 11 and 100 μg/mL, between 101 and 500 μg/mL, and between 501 and 1000 μg/mL correspond to very strong, strong, moderate, and weak activities, respectively, while MIC values higher than 1000 μg/mL denote inactivity [1,52,53,54]. Based on these criteria, compounds **2** and **8** displayed strong or moderate activity against all the tested bacteria (Table 1). However, the lowest MIC value was observed for **2** (plicatin B), which showed strong activity against *S. mutans*, *S. mitis*, and *S. sanguinis* (MIC = 31.2 µg/mL). This compound also showed strong activity against *S. salivarius* and *S. sobrinus* (MIC = 62.5 µg/mL). The strong activity of compound **2** against *S. mutans* is an auspicious result because it is one of the leading agents causing dental caries [1]. Plicatin B (**2**) has been isolated from Brazilian propolis samples, even though its occurrence is less commonly reported [55]. However, data on the antimicrobial activity of plicatin B are still scarce in the literature [56]. On the other hand, artepillin C (**5**) and drupanin (**6**), which are among the most common prenylated compounds found in Brazilian green propolis, showed moderate or weak activity against most of the tested bacteria (MIC between 125 and 1000 µg/mL), with drupanin displaying MIC values slightly lower than artepillin C. Compounds **1**, **10**, and **14** had no activity against any of the oral bacteria tested, with MIC values higher than 1000 µg/mL. 

According to the literature, compounds with an MBC:MIC ratio ≤ 4, and 4 < MBC:MIC ≤ 32 display bactericidal and bacteriostatic effects, respectively, whereas MBC:MIC values > 32 denote a bacteria-resistant effect [57]. Based on these criteria, compounds **2** and **8** are considered to display bactericidal activity against all the oral bacteria tested in this study. 

We compared our data on the antibacterial activity of compounds **1**–**14** with the relevant literature to derive insights into the potential structure–activity relationships. Based on the comparison of the antimicrobial activities of artepillin C (**5**), drupanin (**6**), and 4-hydroxycinnamic acid, Aga and coworkers considered that the antimicrobial activity of 4-hydroxycinnamic acid derivatives may be boosted by an increasing number of prenyl groups in the structure [58]. However, in this study, the mono-prenylated compound **2** (plicatin B) and its corresponding carboxylic acid **6** (drupanin) were more active against the tested oral bacteria than the di-prenylated compounds **1** and **5** (artepillin C), respectively. These results indicated clearly that other structural features than the prenyl groups also play a key role in the antibacterial activity of compounds **1**–**14**. For example, the phenolic hydroxyl is a crucial structural feature in relation to the antibacterial activity, as evidenced by the comparison between the MIC values of the isomers **2** (a *C*-prenylated compound) and **4** (an *O*-prenylated compound). The role played by phenolic hydroxyl is likely due to its capability to form a hydrogen bond to the active binding sites of the bacteria enzymes, thus inhibiting microbial enzymes and simultaneously increasing the affinity with cytoplasmic membranes [59]. This is reinforced by the higher activity of **2** (a mono-*C*-prenylated ester) compared to compound **3** (a tri-prenylated ester that contains a prenyl group at the oxygen at C4), despite the higher lipophilicity of **3**. Indeed, docking studies confirmed the formation of a hydrogen bond between the phenolic hydroxyl of compounds **2** and **8** with the residues HIS 85(A), and ASN 104(A) in *S. mitis*, and *S sanguinis*, respectively.

The nature of the oxygenated function at C9 and the type of covalent bond between C7 and C8 were also found to affect the antibacterial activity of compounds **1**–**14**; however, the effects of these structural features vary according to the number and position of the prenyl groups. For example, the mono-*C*-prenylated methyl ester **2** (plicatin B) and its hydrogenated derivative **8** are more active than their corresponding carboxylic acids **7** (drupanin) and **13**, indicating that the presence of an ester function at C9 may potentialize the antibacterial activity of mono-*C*-prenylated compounds. However, in the case of compounds di-*C*-prenylated **5** (artepillin C) and *O*-prenylated **7**, the carboxyl group at C9 increases the antibacterial activity as compared to the corresponding methyl esters **1** and **4**. Similarly, the presence of a single bond between C7 and C8 in the structure of mono-*C*-prenylated compounds **8** (a methyl ester) and **13** (a carboxylic acid) decreases the antibacterial activity as compared to compounds **2** and **6**, which display a double bond between C7 and C8. On the other hand, for compounds di- and tri-prenylated **12** and **9**, a single bond between C7 and C8 increases the antibacterial activity as compared to **5** and **3**, respectively. 

The important role played by the number and position of the prenyl groups in terms of the antibacterial activity of compounds **1**–**14** can be better understood based on the studies reported by Kubo and coworkers. According to those authors, the mechanism by which phenolic acids enter the molecular structure of the bacteria membrane involves the orientation of the phenol hydroxyl into the aqueous phase by hydrogen bonding, and the non-polar carbon chain alignment into the lipid phase by dispersion forces. The activity tends to disappear when the hydrophilic force exceeds the hydrophobic one [60]. Therefore, based on the literature [60], the antibacterial activity of compounds **1**–**14** can be understood in terms of the balance between hydrophilicity/lipophilicity. In this sense, compounds **2** and **8** can be inferred to achieve the best lipophilicity/hydrophilicity balance among the tested compounds. Hydrolysis of plicatin B (**2**) produces drupanin (**6**), which has a carboxyl group at C9. Although this group can anchor the compound to the lipid bilayer [61], it also increases the hydrophilicity of drupanin (**6**) as compared to plicatin B (**2**) and consequently decreases its antibacterial activity. On the other hand, the presence of two *C*-prenyl groups in the structure of artepillin C (**5**) increases lipophilicity as compared to **2** and **6**, thus decreasing its antibacterial activity. Finally, the presence of an α,β-unsaturated carbonyl conjugated with the aromatic ring of phenylpropanoids **1**–**7** confers conformational and electronic characteristics that are strongly influenced by the phenol-OH group in the *para* position [62]. In principle, because of the rotation around the single bond between C7 and C8, compounds **8**, **9**, **12**, **13**, and **14** can assume a wider variety of conformations as compared to **1**–**7**, **10**, and **11**, which could improve their intermolecular interactions with a potential target [63]. However, data from this study suggest a combined effect of the nature of the bond between C7 and C8 and the number and position of the prenyl groups on the molecular shape of compounds **1**–**14** and, consequently, on their antibacterial activity. 

### 3.2. Molecular Docking and Molecular Dynamics

The antibacterial activity of compounds **2** and **8** against *S. mitis*, *S. sanguinis*, and *S. mutans* was further investigated using molecular docking. Molecular docking is a computational technique that predicts the precise positioning of a ligand molecule over a receptor protein molecule to create a stable complex [64]. This orientation is essential for predicting the binding affinity and strength of the ligand–protein connection through the use of a scoring function [65]. The interaction between a drug and its receptor reduces the overall free energy of the system, providing insights into the molecule’s affinity and activity, which is crucial for drug design and discovery [66]. Docking studies can both describe the protein and the ligand as complementary surfaces [67,68] and provide the ligand–protein pairwise interaction energies [69]. 

The protein targets in *S. mitis* (**3LE0**) [46], *S. sanguinis* (**4N82**) [47], and *S. mutans* (**3AIC**) [48] were selected on the basis of their relevance to the microorganism virulence and the availability of their resolved crystallographic structures in the Protein Data Bank (PDB) [70]. Based on the binding score energy values, compounds **2** (−4.228 kcal/mol) and **8** (−4.476 kcal/mol) were found to inhibit *S. mitis* (**3LE0**) more effectively than the native ligand glycerol (GOL) (−3.655 kcal/mol). However, compound **8** was predicted to be a stronger *S. mitis* (**3LE0**) target binder compared to compound **2** due to the formation of a more stable complex, as indicated by its negative score energy of −4.476 kcal/mol. These results are in full agreement with the lower MIC value of compound **8** (0.12 mM) compared to compound **2** (0.13 mM) (Table 2). Furthermore, these results suggest that compound **8** has a high affinity with the *S. mitis* (**3LE0**) pocket, which is confirmed by establishing four strong hydrogen bonds (a conventional H-bond type and three carbon H-bonds) [71,72]. Of particular interest is the electrostatic interaction with ARG112(A). Recent studies have revealed that residues ARG112(A) and HIS85(A) play an essential role in the inhibition of the *S. mitis* (**3LE0**) target [73,74]. 

Conversely, compound **2** is more active against *S. sanguinis* (MIC = 31.2 µg/mL, 0.13 mM) than compound **8** (MIC = 62.5 µg/mL, 0.25 mM) (Table 1). Indeed, molecular docking with these compounds revealed the higher stability of the complex formed by compound **2** (score energy of −6.156 kcal/mol) with the protein target in *S. sanguinis* (**4N82**) compared to that of the complex formed by compound **4** (score energy of −5.575 kcal/mol). This stability is similar to that of the flavin mononucleotide (FMN), the native ligand (score energy of −6.671 kcal/mol, Table 3). This higher stability can be due to the two strong carbon H-bonds (O/ASN104(A)-H and O/GLY103(A)-HA3), one Pi–lone pair interaction (with TYR63(A)), and four hydrophobic interactions between compound **2** and residues PHE107(A), MET132(A), PRO62(A), and TYR64(A) of the *S. sanguinis* protein target (Table 3 and Figure 3A). Indeed, the role played by these residues in the stability of the formed complex has been reported [47,73].

The score energies of the complexes formed between the *S. mutans* target (**3AIC**) and compounds **2** (−5.049 kcal/mol) and **8** (−5.042 kcal/mol) are similar to those of acarbose (−6.674 kcal/mol, Table 3), the native ligand. Furthermore, compound **2** fits nicely into the *S. mutans* (**3AIC**) pocket due to the formation of three strong hydrogen bonds [71,72] with active site residue of the *S. mutans* (**3AIC**) target: one conventional H-bond type and two other carbon H-bonds. An electrostatic interaction with GLU515(A) was also observed. In contrast, this compound formed five hydrophobic interactions with the following active site residues: LEU382(A), HIS587(A), TYR610(A), TYR916(A), and LEU433(A) (Table 3, Figure 4A). This result was supported by several recent studies [49,50,51].

MD simulations were also performed to investigate the stability of the best complexes obtained through molecular-docking calculations for compounds **2** and **8** with the protein targets. Based on a generic model of the physics driving interatomic interactions, MD simulations predict the movements of individual atoms inside proteins and other molecular systems across time [75]. These simulations display the locations of all the atoms with femtosecond temporal precision, and they can capture a wide range of significant biomolecular processes, such as conformational change, ligand binding, and protein folding [76]. Crucially, these simulations can also forecast, down to the atomic level, how biomolecules will react to various perturbations like mutation, phosphorylation, protonation, or ligand addition or removal [77]. In this study, the stability of the best complexes obtained through molecular-docking calculations for compound **2** (**4N82**–compound **2** and **3AIC**–compound **2**) and **8** (**3LE0**–compound **8**) with the protein targets was investigated by MD simulations (Figure 5). The energy potential fluctuation observed in all the curves in the first 100 picoseconds (ps) (Figure 5) can be justified based on the variation in the potential energy of three complexes: **3LE0**–compound **8** (Figure 5a); **4N82**–compound **2** (Figure 5b); and **3AIC**–compound **2** (Figure 5c). However, all the studied complexes exhibit high stability, as confirmed by the MD simulations, because they maintained almost the same types of interactions compared to the molecular-docking studies. This was also confirmed by the stability of the potential energy as a function of time (Figure 5a–c), as reported in previous papers [78,79].

### 3.3. Drug-Likeness and ADMET Properties

We also estimated the drug-likeness and ADMET properties of compounds **2** and **8**. The drug-likeness considers how simple physicochemical properties impact molecular behavior in vivo [80]. Lipinski’s “rule-of-five” predicts the drug-likeness of an oral therapeutic agent based on its physicochemical properties [81]. This depicts the connection between physicochemical and pharmacokinetics indices [82]. According to this rule, an orally active drug-like compound should not have more than one violation of the following criteria: hydrogen bond donors not greater than 5, hydrogen bond acceptors not greater than 10, molecular weight not greater than 500 Da, and an octanol–water partition coefficient (log P) not greater than 5 [81,82]. As shown in Table 4, all the physicochemical properties of compounds **2** and **8** follow Lipinsky’s rule. 

The ADMET (absorption, distribution, metabolism, excretion, and toxicity) properties play a critical role in sorting ligands in drug discovery programs, as they account for the failure of 60% of drug candidates during the drug development process [83]. In this study, the Caco-2 values obtained for **2** and **8** are higher than −5.15 (>−5.15 cm/s), which confirms that these compounds have good human intestinal permeability. Moreover, both compounds have HIA values higher than 30%, which means that compounds **2** and **8**, when administered orally, can be absorbed from the gastrointestinal system into the human body’s bloodstream. The logPS values (−3< logPS <−2) indicate that compounds **2** and **8** cannot penetrate the CNS. Additionally, the logBB values of compounds **2** (0.311) and **8** (0.030) indicate that compound **2** is expected to readily cross the blood–brain barrier, whereas compound **8** is poorly distributed in the brain. Compounds **2** and **8** are CYP1A2 inhibitors, but not CYP2C19 and CYP2D6 inhibitors. In addition, these compounds are not CYP2D6 and CYP3A4 substrates and are not likely OCT2 substrates. These compounds are also expected to have a low clearance (<5 mL/min/kg). Furthermore, compounds **2** and **8** are neither hERG I nor hERG II inhibitors, showing no hepatotoxicity risk. Therefore, compounds **2** and **8** satisfy the drug-likeness criteria without violating the Lipinski [81], Veber [84], and Egan rules. Furthermore, based on these results, these compounds are not expected to cause problems with regard to oral bioavailability and pharmacokinetic parameters.

## 4. Materials and Methods

### 4.1. Synthesis of Compounds ***1***–***14***

Compounds **1**–**4** were synthesized by alkylation of methyl *p*-coumarate according to the methodology employed by Patra and coworkers [85] with modifications (Scheme 1). In this procedure, methyl *p*-coumarate (0.5 mmol) was added to a 25 mL flask equipped with a magnetic stirrer bar. Next, 15 mL toluene was added. The mixture was cooled to 0 °C, and 1.5 mmol NaH was added in portions. After 15 min, 1.5 mmol (153 µL) of prenyl bromide was added dropwise. The reaction progress was monitored through TLC using a Hex:EtOAc 8:2 (*v*/*v*) solution as the eluent. After 24 h, the solvent was removed under reduced pressure on a rotary evaporator, and the reaction mixture was then extracted with EtOAc (3 × 15 mL). The organic phase was washed with saturated NaCl solution (15 mL), dried over MgSO_4,_ and filtered off. The solvent was removed by evaporation at reduced pressure on a rotaevaporator. The compounds were isolated by column chromatography with gradient elution, starting with 100% hexane and changing to Hex:EtOAc 9.8:0.2 (*v*/*v*) after the separation of the first compounds. The resulting solids were dried under a vacuum to yield compounds **1** (32% yield), **2** (18% yield), **3** (15% yield), and **4** (35% yield) (Scheme 1).

Compounds **5**, **6**, and **7** were obtained from a hydrolysis reaction according to the methodology described by Uto and co-workers [86]. In this procedure, a solution of KOH (15 mL of a 10% aqueous) was added to a solution of the methyl esters **1, 2,** and **4** in MeOH (15 mL) (Scheme 1). The mixture was heated under reflux for 1 h, cooled to 0–5 °C, and acidified with 1 mol/L HCl. After removing MeOH under reduced pressure, the aqueous residue was extracted with EtOAc (3 × 15 mL). The organic phase was washed with saturated NH_4_Cl and brine, dried over MgSO_4_, and evaporated under reduced pressure. The resulting white solids were dried under a vacuum to afford compounds **5**, **6**, and **7** in a 100% yield.

Compounds **8**, **9**, **12**, **13**, and **14** were obtained from **2**, **4**, **5**, **6**, and **7** by catalytic hydrogenation. In this procedure, compounds **2**, **4**, **5**, **6**, and **7** and Pd/C (catalyst) were wholly dissolved in HPLC-grade EtOAc and transferred to a high-pressure reactor under stirring and kept under an H_2_ atmosphere and 400 psi at room temperature for 1–2 h (Scheme 1). The resulting oil was dried under a vacuum to yield compounds **8**, **9**, **12**, **13**, and **14** in a 100% yield.

Compounds **10** and **11** were synthesized according to the methodology reported by Kantee and co-workers [87] with some modifications (Scheme 1). In this procedure, LiAlH_4_ (0.68 mmol) was quickly added to a solution of compounds **3** and **4** (0.34 mmol) in THF (5.5 mL) at 0 °C. The reaction mixture was stirred under an N_2_ atmosphere at 0 °C for 1 h. After one hour, the mixture was stirred at room temperature for 9–10 h. The reaction mixture was then augmented with H_2_O, conc. HCl and extracted with EtOAc (x3). The combined organic layers were washed with brine, dried over MgSO_4_, and concentrated under a vacuum. Purification of the crude residue by column chromatography with isocratic elution with Hex/EtOAc 9.8:0.2 (*v*/*v*) gave the corresponding alcohol derivative. The resulting compounds were dried under a vacuum to yield compounds **10** (65% yield) and **11** (89% yield).

### 4.2. Antibacterial Assays

The in vitro antimicrobial action of artepillin C (**5**), drupanin (**6**) and derivatives **1**–**4**, and **7**–**14** was evaluated in terms of their minimum inhibitory concentration (MIC) values [88], which were interpreted as the lowest concentrations that inhibited bacterial growth. To this end, *Streptococcus mutans* (ATCC 25175), *Streptococcus mitis* (ATCC 49456), *Streptococcus salivarius* (ATCC 25975), *Streptococcus sanguinis* (ATCC 10556), *Streptococcus sobrinus* (ATCC 33478), *Enterococcus faecalis* (ATCC 4082), and *Lactobacillus paracasei* (ATCC 11578) were assayed by the broth microdilution method in 96-well microplates. The bacterial colonies were cultured at 37 °C for 24 h in blood agar (Difco Labs, Detroit, MI, USA). Further standardization of the inoculum quantity was accomplished on a Femto spectrophotometer (São Paulo, Brazil) operating at a wavelength of 625 nm to match 0.5 on the McFarland scale (1.5 × 10^8^ CFU/mL). The microorganism suspensions were diluted to a final concentration of 5 × 10^5^ CFU/mL. Samples of compounds **1**–**14** were dissolved in DMSO (Merck, Darmstadt, Germany) and tryptic soy broth (TSB, Difco) to obtain final concentrations varying from 0.98 to 2000 μg/mL. Inoculated microplate wells containing DMSO (1%) and TSB (1:5 *v/v* and 100%) were employed as the negative control. A non-inoculated well was also added to ensure the medium sterility. Chlorhexidine (Sigma-Aldrich, St. Louis, MO, USA) was used as the positive control at concentrations ranging from 0.115 to 59 μg/mL in TSB (Difco). The microplates were sealed with plastic film and incubated at 37 °C for 24 h. Next, 30 μL of revealing 0.02% resazurin (Sigma-Aldrich, St. Louis, MO, USA) was added to each microplate well to indicate the microbial viability. Before resazurin was added and to determine the MBC, a 10 µL aliquot of the inoculum was aseptically removed from each well and plated onto blood agar (Difco). The plates were incubated as described previously. The minimum bactericidal concentration (MBC) was determined as the lowest compound concentration that killed > 99.9% of the initial bacteria population, at which point no visible bacterial growth occurred [89]. The MIC values were assessed by analysis of the color change of the resazurin solution from blue (without metabolic activity) to pink (with metabolic activity) [90]. The MIC and MBC were determined in triplicate for each microorganism, and the results are presented as the mean of three replicates.

### 4.3. Computational Methodology

#### 4.3.1. Ligands and Targets Preparation

The 3D structures of the most active compounds **2** and **8** (Scheme 1) were optimized using the semi-empirical method AM1 [91], which was implemented in Hyperchem 8.0.8 software (Hypercube Inc., Gainesville, FL, USA). Next, the 3D structures were converted into .mdb format for use as input in the MOE-docking.

The crystal structures of *S. mitis* (**3LE0**) [46], *S. sanguinis* (**4N82**) [47], and *S. mutans* (**3AIC**) [48] were selected as antibacterial targets, which were downloaded from the Protein Data Bank (http://www.rcsb.org/, accessed on 8 June 2024). Some information related to the target structures is provided in Table 1.

#### 4.3.2. Docking Method Protocol and Validation

The molecular-docking studies were carried out to identify the binding interactions of the most active compounds **2** and **8** within the binding site residue of the targets by using MOE software [92], and the docking protocol steps were followed and detailed in our previous research [93,94] by using the following default parameters: placement: Triangle Matcher; rescoring 1: London dG scoring function. The visualization of all the possible interactions that have been formed between the compounds and receptor active site residues was generated by the BIOVIA DS visualize package (Dassault Systèmes BIOVIA, Discovery Studio Modeling Environment, 2020).

The re-docking of all the native ligands to their targets was conducted using “Dock Option” implemented in the MOE software [92] to validate the used method. The RSMD values of the obtained complexes (targets–crystallized ligands) were less than 2.50 Å [95], meaning that the docking method is accurate and successful.

#### 4.3.3. MD Simulations 

MD simulations were carried out using the “Compute/Simulations/Dynamics” options in the MOE 2014.0901 software [92] to study the stability of the two best-formed complexes. The following default parameters were used: the Nose–Poincaré–Andersen (NPA) algorithm to search the interactions of different residues in each system, and the MMFF94x force field for the energy minimization step of these complexes [96,97]. The MD protocol was settled for 1500 ps (an equilibrium period of 100 ps, followed by a production period of 1400 ps, at a constant temperature of 310 K). Finally, the plots of the potential energies U (kcal/mol) variations as a function of time t (ps) were produced using OriginPro 9.1.software [98].

#### 4.3.4. ADMET Evaluation

The drug-likeness rules, namely Lipinski, Veber, and Ghose, were verified by calculating the different physicochemical parameters (total polar surface area (TPSA); number of rotatable bonds (nROTB), molecular weight (MW), lipophilicity (LogP), number of hydrogen bond acceptors (nHA), and number of hydrogen bond donors (nHD)) using the SwissADME server (http://www.swissadme.ch/, accessed on 8 June 2024) [99].

The pkCSM server (http://biosig.unimelb.edu.au/pkcsm/prediction, accessed on 8 June 2024) [100] was used for the analysis of the ADMET profiles by calculating the following parameters: the absorption (Caco-2: colon adenocarcinoma, HIA: human intestinal absorption), distribution (CNS: central nervous system permeability, BBB: blood–brain barrier permeability), metabolism (CYP1A2 inhibitor, CYP2C19 inhibitor, CYP2D6 inhibitor), excretion (renal OCT2 substrate: organic cation transporter 2, total clearance), and toxicity (hERG: human ether-a-go-go-related gene, hepatotoxicity).

## 5. Conclusions

Plicatin B (**2**) was identified as a compound with promising activity against oral bacteria, including *S. mutans*, one of the main causative agents of bacteria caries. Moreover, this study revealed plicatin B to be more active than artepillin C, which has been highlighted as the main factor responsible for the antimicrobial activity of the Brazilian green propolis. However, among all the tested compounds, the most remarkable activity against *S. mutans* was found for compound **8**, which was obtained by hydrogenation of plicatin B. 

The molecular-docking/dynamics simulation results proved that compounds **2** and **8** have high binding affinities against three targets, *S. sanguinis* (**4N82**) and *S. mitis* (**3LE0**), as confirmed by the low energy values and various interactions with the active site residues of these targets. The ADMET properties were predicted for all the candidates to validate the pharmacodynamics and pharmacokinetics profiles, and these compounds verified the three rules, Lipinski, Veber, and Egan. In summary, the results of the present research revealed that the most active compounds **2** and **8** should be further investigated to explore their anti-cariogenic properties**,** including evaluation of their cytotoxicity, shelf life, anti-demineralization, remineralization, and cariostatic properties. However, the initial efforts must be focused on optimizing the synthesis of compounds **1**–**4,** aiming to reduce the reaction time and to make the isolation of the prenylated compounds **1**, **2**, **3**, and **4** from the reaction mixture simpler and faster. 

## Figures and Tables

**Table 1:** Minimum inhibitory concentration (MIC) and minimum bactericidal concentration (MBC) values (µg/mL) of compounds **2**–**9**, and **11**–**13** *.

Compound	*Enterococcus* *Faecalis* ATCC 4082	*Lactobacillus* *Paracasei* ATCC 11578	*Streptococcus Salivarius* ATCC 25975	*Streptococcus Sobrinus* ATCC 33478	*Streptococcus Mitis* ATCC 49456	*Streptococcus Sanguinis* ATCC 10556	*Streptococcus Mutans* ATCC 25175
**2**	500 (2.03) 500 (2.03)	500 (2.03) 1000 (4.06)	62.5 (0.25) 250 (1.01)	62.5 (0.25) 62.5 (0.25)	31.2 (0.13) 31.2 (0.13)	31.2 (0.13) 31.2 (0.13)	31.2 (0.13) 31.2 (0.51)
**3**	>2000 (>5.23) >2000 (>5.23)	>2000 (>5.23) >2000 (>5.23)	2000 (5.23) >2000 (>5.23)	2000 (5.23) >2000 (>5.23	500 (1.31) 1000 (2.61)	1000 (2.61) 2000 (5.23)	>2000 (5.23) >2000 (5.23)
**4**	>2000 (8.12) >2000 (>8.12)	>2000 (8.12) >2000 (>8.12)	>2000 (8.12) >2000 (>8.12)	>2000 (8.12) >2000 (>8.12)	1000 (4.06) 2000 (8.12)	>2000 (8.12) >2000 (>8.12)	>2000 (8.12) >2000 (>8.12)
**5**	>2000 (>6.66) >2000 (>6.66)	500 (1.66) 1000 (3.33)	1000 (3.33) 2000 (6.66)	500 (1.66) 2000 (6.66)	125 (0.41) 1000 (3.33)	500 (1.66) 1000 (3.33)	500 (1.66) 2000 (6.66)
**6**	1000 (3.09) 1000 (3.09)	500 (1.55) 500 (1.55)	250 (0.77) 500 (1.55)	125 (0.39) 125 (0.39)	250 (0.77) 250 (0.77)	250 (0.77) 250 (0.77)	125 (0.39) 250 (0.77)
**7**	500 (2.15) >2000 (>8.61)	1000 (4.31) 2000 (8.61)	1000 (4.31) 2000 (8.61)	1000 (4.31) 1000 (4.31)	500 (2.15) 500 (2.15)	500 (2.15) 500 (2.15)	1000 (4.31) 2000 (8.61)
**8**	125 (0.50) 125 (0.50)	62.5 (0.25) 250 (1.0)	62.5 (0.25) 62.5 (0.25)	125 (0.50) 125 (0.50)	31.25 (0.12) 31.25 (0.12)	62.5 (0.25) 62.5 (0.25)	62.5 (0.25) 62.5 (0.25)
**9**	>2000 (8.0) >2000 (8.0)	>2000 (8.0) >2000 (8.0)	1000 (4.0) 2000 (8.0)	1000 (4.0) 1000 (4.0)	500 (2.0) 1000 (4.0)	1000 (4.0) 1000 (4.0)	2000 (8.0) 2000 (8.0)
**11**	2000 (9.16) >2000 (>9.1)	2000 (9.16) >2000 (>9.1)	62.5 (0.29) 125 (0.57)	2000 (9.16) 2000 (9.16)	500 (2.29) 1000 (4.58)	1000 (4.58) 2000 (9.16)	1000 (4.58) 2000 (9.16)
**12**	1000 (3.26) 2000 (6.53)	500 (1.63) 1000 (3.26)	500 (1.63) 500 (1.63)	250 (0.82) 500 (1.63)	62.5 (0.20) 125 (0.41)	500 (1.63) 1000 (3.26)	500 (1.63) 500 (1.63)
**13**	>2000 (8.46) >2000 (8.46)	>2000 (8.46) >2000 (8.46)	>2000 (8.46) >2000 (8.46)	>2000 (8.46) >2000 (8.46)	250 (1.06) 1000 (4.23)	1000 (4.23) 1000 (4.23)	>2000 (8.46) >2000 (8.46)
CHD	3.69 (7.30) 3.69 (7.30)	1.84 (3.64) 1.84 (3.64)	0.92 (1.82) 0.92 (1.82)	1.84 (3.64) 1.84 (3.64)	3.69 (7.30) 3.69 (7.30)	3.69 (7.30) 3.69 (7.30)	0.92 (1.82) 0.92 (1.82)

* Compounds **1, 10,** and **14** were inactive (MIC > 1000 µg/mL) against all the tested bacteria and were not included). CHD: chlorhexidine. Values between parenthesis are expressed in mM. MBC values are given in blue.

**Table 2:** Some information related to the studied targets.

	3LE0	4N82	3AIC
Method	X-ray diffraction	X-ray diffraction	X-ray diffraction
Microorganism	*S. mitis*	*S. sanguinis*	*S. mutans*
Chain	A	A, B, C, D, E, F	A, B, C, D, E
Sequence length	153	178	488
Resolution (Å)	1.91	1.88	3.11
Native ligands	GOL	FMN	ACA

GOL: glycerol; FMN: flavin mononucleotide; ACA: acarbose.

**Table 3:** Docking results of compounds **2** and **8 when** docked into the *S. mitis* target (**3LE0**).

	S-Score (kcal/mol)	RMSD (Å)	Bonds between Atoms of Compounds and Active Site Residues				
			Atom of Compound	Involved Receptor Atoms	Involved Receptor Residues	Category	Type of Interaction
**2**	−4.228	2.997	O	HH11	ARG120(A)	HB	Conventional HB
			H	NE2	HIS85(A)	HB	Conventional HB
			/	NH2	ARG112(A)	Electrostatic	Pi-Cation
			C	/	TYR62(A)	Hydrophobic	Pi-Pi T-shaped
			C	/	VAL117(A)	Hydrophobic	Alkyl
**8**	−4.476	2.932	H	NE2	HIS85(A)	HB	Conventional HB
			H	OD1	ASP114(A)	HB	Carbon HB
			H	OD2	ASP77(A)	HB	Carbon HB
			H	OD1	ASP114(A)	HB	Carbon HB
			/	NH2	ARG112(A)	Electrostatic	Pi-Cation
			C	/	VAL117(A)	Hydrophobic	Alkyl
GOL	−3.655	2.149	O1	HH11	ARG120(A)	HB	Conventional HB
			O3	HH11	ARG112(A)	HB	Conventional HB
			H12	NE2	HIS85(A)	HB	Carbon H-Bond

HB: hydrogen bond. GOL: glycerol.

**Table 4:** Physicochemical properties and drug-likeness predictions of compounds **2** and **8**.

	Physicochemical Properties						Drug-Likeness Rules		
	TPSA (Å^2^)	n-ROT	MW (g/mol)	MLogP	n-HA	n-HD	Lipinski	Veber	Egan
				WLogP					
	(0~140)	(0~11)	(100~500)	(0~5)	(0~12)	(0~7)			
**2**	39.42	2	225.25	1.49	3	0	Accepted	Accepted	Accepted
				2.40					
**8**	30.19	1	223.27	2.33	2	0	Accepted	Accepted	Accepted
				3.01					

TPSA: topological polar surface area; n-ROT: number of rotatable; MW: molecular weight; Log P: logarithm of partition coefficient of compound between *n*-octanol and water; n-HA: number of hydrogen bond acceptors; n-HD: number of hydrogen bonds donors.

**Table 5:** ADMET/pharmacokinetic properties of compounds **2** and **8**.

*ADMET*	Parameters	Compounds	
		2	8
Absorption	Caco_2_ (Log P_app_ × 10^−6^ cm/s)	1.178	1.417
	HIA (%)	99.207	97.682
Distribution	CNS (logPS)	−1.800	−1.692
	BBB (logBB)	0.311	0.030
Metabolism	CYP1A2 inhibitor	Yes	Yes
	CYP2C19 Inhibitor	No	No
	CYP2D6 inhibitor	No	No
	CYP2D6 substrate	No	No
	CYP3A4 substrate	No	No
Excretion	Renal OCT2 substrate	No	No
	Total clearance (log mL/min/kg)	0.744	0.780
Toxicity	hERG I and II inhibitors	No	No
	Hepatotoxicity	No	No

**Caco-2**: colon adenocarcinoma, **HIA:** human intestinal absorption, **CNS:** central nervous system permeability, **BBB:** blood–brain barrier permeability, **Renal OCT2 substrate:** organic cation transporter 2, **hERG:** human ether-a-go-go-related gene.

## Data Availability

A draft of this paper is available as a preprint at https://chemrxiv.org/engage/chemrxiv/article-details/65a42dbe9138d23161e85cb4 (accessed on 17 January 2024).

## Supplementary Materials

The following supporting information can be downloaded at https://www.mdpi.com/article/10.3390/antibiotics13080787/s1, Tables S1 and S2: Docking results of compounds **2** and **8** docked into the *S. sanguinis* (**4N82**) and *S. mutans* (**3AIC**) targets. Figures S1–S56: ^1^H and ^13^C NMR spectra, DEPT 135 spectra, and ESI-TOF-MS spectra of compounds **1**–**14**.
