# Supplementary material for: In Vitro and In Silico Studies of the Antimicrobial Activity of Prenylated Phenylpropanoids of Green Propolis and Their Derivatives against Oral Bacteria
Source: Antibiotics (Basel). 2024 Aug 22;13(8):787. doi: 10.3390/antibiotics13080787 (PMC11352038; doi:10.3390/antibiotics13080787)
Supplement: Supplementary file 1 [file antibiotics-13-00787-s001.zip › antibiotics-3119786-supplementary.pdf]

Supplementary Material

In Vitro and In Silico Studies of the Antimicrobial Activity of Prenylated Phenylpropanoids of Green Propolis and Their Derivatives against Oral Bacteria

Tatiana M. Vieira [1,†], Julia G. Barco [1,†], Sara L. de Souza [2,†], Anna L. O. Santos [2,†], Ismail Daoud [3,4,†], Seyfeddine Rahalie [5,†], Noureddine Amdouni [6,†], Jairo K. Bastos [7], Carlos H. G. Martins [2], Ridha Ben Said [5,6,*] and Antônio E. M. Crotti [1,*]

[1] Department of Chemistry, Faculty of Philosophy, Science and Letters at Ribeirão Preto, University of São Paulo, Ribeirão Preto 14040-901, SP, Brazil; tati.manzini@gmail.com (T.M.V.); juliagrassibarco@usp.br (J.G.B.)

[2] Department of Microbiology, Institute of Biomedical Sciences, Federal University of Uberlândia, Uberlândia 38405320, MG, Brazil; sara.souza1905@gmail.com (S.L.d.S.); annaolvsantos@gmail.com (A.L.O.S.); carlos.martins2@ufu.br (C.H.G.M.)

[3] Department of Matter Sciences, University Mohamed Khider, BP 145 RP, Biskra 07000, Algeria; i.daoud@univ-biskra.dz

[4] Laboratory of Natural and Bio-Active Substances, Faculty of Science, Tlemcen University, Tlemcen P.O. Box 119, Algeria

[5] Department of Chemistry, College of Science, Qassim University, Qassim 51452, Saudi Arabia; saif.rahali@gmail.com

[6] Laboratoire de Caractérisations, Applications et Modélisations des Matériaux, Faculté des Sciences de Tunis, Université Tunis El Manar, Tunis 1068, Tunisia; noureddine.amdouni@fst.utm.tn

[7] School of Pharmaceutical Sciences of Ribeirão Preto, University of São Paulo, Ribeirão Preto 14040-903, SP, Brazil; jkbastos@fcfrp.usp.br

* Correspondence: ben.said.ridha@gmail.com (R.B.S.); millercrotti@ffclrp.usp.br (A.E.M.C.)

† These authors contributed equally to this work.

Table S1. Docking results of compounds **2** and **8** docked into *S. sanguinis* target (**4N82**).

	S-Score (kcal/mol)	RMSD (Å)	Bonds between atoms of compounds and active site residues				
			Atom of compound	Involved receptor Atoms	Involved receptor Residues	Category	Type of interaction
**2**	-6.156	2.071	O	H	ASN104(A)	HB	Conventional HB
			O	HA3	GLY103(A)	HB	Carbon HB
			/	O	THR63(A)	Other	Pi-Lone Pair
			/	/	PHE107(A)	Hydrophobic	Pi-Pi Stacked
			C	/	MET132(A)	Hydrophobic	Alkyl
			C	/	PRO62(A)	Hydrophobic	Alkyl
			C	/	TYR64(A)	Hydrophobic	Alkyl
**8**	-5. 575	1.283	O	H	ASN104(A)	HB	Conventional HB
			H	O	LEU65(A)	HB	Carbon HB
			/	O	TYR63(A)	Other	Pi-Lone Pair
			/	/	PHE107(A)	Hydrophobic	Pi-Pi Stacked
			C	/	PRO62(A)	Hydrophobic	Alkyl
**FMN**	-6.671	1.034	O1P	H	LEU11(A)	HB	Conventional HB
			O3P	H	SER12(A)	HB	Conventional HB
			O3P	HG	SER12(A)	HB	Conventional HB
			O3P	H	GLY13(A)	HB	Conventional HB
			O3P	H	ASN14(A)	HB	Conventional HB
			O2P	H	THR15(A)	HB	Conventional HB
			O2P	HG1	THR15(A)	HB	Conventional HB
			O1P	HH	TYR64(A)	HB	Conventional HB
			HO3	OH	TYR64(A)	HB	Conventional HB
			HO4	O	SER102(A)	HB	Conventional HB
			/	/	PHE107(A)	Hydrophobic	Pi-Pi Stacked
			/	/	PHE107(A)	Hydrophobic	Pi-Pi Stacked
			/	/	PHE107(A)	Hydrophobic	Pi-Pi Stacked
			C7M	/	LEU65(A)	Hydrophobic	Alkyl
			C7M	/	PHE107(A)	Hydrophobic	Pi-Alkyl
			C8M	/	PHE107(A)	Hydrophobic	Pi-Alkyl

HB: hydrogen bond. FMN: flavin mononucleotide

Table S2. Docking results of compounds **2** and **8** docked into *S. mutans* target (**3AIC**).

	**S-Score (kcal/mol)**	**RMSD (Å)**	**Bonds between atoms of compounds and active site residues**				
			**Atom of compound**	**Involved receptor Atoms**	**Involved receptor Residues**	**Category**	**Type of interaction**
**2**	-5.049	2.296	O	HE2	HIS587(A)	HB	Conventional HB
			H	OD1	ASP909(A)	HB	Carbon HB
			H	OD1	ASP909(A)	HB	Carbon HB
			/	OE2	GLU515(A)	Electrostatic	Pi-Anion
			C	/	LEU382(A)	Hydrophobic	Alkyl
			C	/	HIS587(A)	Hydrophobic	Pi-Alkyl
			C	/	TYR610(A)	Hydrophobic	Pi-Alkyl
			C	/	TYR916(A)	Hydrophobic	Pi-Alkyl
			/	/	LEU433(A)	Hydrophobic	Pi-Alkyl
**8**	-5.042	2.622	H	OD1	ASN481(A)	HB	Carbon H-Bond
			H	O	GLU515(A)	HB	Carbon H-Bond
			/	OD2	ASP588(A)	Electrostatic	Pi-Anion
			C	/	TRP517(A)	Hydrophobic	Pi-Alkyl
			C	/	HIS587(A)	Hydrophobic	Pi-Alkyl
			C	/	HIS587(A)	Hydrophobic	Pi-Alkyl
			C	/	TYR916(A)	Hydrophobic	Pi-Alkyl
			C	/	TYR916(A)	Hydrophobic	Pi-Alkyl
**ACA**	-6.674	2.513	O2B	HH21	ARG475(A)	HB	Conventional HB
			O2B	HE2	HIS587(A)	HB	Conventional HB
			O3B	HE2	HIS587(A)	HB	Conventional HB
			H8	OD2	ASP424(A)	HB	Conventional HB
			H2	OD1	ASN481(A)	HB	Conventional HB
			H6	OE1	GLU515(A)	HB	Conventional HB
			H16	OD2	ASP588(A)	HB	Conventional HB
			H25	OD2	ASP477(A)	HB	Conventional HB
			H4	OD2	ASP909(A)	HB	Carbon HB
			H5	OE2	GLU515(A)	HB	Carbon HB
			H14	OE1	GLU515(A)	HB	Carbon HB
			H15	OD1	ASP477(A)	HB	Carbon HB
			H17	OD2	ASP588(A)	HB	Carbon HB
			H24	OD2	ASP909(A)	HB	Carbon HB
			H9	OD2	ASP588(A)	Electrostatic	Attractive Charge
			H8	OE2	GLU515(A)	Electrostatic	Attractive Charge
			H8	OD2	ASP588(A)	Electrostatic	Attractive Charge
			N4A	OD1	ASP477(A)	Electrostatic	Attractive Charge

HB: hydrogen bond. ACA: acarbose (natural ligand).

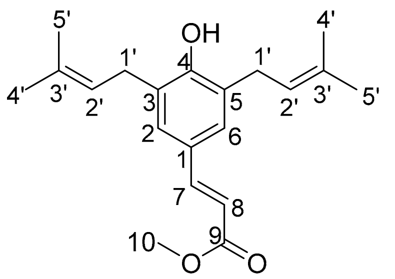

1

Methyl (*E*)-3,5-diprenyl-4-hydroxycinnamate (**1**). Yellowish oil, 32% yield. ^{1}H NMR (400 MHz, $CDCl_3$, **S1**): 1.76 (6H, *s*, H5’), 1.78 (6H, *s*, H4’), 3.34 (4H, *d*, $J_{1',2'}$=7.2 Hz, H1’), 3.78 (3H, *s*, H10), 5.30 (2H, *m*, H2’), 6.28 (1H, *d*, $J_{8,7}$=16.0 Hz, H8), 7.16 (1H, *s*, H2=H6), 7.60 (1H, *d*, $J_{7,8}$=16.0 Hz, H7). ^{13}C NMR (100 MHz, $CDCl_3$, **S2** and **S3**): 17.9 (CH_3, C5’), 25.8 (CH_3, C4’), 29.5 (CH_2, C1’), 51.5 (CH, C10), 115.1 (CH, C8), 121.5 (CH, C2’), 126.8 (C, C3=C5), 127.8 (C, C1), 128.1 (CH, C2=C6), 134.9 (C, C3’), 145.4 (CH, C7), 155.1 (C, C4), 168.0 (C, C9). HR-ESI(+)-MS (Q-TOF, **S4**): Found *m/z* 315.1941 (calc. for $C_{20}H_{27}O_3^+$, *m/z* 315.1955, error +4.4); *m/z* 337.1758 (calc. for $C_{20}H_{23}NaO_3^+$, *m/z* 337.1774, error -4.1)**.** HPLC purity: 98.2%.

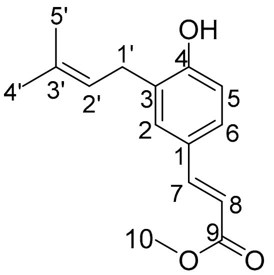

2

Methyl (*E*)-3-prenyl-4-Hydroxycinnamate (plicatin B, **2**). White solid, m.p. 45-48 ºC, 18% yield. ^{1}H NMR (400 MHz, $CDCl_3$, **S5**): 1.71 (3H, *s*, H5’), 1.75 (3H, *s*, H4’), 3.30 (2 H, *d*, $J_{1',2'}$ =7.3 Hz, H1’), 3.78 (3H, *s*, H10), 5.28 (1H, *m*, H2’), 6.27(1 H, *d*, $J_{8,7}$=16.0 Hz, H8), 7.03 (1H, *d*, $J_{5,6}$=8.4 Hz, H5), 7.20 (1H, *dd*, $J_{6,5}$=8.4 Hz. $J_{6,2}$=2.3 Hz, H6), 7.29 (1H, *d*, $J_{2,6}$=2.3 Hz, H2), 7.60 (1H, *d*, $J_{7,8}$=16.0 Hz, H7). ^{13}C NMR (100 MHz, $CDCl_3$, **S6** and **S7**): 17.9 (CH_3, C5’), 25.8 (CH_3, C4’), 28.9 (CH_2, C1’), 51.5 (CH_3, C10), 115.0 (CH, C8), 118.0 (CH, C5), 122.3 (CH, C2’), 126.4 (CH, C6), 127.2 (C, C3), 127.4 (C, C1), 129.4 (CH, C2), 132.7 (C, C3’), 144.5 (CH, C7), 156.5 (C, C4), 167.9 (C, C9). HR-ESI(+)-MS (Q-TOF, **S8**): Found *m/z* 247.1322 (calc. for $C_{15}H_{19}O_3^+$, *m/z* 247.1329, error +2.8); *m/z* 269.1139 (calc. for $C_{15}H_{18}NaO_3^+$, *m/z* 269.1148, error +3.3). HPLC purity: 97.4%.

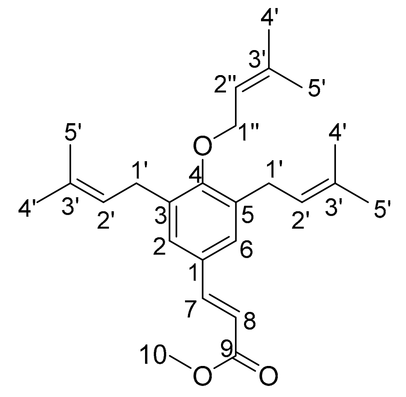

3

Methyl (*E*)-3,5-diprenyl-4-prenyloxycinnamate (3). Yellowish oil, 15% yield. ^{1}H NMR (400 MHz, $CDCl_3$, **S9**): 1.72 (12 H, *s*, H4'=H5'), 1.78 (6 H, *s*, H4"=H5"), 3.38 (4 H, *d*, *J*=6.4 Hz, H1'), 3.80 (3H, *s*, H10), 4.32 (2 H, *d*, *J* = 6.2 Hz, H1"), 5.28 (2H, *m*, H2'), 5.58 (1H, *m*, H2"), 6.30 (1H, *d*, $J_{8,7}$=16.0 Hz, H8), 7.19 (2H, *s*, H2=H6), 7.60 (1H, *d*, $J_{7,8}$=16.0 Hz, H7). ^{13}C NMR (100 MHz, $CDCl_3$, **S10** and **S11**): 18.0 (CH_3, C4'=C5'), 25.9 (CH_2, C4"=C5"), 28.5 (CH, C1'), 51.7 (CH_3, C10), 70.6 ($C4H_2$, C1"), 116.4 (CH, C8), 120.5 (CH, C2"), 122.7 (CH, C2'), 127.8 (C, C1), 128.2 (CH, C2=C6), 130.3 (C, C3=C5), 135.7 (C, C3'), 137.9 (C, C3), 145.2 (C, C7"), 157.4 (C, C4) 167.8 (C, C9). HR-ESI(+)-MS (Q-TOF, **S12**): HR-ESI(+)-MS (Q-TOF, **S12**): Found *m/z* 383.2570 (calc. for $C_{25}H_{35}O_3^+$, *m/z* 383.2581, error +2.9). HPLC purity: 98.5%.

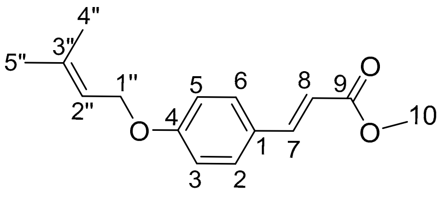

4

Methyl (*E*)-4-prenyloxycinnamate (4). Yellowish oil, 15% yield. ^{1}H NMR (400 MHz, $CDCl_3$, **S13**): 1.75 (3 H, *s*, H5"), 1.80 (3 H, *s*, H4"), 3.79 (3H, *s*, H10), 4.54 (2 H, *d*, $J_{1',2'}$ = 6.7 Hz, H1"), 5.48 (1 H, *m*, H2"), 6.32 (1H, *d*, $J_{8,7}$=16.0 Hz, H8), 6.91 (2H, *d*, $J_{3,2}$=8.7 Hz, H3=H5), 7.46 (2H, *d*, $J_{2,3}$=8.7 Hz, H2=H6), 7.65 (1H, *d*, $J_{7,8}$=16.0 Hz, H7). ^{13}C NMR (100 MHz, $CDCl_3$, **S14** and **S15**): 18.5 (CH_3, C5"), 26.1 (CH_3, C4"), 51.9 (CH_3, C10), 65.2 (CH_2, C1"), 115.1 (CH, C3=C5), 115.3 (CH, C8), 119.4 (CH, C2"), 127.2 (C, C1), 129.9 (CH, C2=C6), 138.8 (C, C3"), 144.8 (CH, C7), 161.0 (C, C4), 168.1 (C, C9). HR-ESI-MS (Q-TOF, **S16**): Found *m/z* 247.1327 (calc. for $C_{15}H_{19}O_3^+$, *m/z* 247.1329, error +0.8); *m/z* 269.1143 (calc. for $C_{15}H_{18}NaO_3^+$, *m/z* 269.1148, error -1.9). HPLC purity: 98.7%

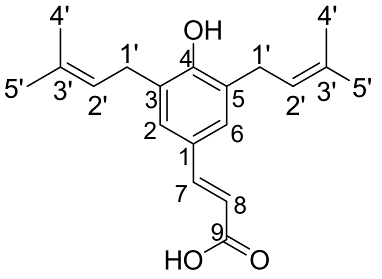

5

(*E*)-3,5-diprenyl-4-Hydroxycinnamic acid (artepillin C, **5**). White solid, m.p. 98-101 °C, 15% yield. ^{1}H NMR (400 MHz, $CDCl_3$, **S17**): 1.83 (6 H, *s*, H5'), 1.85 (6 H, *s*, H4'), 3.41 (4 H, *d*, *J*=6.4 Hz, H1'), 5.37 (2 H, *m*, H2'), 6.35 (1 H, *d*, $J_{8,7}$=16.0 Hz, H8), 7.31 (1H, *s*, H2=H6), 7.76 (1H, *d*, $J_{7,8}$=16.0 Hz, H7). ^{13}C NMR (100 MHz, $CDCl_3$, **S18** and **S19**): 18.0 (CH_3, C5'), 26.0 (CH_3, C4'), 29.6 (CH_2, C1'), 114.2 (CH, C8), 121.5 (CH, C2'), 126.6 (C, C3=C5), 127.9 (C, C1), 128.5 (CH, C2=C6), 135.4 (C, C3'), 147.6 (CH, C7), 155.6 (C, C4), 172.1 (C, C9). HR-ESI-MS (Q-TOF, **S20**): Found *m/z* 303.1585

(calc. for $C_{18}H_{23}O_4^+$, *m/z* 303.1591, error +2.0); *m/z* 325.1406 (calc. for $C_{18}H_{22}NaO_4^+$, *m/z* 325.1410, error +1.2). HPLC purity: 97.8 %

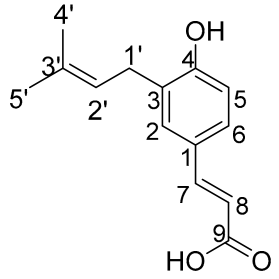

6

(*E*)-3-prenyl-4-hydroxycinnamic acid (drupanin A, **6**). White solid, m.p. 145-148 ºC, 100% yield. ^{1}H NMR (400 MHz, $CDCl_3$, **S21**): 1.78 (3 H, *s*, H5') 1.79 (3 H, *s*, H4') 3.36 (2 H, *d, J*=7.0 Hz, H1') 5.33 (1 H, *m*, H2') 6.32 (1 H, *d*, $J_{8,7}$=15.8 Hz, H8) 6.82 (1H, *d*, $J_{5,6}$=8.0 Hz, H5) 7.33 (2H, *dd*, $J_{6,5}$=8.0 Hz, $J_{6,2}$=2.0 Hz, H2 and H6), 7.71 (1H, *d*, $J_{7,8}$=15.8 Hz, H7). ^{13}C NMR (100 MHz, $CDCl_3$, **S22** and **S23**): 18.0 (CH_3, C5'), 25.9 (CH_3, C4'), 29.7 (CH_2, C1'), 114.4 (CH, C8), 116.4 (CH, C5), 121.2 (CH, C2'), 127.3 (CH, C6), 127.7 (C, C1), 128.3 (C, C3), 130.7 (CH, C2), 131.4 (C, C3'), 147.1 (CH, C7), 157.1 (C, C4), 177.4 (C, C9). HR-ESI-MS (Q-TOF, **S24**): Found *m/z* 233.1165 (calc. for $C_{14}H_{17}O_3^+$, *m/z* 233.1172, error +3.0); *m/z* 255.0980 (calc. for $C_{14}H_{16}NaO_3^+$, *m/z* 255.0992, error +4.7). HPLC purity: 98.7 %.

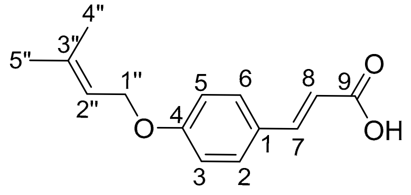

7

(*E*)-4-prenyloxycinnamic acid (7). White solid, m.p. 164-167 ºC, 100% yield. ^{1}H NMR (400 MHz, $CDCl_3$, **S25**): 1.75 (3H, s, H5"), 1.78 (3H, *s*, H4"), 4.55 (2H, *d, J*$_{1',2'}$=6.8 Hz, H1"), 5.48 (1H, *m*, H2"), 6.32 (1H, *d*, *J*=15.9 Hz, H8), 6.92 (1H, *d, J*=8.8 Hz, H3=H5), 7.50 (1H, *d, J*=8.7 Hz, H2=H6), 7.74 (1H, *d*, *J*=15.9 Hz, H7). ^{13}C NMR (100 MHz, $CDCl_3$, **S26** and **S27**): 18.4 (CH, C5"), 26.0 (CH, C4"), 65.2 (CH_2, C1"), 114.4 (CH, C3=C5), 115.4 (CH, C8), 119.5 (CH, C2"), 126.9 (C, C1), 130.1 (CH, C2=C6), 138.0 (C, C3"), 146.8 (CH, C7), 161.2 (C, C4), 174.1 (C, C9). HR-ESI-MS (Q-TOF, **S28**): Found *m/z* 233.1167 (calc. for $C_{14}H_{17}O_3^+$, *m/z* 233.1172, error +2.1); *m/z* 255.0988 (calc. for $C_{14}H_{16}NaO_3^+$, *m/z* 255.0992, error +1.6). HPLC purity: 96.4 %.

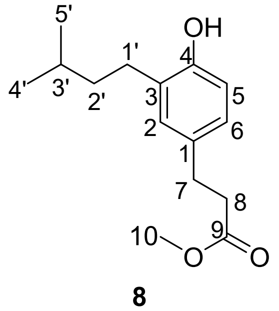

8

Methyl (*E*)-3-isopentyl-7,8-dihydro-4-hydroxycinnamate (**8**). Yellowish oil, 100% yield. ^{1}H NMR (400 MHz, $CDCl_3$, **S29**): 0.94 (3 H, *d*, $J_{4',3'}$= $J_{5',3'}$=6.5 Hz, H4'=H5'), 1.47 (2H, *m*, H2'), 1.60 (1 H, *m*, H3'), 2.58 (4H, *t*, *J*=8.1 Hz, H1' and H8), 2.85 (2H, *t*, $J_{7,8}$=8.1 Hz, H7), 3.67 (3H, *s*, H10), 6.68 (1H, *d*, $J_{5,6}$=8.1 Hz, H5), 6.85 (1H, *dd*, $J_{6,5}$=8.1 Hz, $J_{6,2}$=2.1 Hz, H6), 6.93 (1H, *d*, $J_{2,6}$=2.1, H2). ^{13}C NMR (100 MHz, $CDCl_3$,

S30 and **S31**): 22.6 (CH_3, C4'=C5'), 27.9 (CH, C3'), 28.0 (CH_2, C2') 30.3 (CH_2, C7), 36.2 (CH_2, C8), 39.0 (CH_2, C1'), 51.7 (CH_3, C10), 115.2 (CH, C5), 126.4 (CH, C6), 129.2 (C, C3), 129.8 (CH, C2), 132.1 (C, C1), 152.3 (C, C4), 173.9 (C, C9). HR-ESI-MS (Q-TOF, **S32**): Found *m/z* 251.1651 (calc. for $C_{15}H_{23}O_3^+$, *m/z* 251.1642, error -3.6); *m/z* 273.1473 (calc. for $C_{15}H_{22}NaO_3^+$, *m/z* 273.1461, error -4.4). HPLC purity: 98.4%.

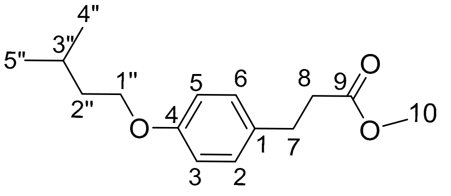

9

Methyl (*E*)-4-isopentyloxy-7,8-dihydrocinnamate (9). White solid, m.p. 172-176 °C, 100% yield. ^{1}H NMR (400 MHz, $CDCl_3$, **S33**): 1.38 (3H, *s*, H4"=H5"), 2.09 (2H, *m*, H2"), 2.25 (1H, *m*, H3"), 3.02 (2H, *t, J* =8.1 Hz, H8) 3.31 (2H, *t, J*=8.1 Hz, H7), 4.08 (3H, *s*, H10), 4.38 (2H, *t*, $J_{1'',2''}$=6.7 Hz, H1"), 7.24 (2H, *d, J*=8.4 Hz, H3=H5), 7.53 (2H, *d, J*=8.4 Hz, H2=H6). ^{13}C NMR (100 MHz, $CDCl_3$, **S34** and **S35**): 22.6 (CH_3, C4"=C5"), 25.1 (CH, C3"), 30.2 (CH_2, C2"), 36.1 (CH_2, C1"), 38.1 (CH_2, C8), 51.6 (CH_3, C10), 66.6 (CH_2, C7), 114.5 (CH, C3=C5), 129.2 (CH, C2=C6), 132.5 (C, C1), 157.7 (C, C4), 173.7 (C, C9). HR-ESI-MS (Q-TOF, **S36**): Found *m/z* 251.1649 (calc. for $C_{15}H_{23}O_3^+$, *m/z* 251.1642, error -2.8); *m/z* 273.1473 (calc. for $C_{15}H_{22}NaO_3^+$, *m/z* 273.1461, error -4.4). HPLC purity: 98.0%.

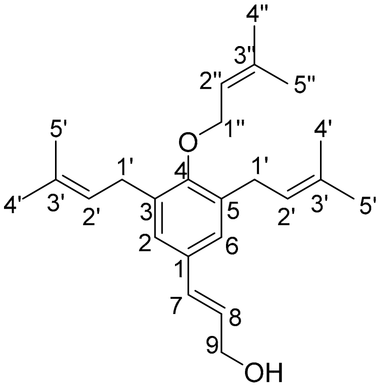

10

(*E*)-3,4-diprenyl-4-isoprenyloxycinnamic alcohol (10). Yellowish oil, 63% yield. ^{1}H NMR (400 MHz, $CDCl_3$, **S37**): 1.71 (9 H, *br s*), 1.74 (9 H, *br s*), 3.31 (4H, *d*,$J_{1',2'}$=7.2 Hz, H1'), 4.28 (2 H, *m*, H9), 4.52 (2 H, *t*, $J_{1'',2''}$=6.3 Hz, H1"), 5.30 (1 H, *m,* H2'), 5.49 (1H, *m*, H2"), 6.22 (1H, $J_{7,8}$ = 16.0 Hz, H7), 6.52 (1H, $J_{8,7}$ = 16.0 Hz, H8), 6.79 (2H, *br s*, H2=H6). ^{13}C NMR (100 MHz, $CDCl_3$, **S38** and **S39**): 18.4 (CH_3, C4'=C4"), 25.9 (CH_3, C5'=C5"), 28.6 (CH_2, C1'), 64.1 (CH_2, C9), 65.4 (CH_2, C1"), 120.2 (CH, C2"), 122.7 (CH, C2'), 127.5 (CH, C1), 127.7 (CH, C2=C6), 129.0 (CH, C8), 130.7 (C, C3'), 131.6 (CH, C7), 132.6 (C, C3=C5), 137.4 (C, C3"), 156.6 (C, C4). HR-ESI-MS (Q-TOF, **S40**): Found *m/z* 377.2463 (calc. for $C_{24}H_{34}NaO_2^+$, *m/z* 377.2451, error -3.2). HPLC purity: 99.1 %.

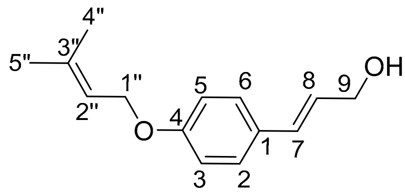

11

(*E*)-4-isoprenyloxycinnamic alcohol (11). Yellowish oil, 35% yield. ^{1}H NMR (400 MHz, $CDCl_3$, **S41**): 1.74 (3 H, *s*, H5”), 1.79 (3 H, *s*, H4”), 4.29 (2H, *dd*, *J*=5.9 and 1.3 Hz, H9), 4.51 (2 H, *d,* $J_{1”,2”}$=6.7 Hz, 1”), 5.49 (1 H, *m*, H2”), 6.24 (1 H, *dd,* $J_{8,7}$=15.8 Hz, $J_{8,9}$=5.9 Hz, H8), 6.55 (1H, *d,* $J_{7,8}$=15.8 Hz, H7), 6.87 (2H, *d*, $J_{5,6}$=$J_{3,2}$=8.7 Hz, H3=H5), 7.31 (2H, *dd*, $J_{2,3}$=8.7 Hz, $J_{2,6}$=1.9 Hz, H2=H6). ^{13}C NMR (100 MHz, $CDCl_3$, **S42** and **S43**): 18.5 (CH_3, C5”), 25.9 (CH_3, C4”), 64.1 (CH_2, C1”), 64.9 (CH, C9), 114.9 (CH, C2”), 119.6 (CH, C3=C5), 126.2 (CH, C8), 127.7 (C, C1), 129.4 (CH, C2=C6), 131.2 (CH, C7), 138.5 (C, C3”), 158.8 (C, C4). HR-ESI-MS (Q-TOF, **S44**): Found *m/z* 241.1183 (calc. for $C_{14}H_{18}NaO_2^+$, *m/z* 241.1189, error +4.1). HPLC purity: 97.8%.

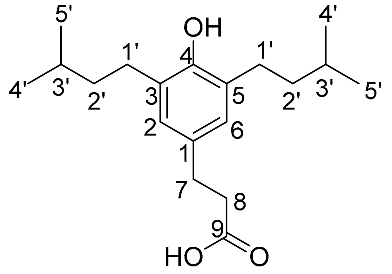

12

(*E*)-2,3-diisopentenyl-4-hydroxy-7,8-dihydrocinnamic acid (**12**). Yellowish oil, 100% yield. ^{1}H NMR (400 MHz, $CDCl_3$, **S45**): 0.95 (12H, *d, J* = 7.1 Hz, H4’=H5’), 1.48 (4H, *m*, H2’), 1.65 (2H, *m*, H3’), 2.50 (4H, *m*, H1’), 2.62 (2H, *t*, $J_{8,7}$= 7.5 Hz, H8), 2.86 (2H, *t,* $J_{7,8}$= 7.5 Hz, H7), 6.81 (1H, *s*, H2=H6). ^{13}C NMR (100 MHz, $CDCl_3$, **S46** and **S47**): 22.8 (CH_3, C4’=C5’), 28.36 (CH, C3’), 28.43 (CH_2, C2’), 30.4 (CH_2, C7), 34.5 (CH_2, C8), 39.3 (CH_2, C1’), 127.5 (C, C3=C5), 128.6 (CH, C2=C6), 132.2 (C, C1), 150.1 (C, C4), 177.5 (C, C9). HR-ESI-MS (Q-TOF, **S48**): Found *m/z* 307.2277 (calc. for $C_{19}H_{31}O_3^+$, *m/z* 307.2268, error -2.9); *m/z* 329.3075 (calc. for $C_{19}H_{30}NaO_3^+$, *m/z* 329.2087, error +3.6). HPLC purity: 98.5%.

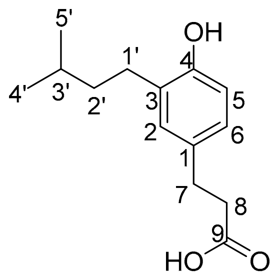

13

(*E*)-3-isopentenyl-4-hydroxy-7,8-dihydrocinnamic acid (**13**). Incolor oil, 100% yield. ^{1}H NMR (400 MHz, $CDCl_3$, **S49**): 0.94 (6H, *d*, *J*=6.9 Hz, H4’=H5’) 1.48 (2H, *m*, H2’), 1.61 (1H, *m*, H3’), 2.57 (2H, *m*, H8), 2.64 (2H, *t*, *J*= 8.1 Hz, H1’), 2.86 (2H, *t*, $J_{7,8}$ = 7.5 Hz, H7), 6.68 (1H, *d*, $J_{5,6}$ =8.0 Hz, C5), 6.89 (1H, *dd*, $J_{6,5}$=8.0 Hz, $J_{6,2}$=2.0 Hz, H6), 6.95 (1H, *d*, *J*=4.0 Hz, H2). ^{13}C NMR (100 MHz, $CDCl_3$, **S50** and **S51**): 22.8 (CH_3, C4’=C5’), 28.0 (CH. C3’), 28.2 (CH_2, C2’), 30.2 (CH_2, C7), 36.0 (CH_2, C8), 39.2 (CH_2, C1’), 115.4 (CH, C5), 126.7 (CH, C6), 129.2 (C, C3), 130.2 (CH, C2), 132.5 (C, C1), 152.2 (C, C4), 178.5 (C, C9). HR-

ESI-MS (Q-TOF, **S52**): Found *m/z* 237.1492 (calc. for $C_{14}H_{21}O_3^+$, *m/z* 237.1485, error -3.0); *m/z* 259.1313 (calc. for $C_{14}H_{20}NaO_3^+$, *m/z* 259.1305, error +3.1). HPLC purity: 99.0 %.

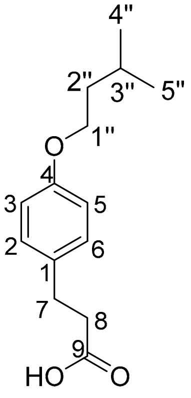

14

(*E*)-isopentyloxy-4-hydroxy-7,8-dihydrocinnamic acid (14). Yellowish oil, 100% yield.^{1}H NMR (400 MHz, $CDCl_3$, **S53**): 0.94 (3H, *s*, H5”), 0.96 (3H, *s*, H4”), 1.66 (2H, *q, J* = 6.7 Hz, H2”), 1.84 (1H, *m*, H3”), 2.65 (2H, *t, J*=8.0 Hz, H7), 2.89 (2H, *t, J*=8.0 Hz, H8), 3.96 (2H, *t, J*= 6.7 Hz, H1”), 6.83 (1H, *d, J*=8.5 Hz, H3=H5), 7.11 (1H, *d, J*=8.5 Hz, H2=H6). ^{13}C NMR (100 MHz, $CDCl_3$, **S54** and **S55**): 22.7 (CH, C4”=C5”), 25.2 (CH_2, C3”), 30.0 (CH_2, C7), 36.0 (CH_2, C8), 38.2 (CH_2, C2”), 66.7 (CH_2, C1”), 114.9 (CH, C3=C5), 129.4 (CH, C2=C6), 132.3 (C, C1), 157.9 (C, C4), 172.8 (C, C9). HR-ESI-MS (Q-TOF, **S56**): Found *m/z* 237.1475 (calc. for $C_{14}H_{21}O_3^+$, *m/z* 237.1485, error +4.2); *m/z* 259.1311 (calc. for $C_{14}H_{20}NaO_3^+$, *m/z* 259.1305, error -2.3). HPLC purity: 98.2%.

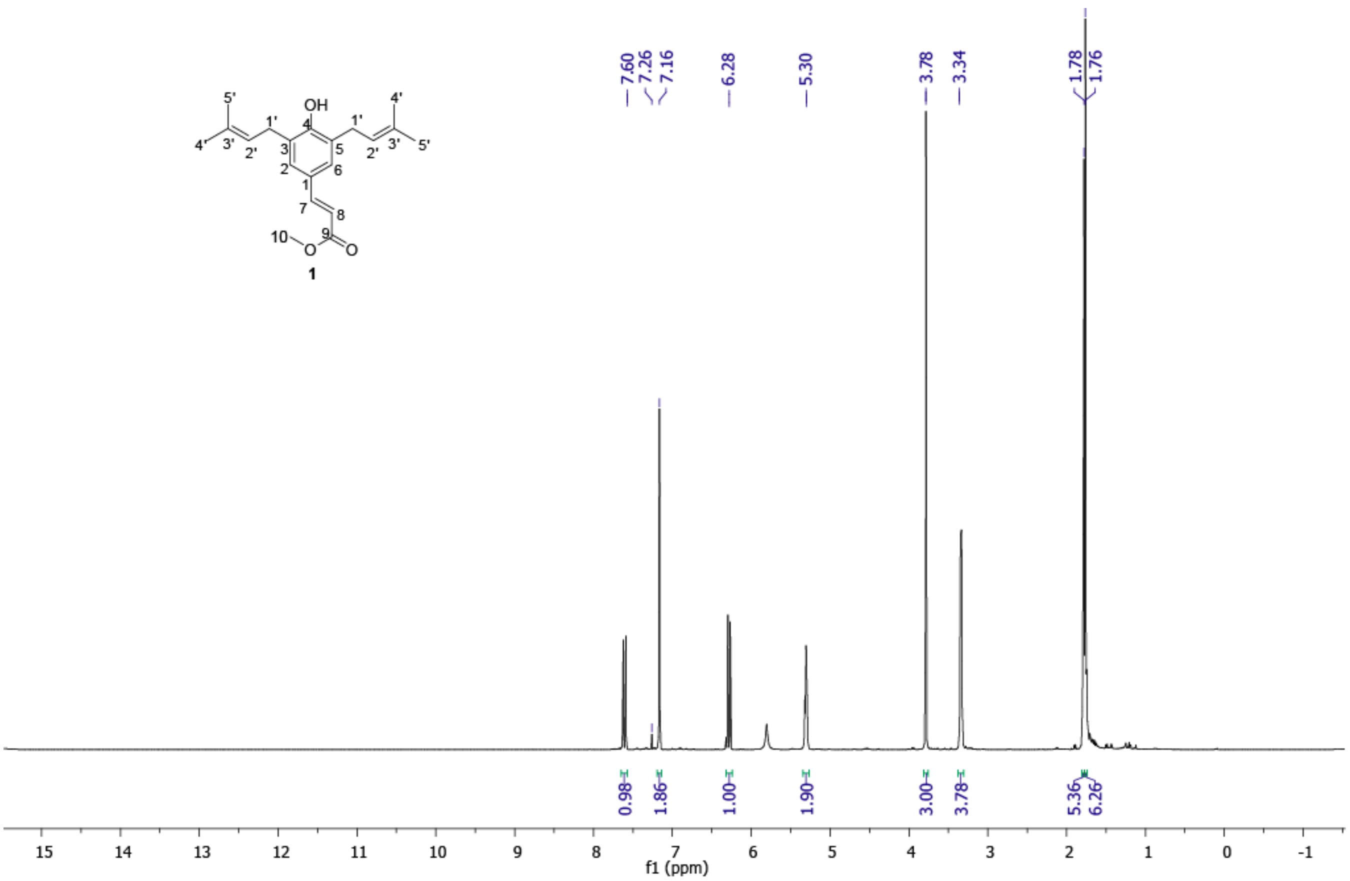

Figure S1. ^{1}H NMR spectrum of compound **1** (400 MHz, $CDCl_3$, TMS).

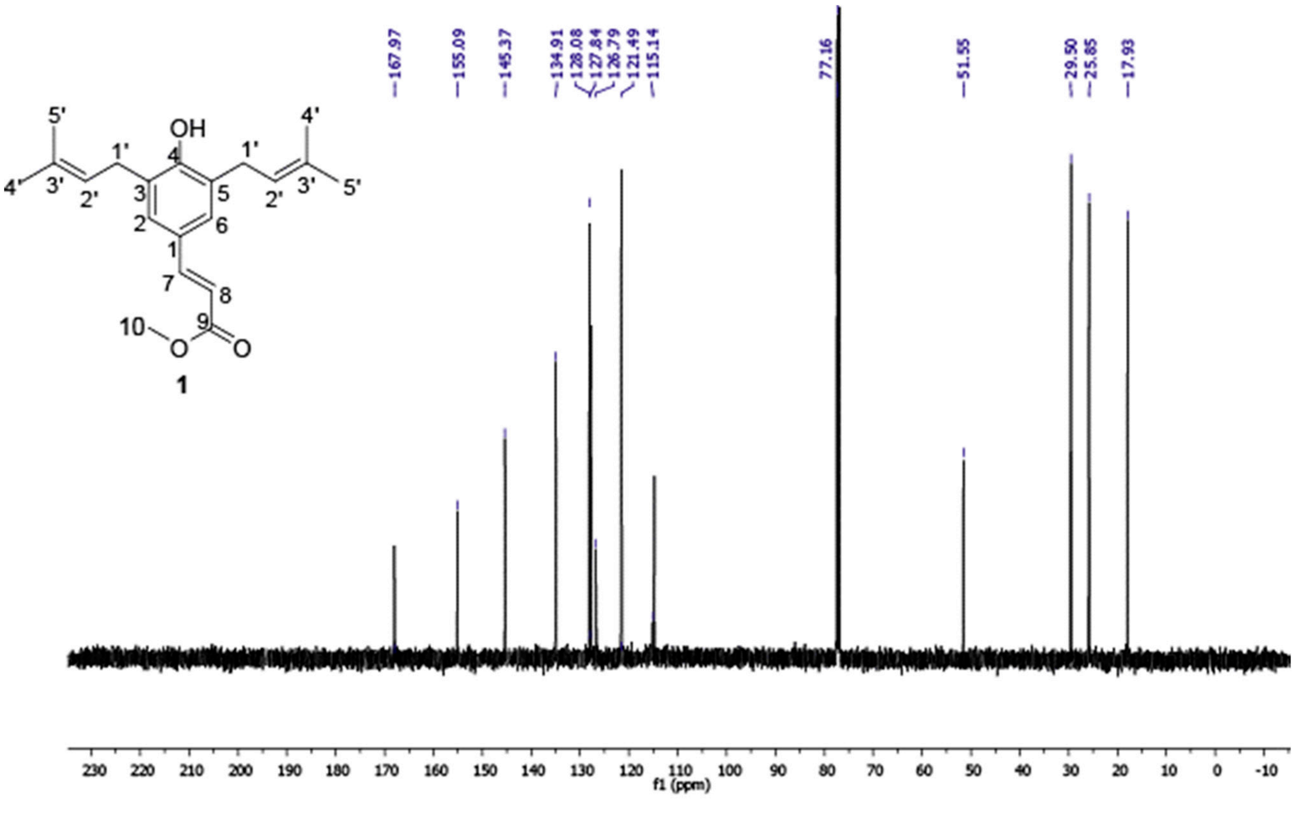

Figure S2. ^{13}C NMR spectrum of compound **1** (400 MHz, $CDCl_3$, TMS).

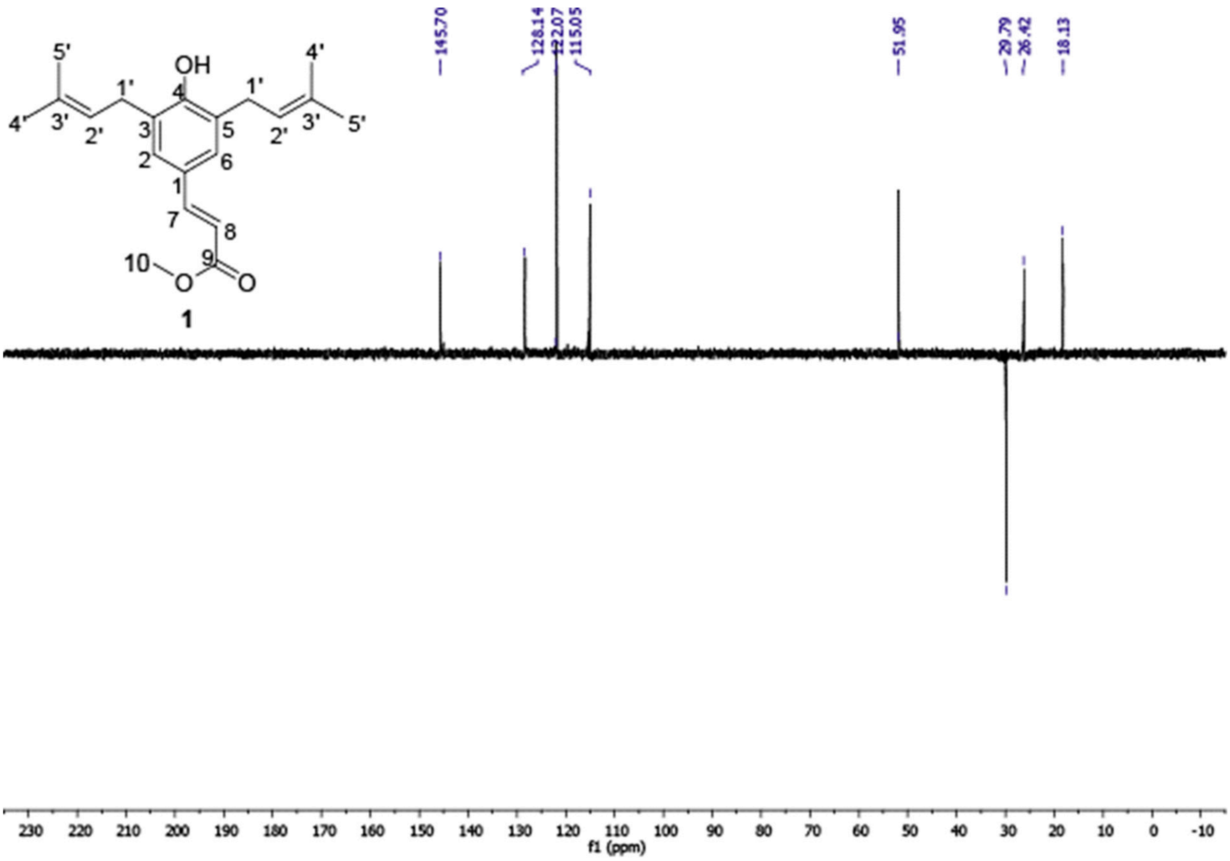

Figure S3. DEPT 135 spectrum of compound **1** (100 MHz, $CDCl_3$, TMS)

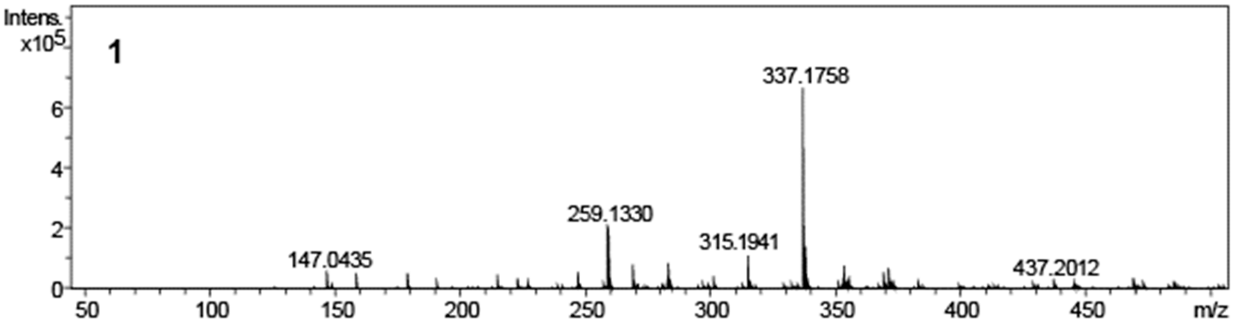

Figure S4. ESI(+)-MS spectrum of compound **1** (Q-TOF).

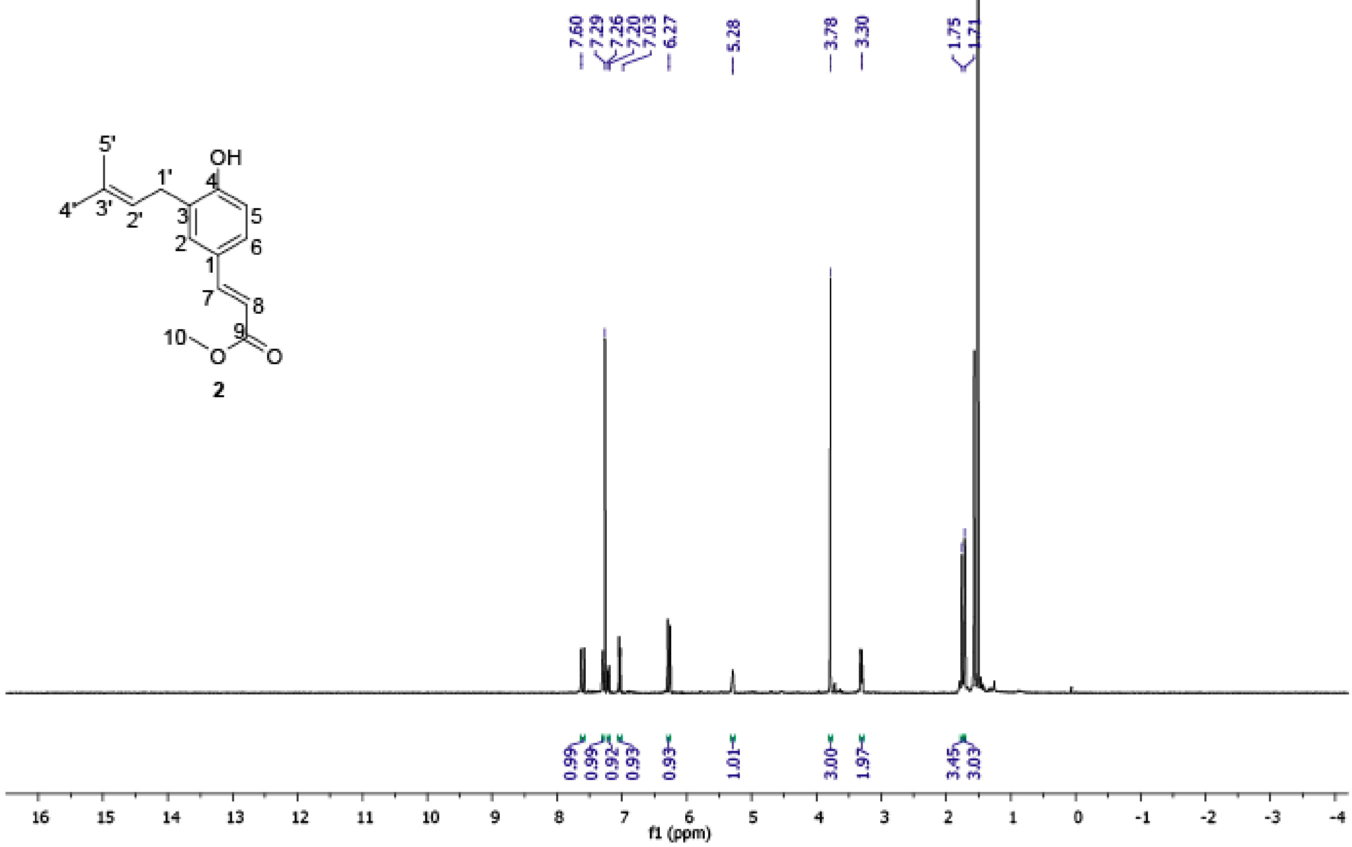

Figure S5. ^{1}H NMR spectrum of compound **2** (400 MHz, CDCl$_3$, TMS).

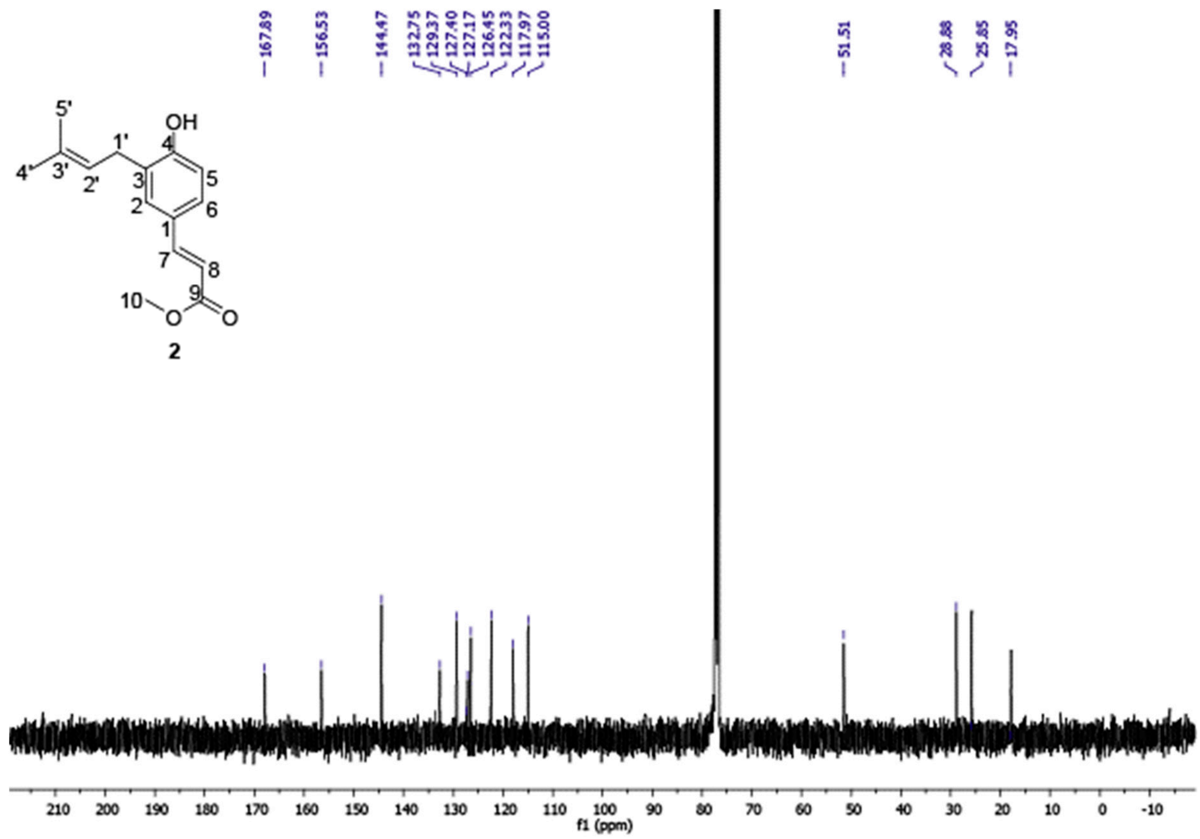

Figure S6. ^{13}C NMR spectrum of compound **2** (400 MHz, CDCl$_3$, TMS).

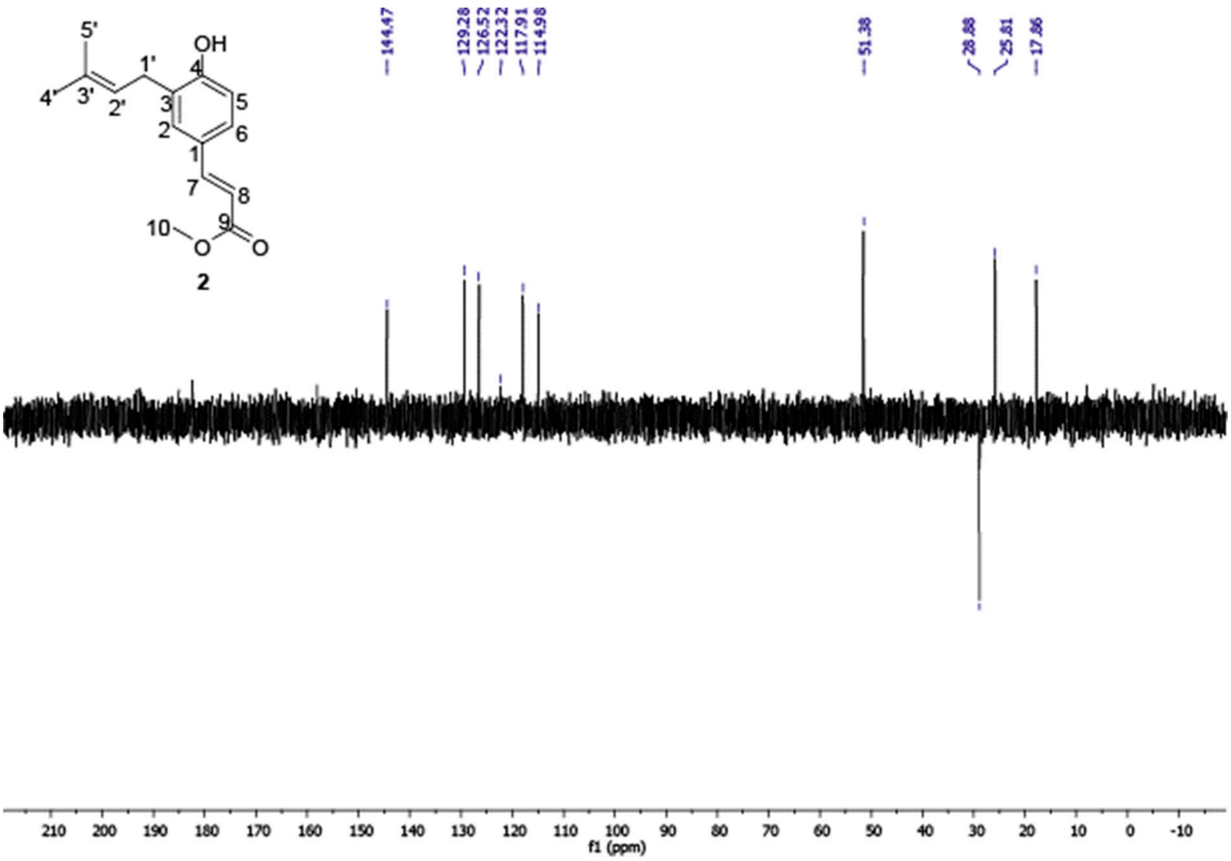

Figure S7. DEPT 135 spectrum of compound **2** (100 MHz, $CDCl_3$, TMS)

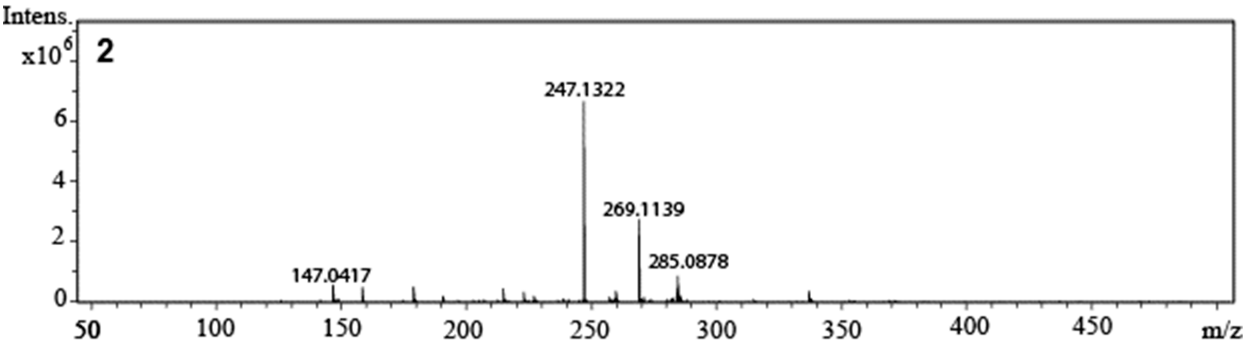

Figure S8. ESI(+)-MS spectrum of compound **2** (Q-TOF).

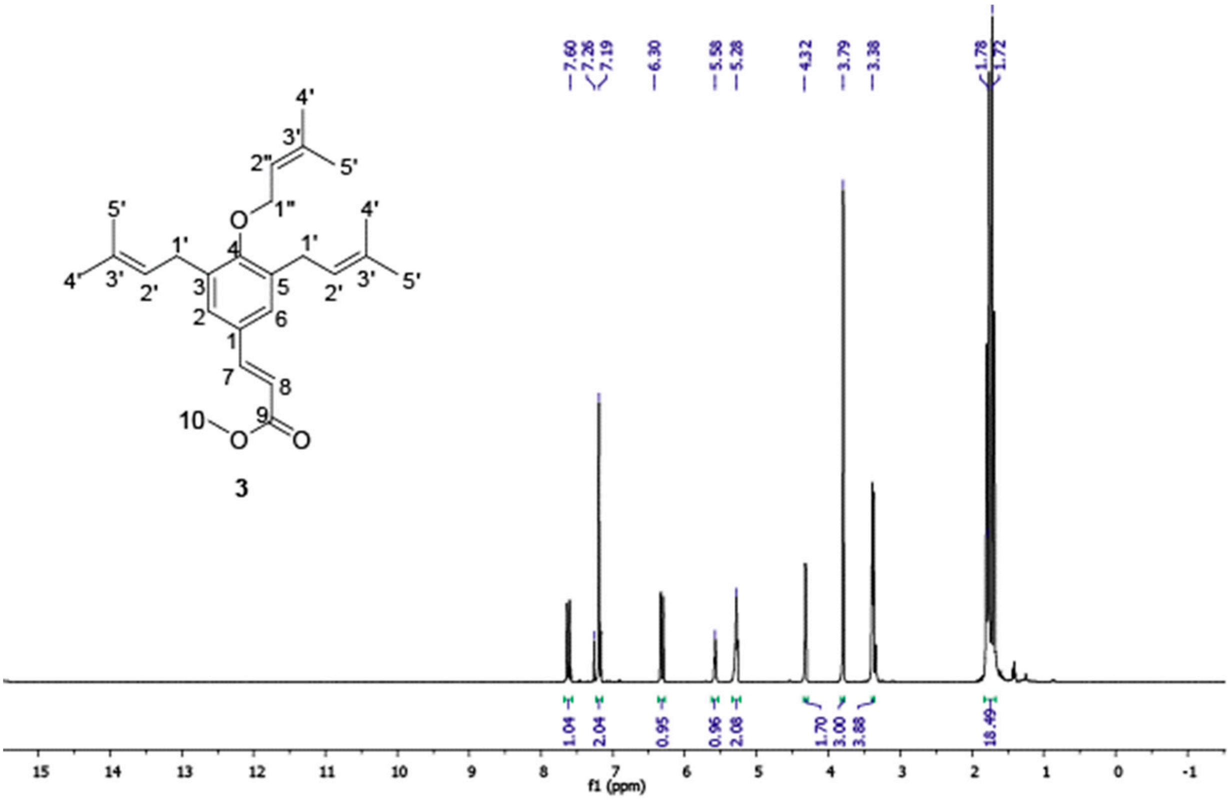

Figure S9. ^{1}H NMR spectrum of compound **3** (400 MHz, $CDCl_3$, TMS).

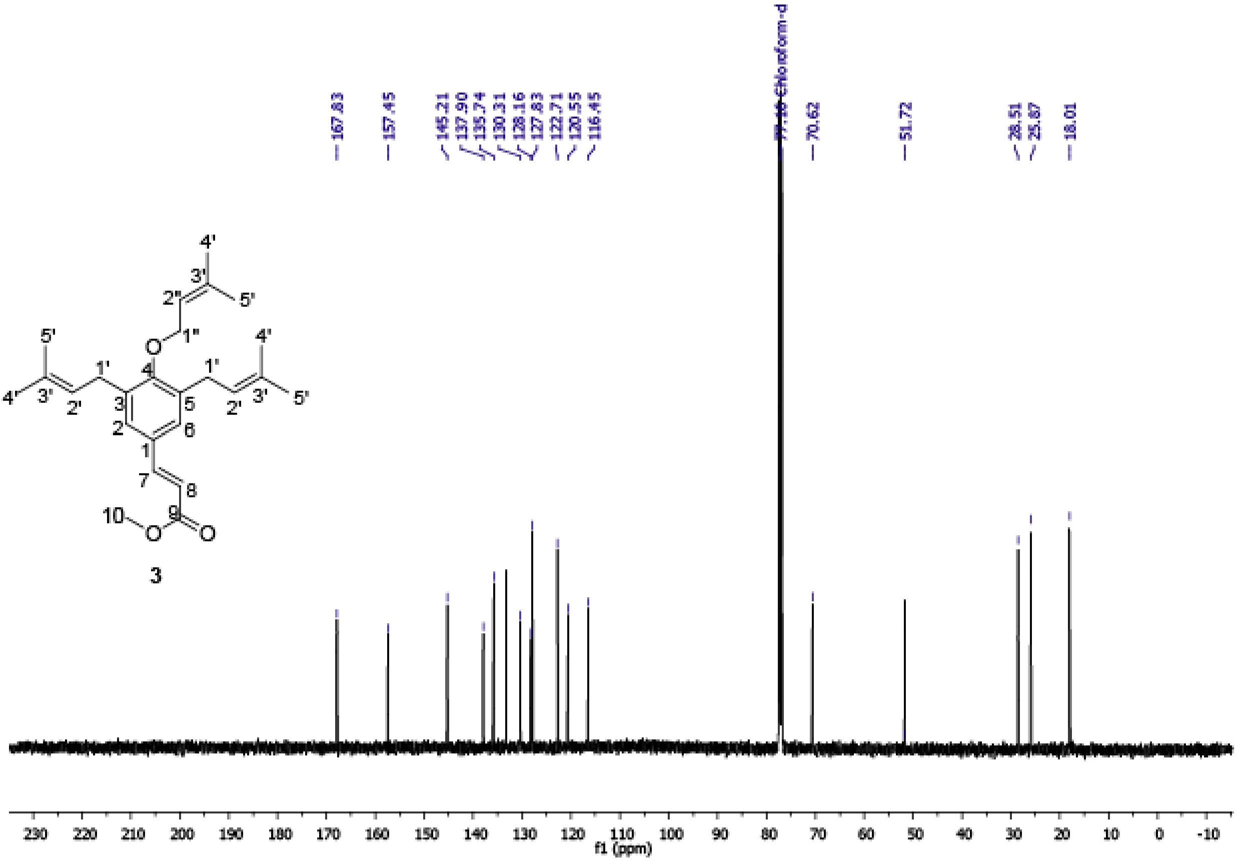

Figure S10. ^{13}C NMR spectrum of compound **3** (400 MHz, $CDCl_3$, TMS).

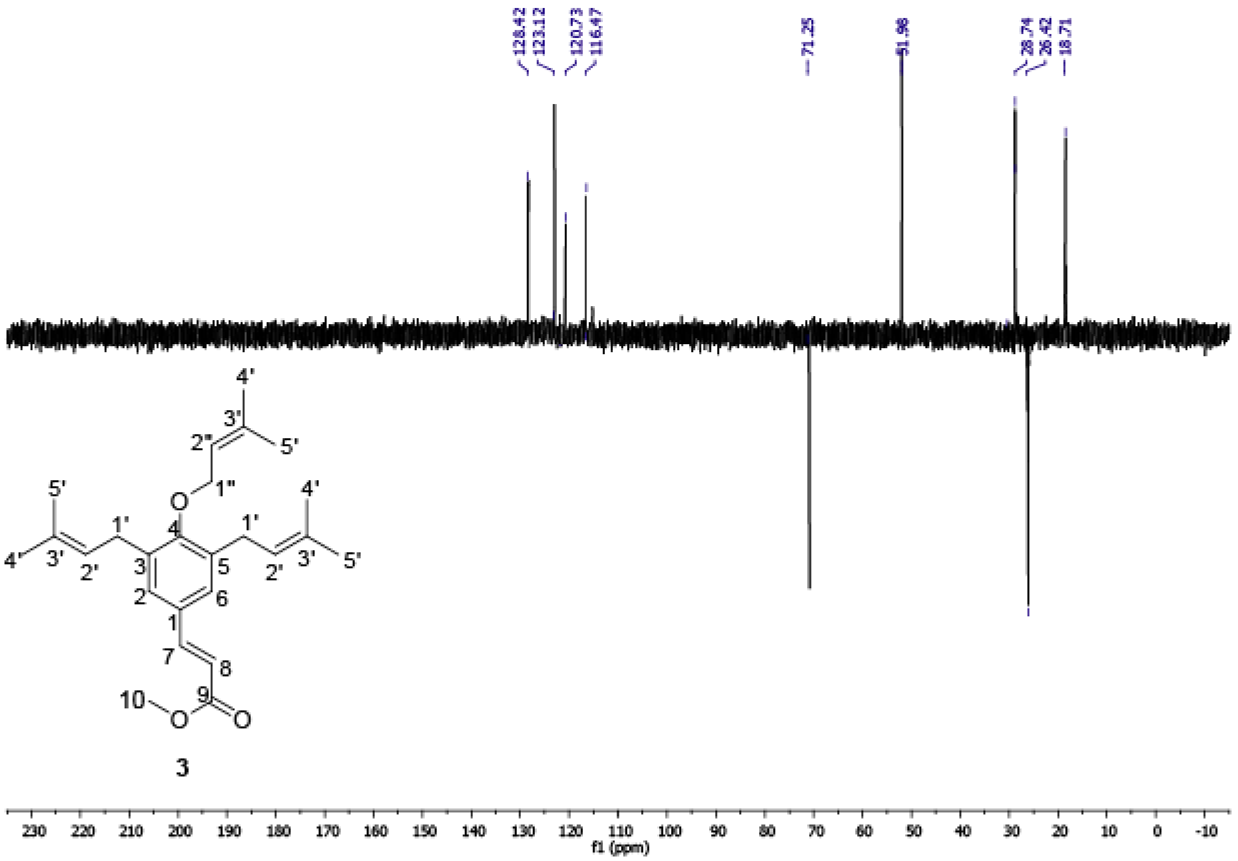

Figure S11. DEPT 135 spectrum of compound **3** (100 MHz, $CDCl_3$, TMS)

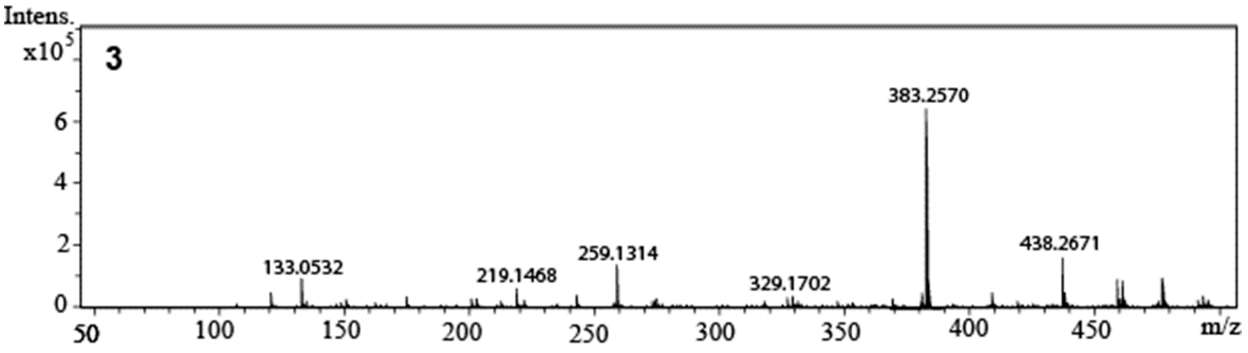

Figure S12. ESI(+)-MS spectrum of compound **3** (Q-TOF).

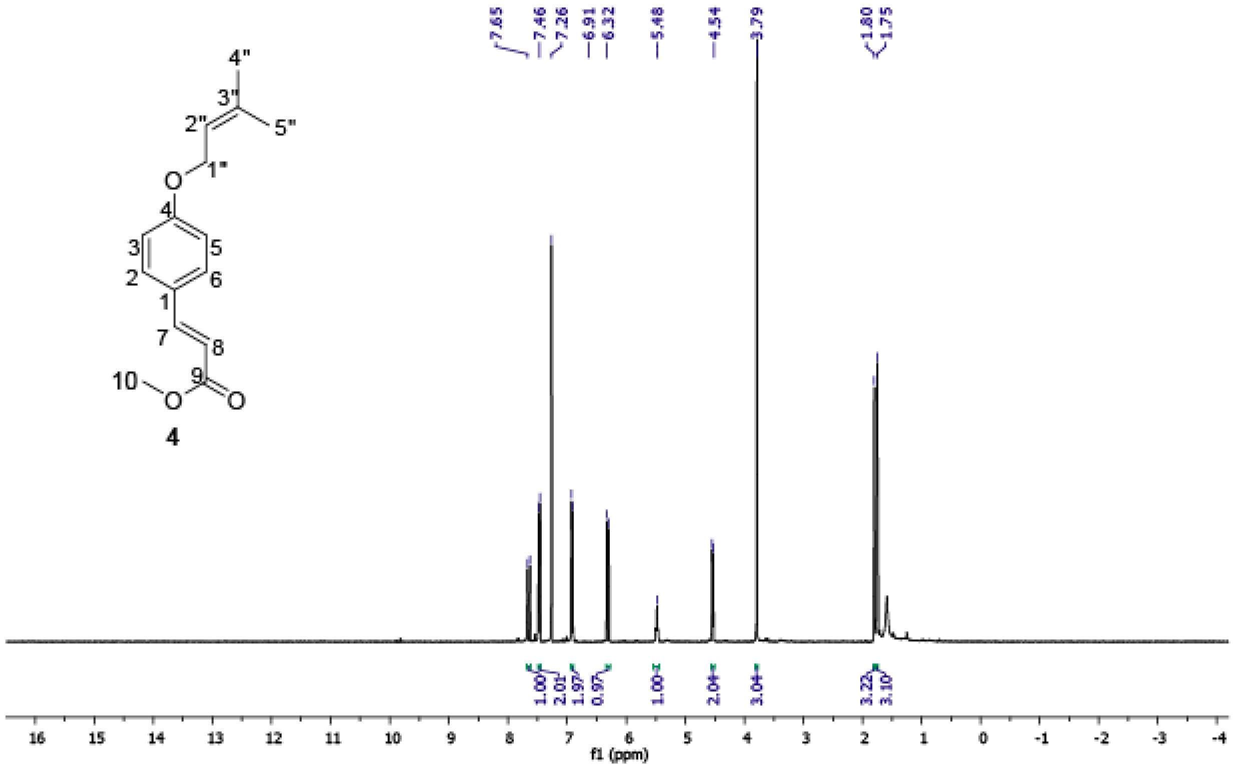

Figure S13. ^{1}H NMR spectrum of compound **4** (400 MHz, $CDCl_3$, TMS).

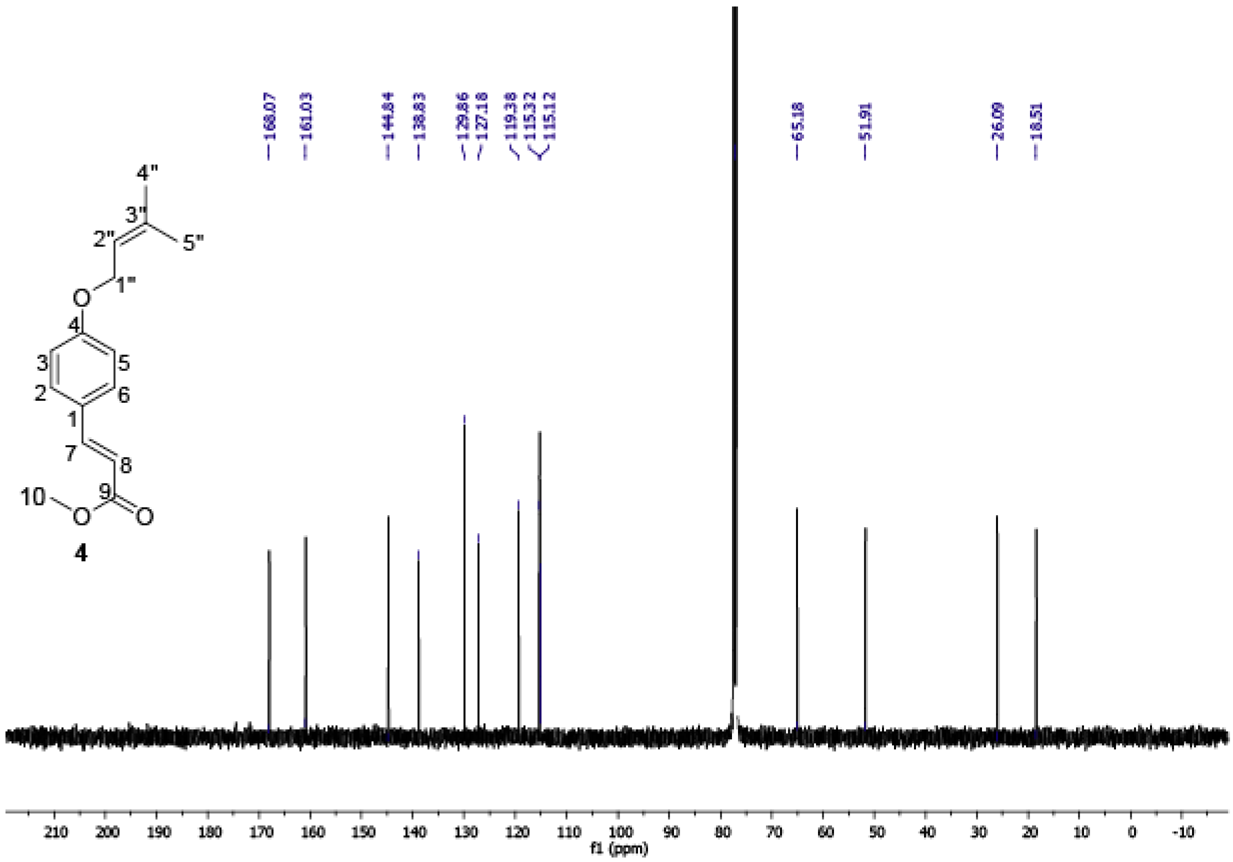

Figure S14. ^{13}C NMR spectrum of compound **4** (400 MHz, $CDCl_3$, TMS).

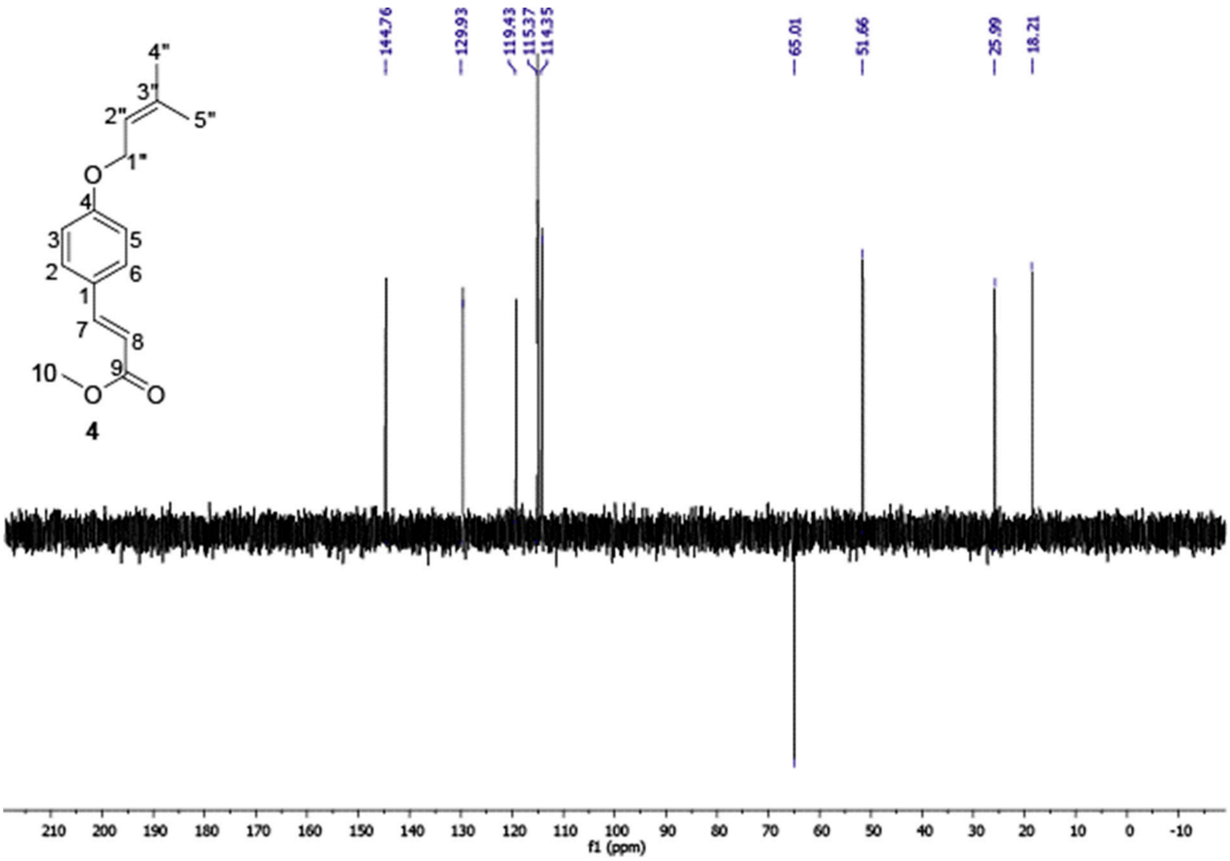

Figure S15. DEPT 135 spectrum of compound **4** (100 MHz, $CDCl_3$, TMS).

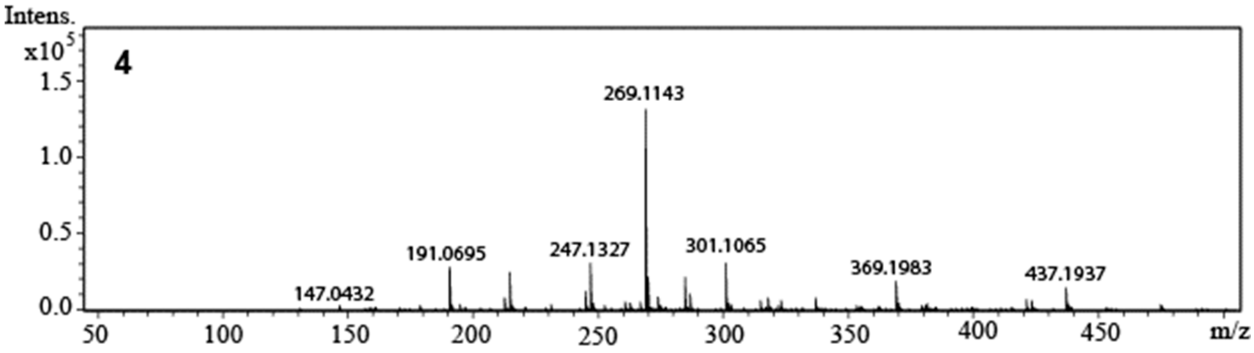

Figure S16. ESI(+)-MS spectrum of compound **4** (Q-TOF).

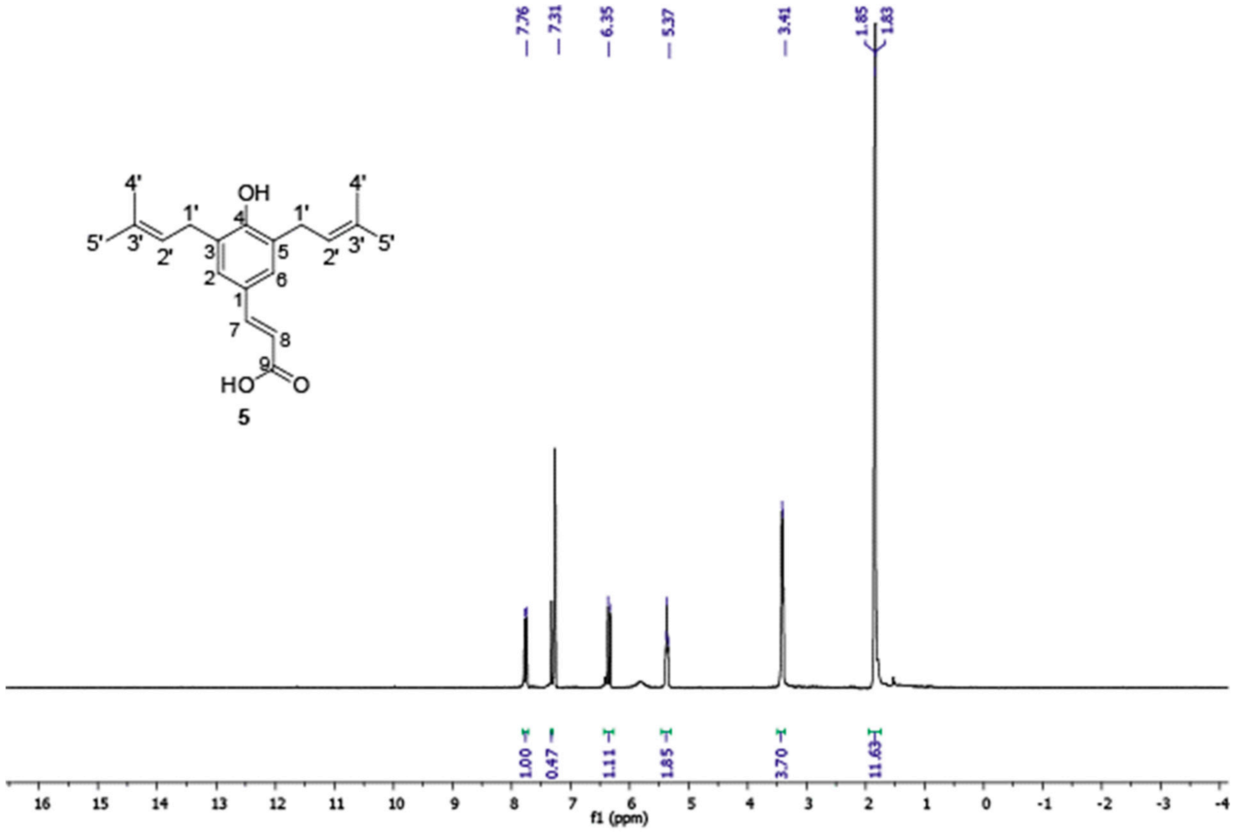

Figure S17. ^{1}H NMR spectrum of compound **5** (400 MHz, $CDCl_3$, TMS).

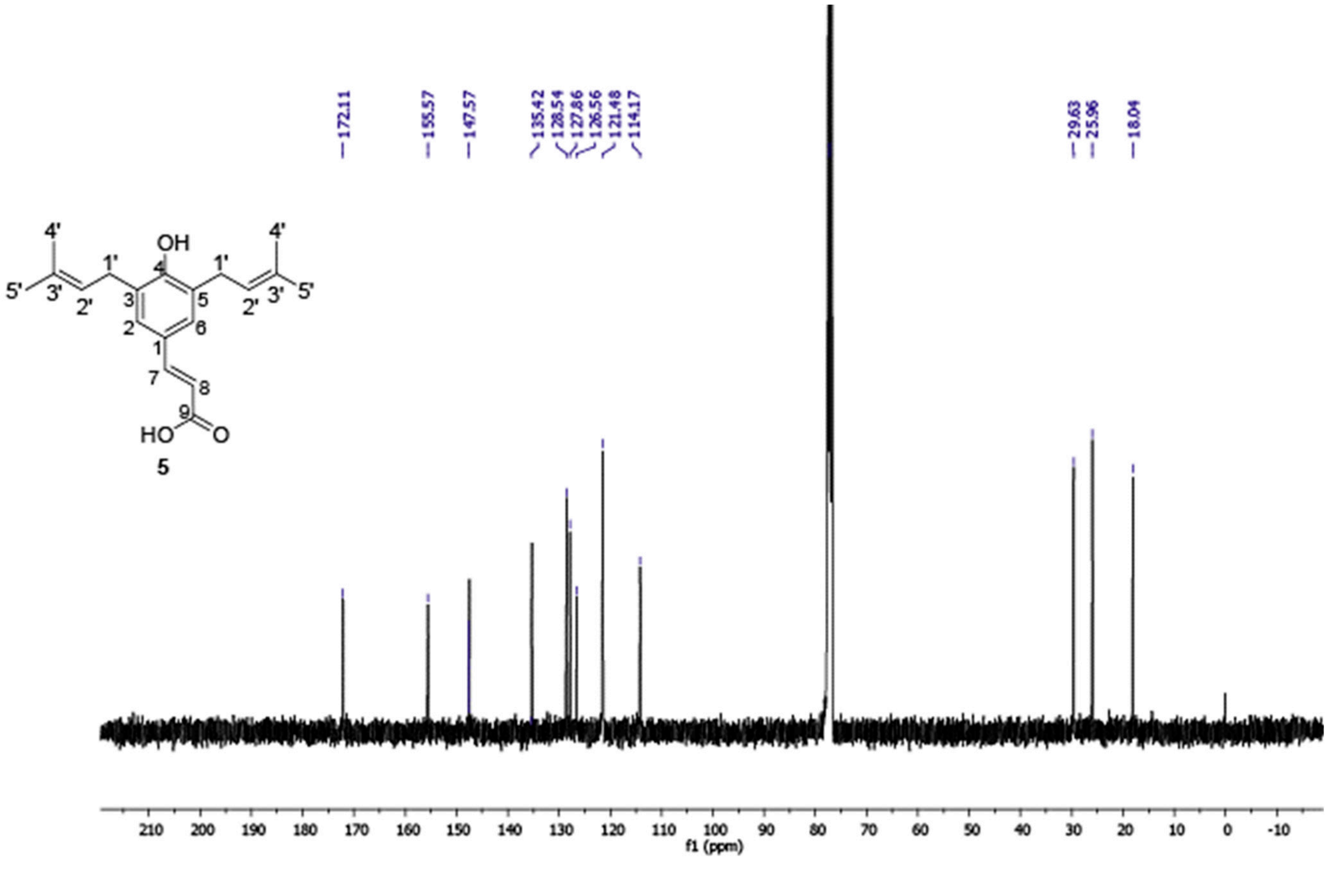

Figure S18. ^{13}C NMR spectrum of compound **5** (400 MHz, $CDCl_3$, TMS).

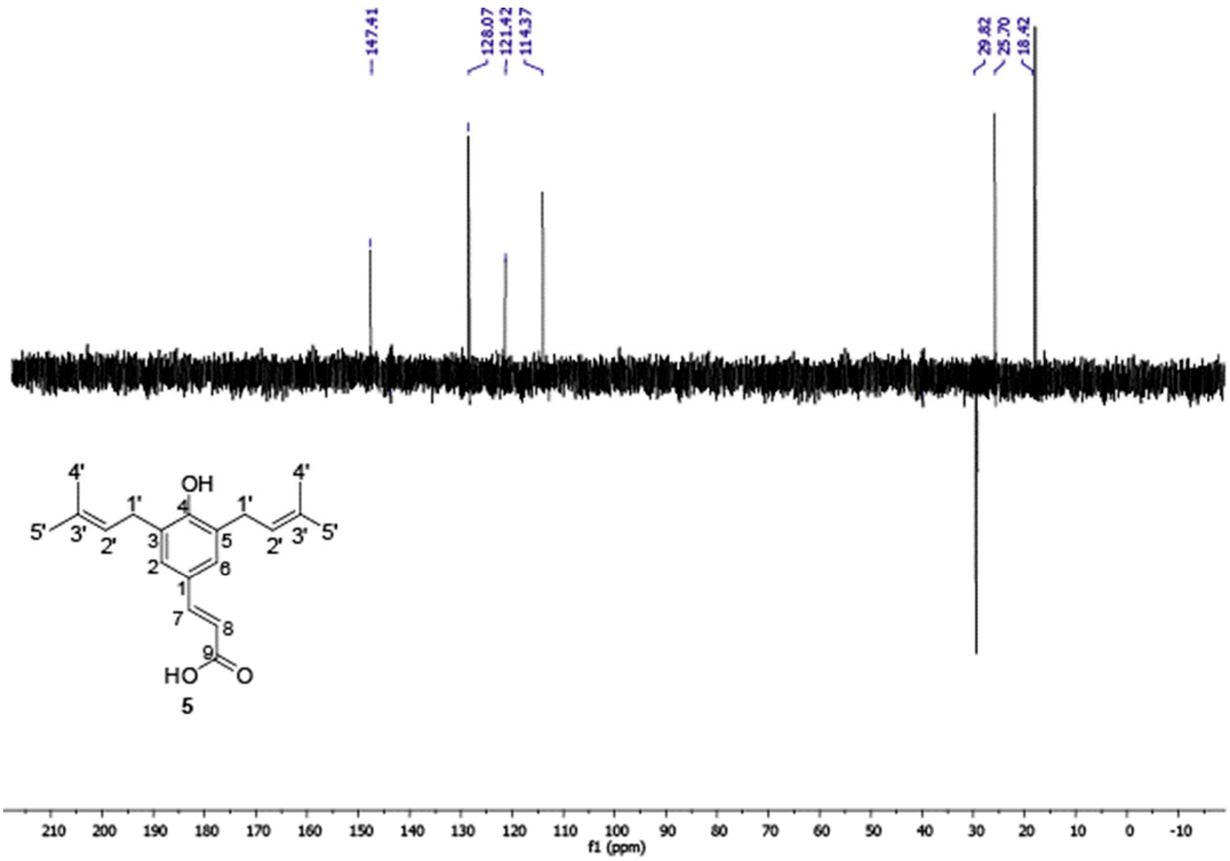

Figure S19. DEPT 135 spectrum of compound **5** (100 MHz, $CDCl_3$, TMS)

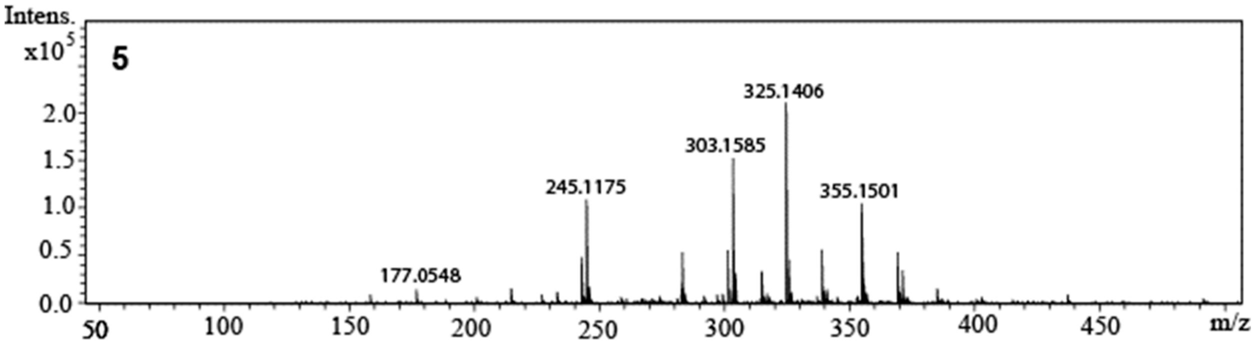

Figure S20. ESI(+)-MS spectrum of compound **5** (Q-TOF).

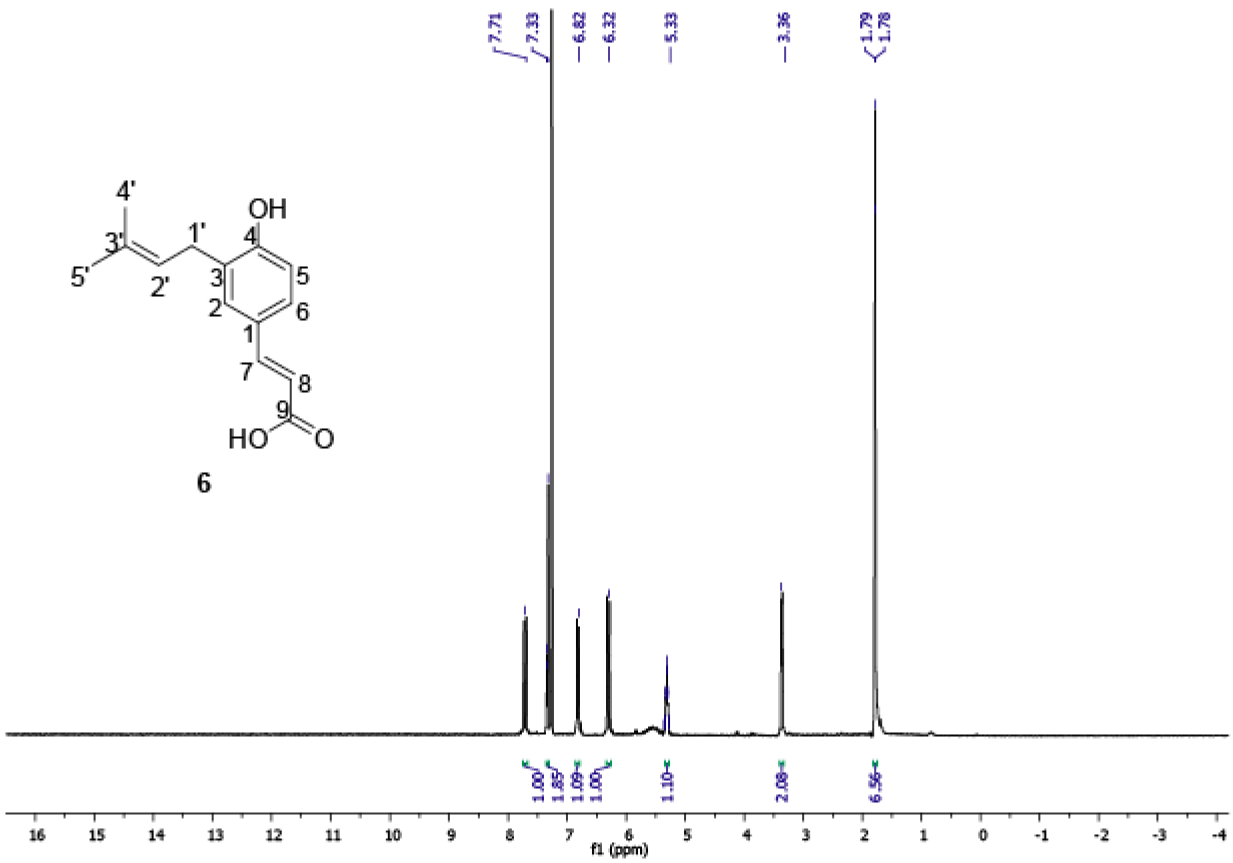

Figure S21. ^{1}H NMR spectrum of compound **6** (400 MHz, $CDCl_3$, TMS).

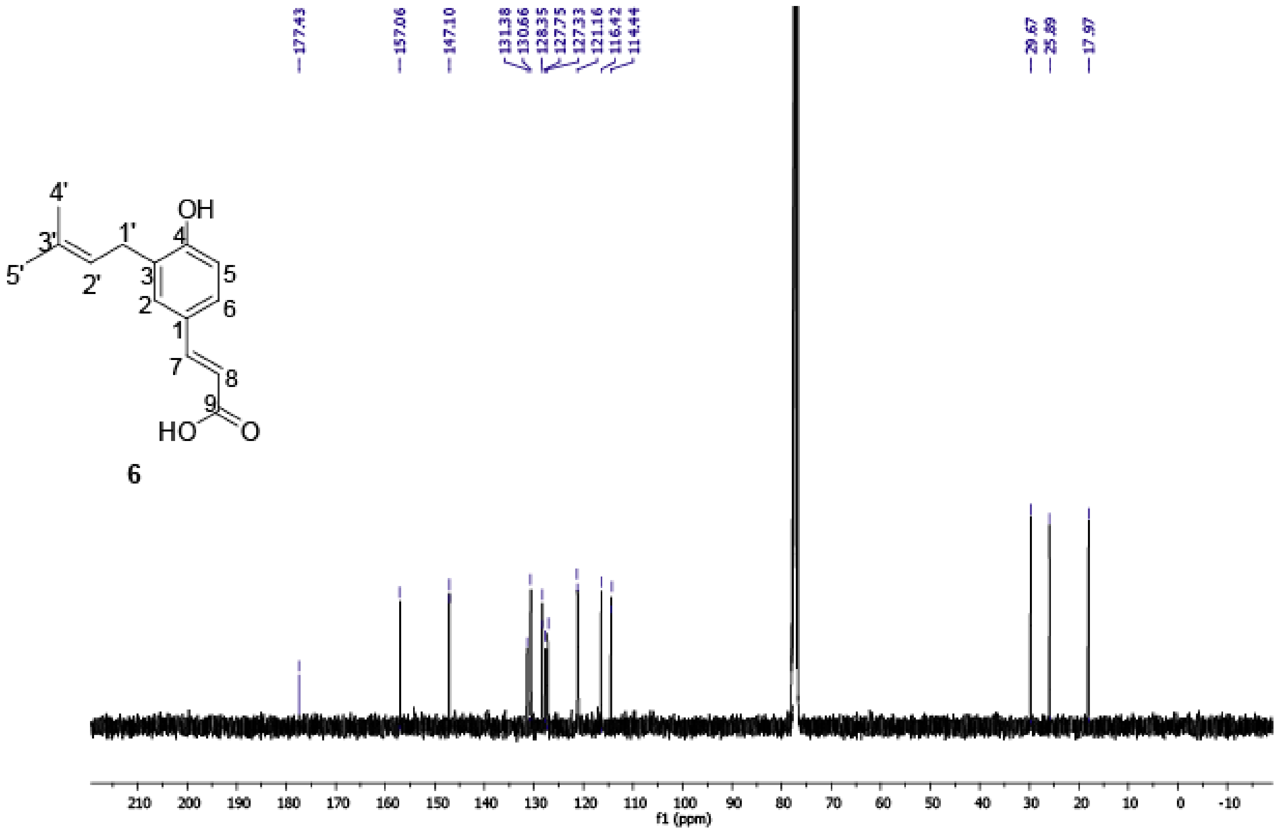

Figure S22. ^{13}C NMR spectrum of compound **6** (400 MHz, $CDCl_3$, TMS).

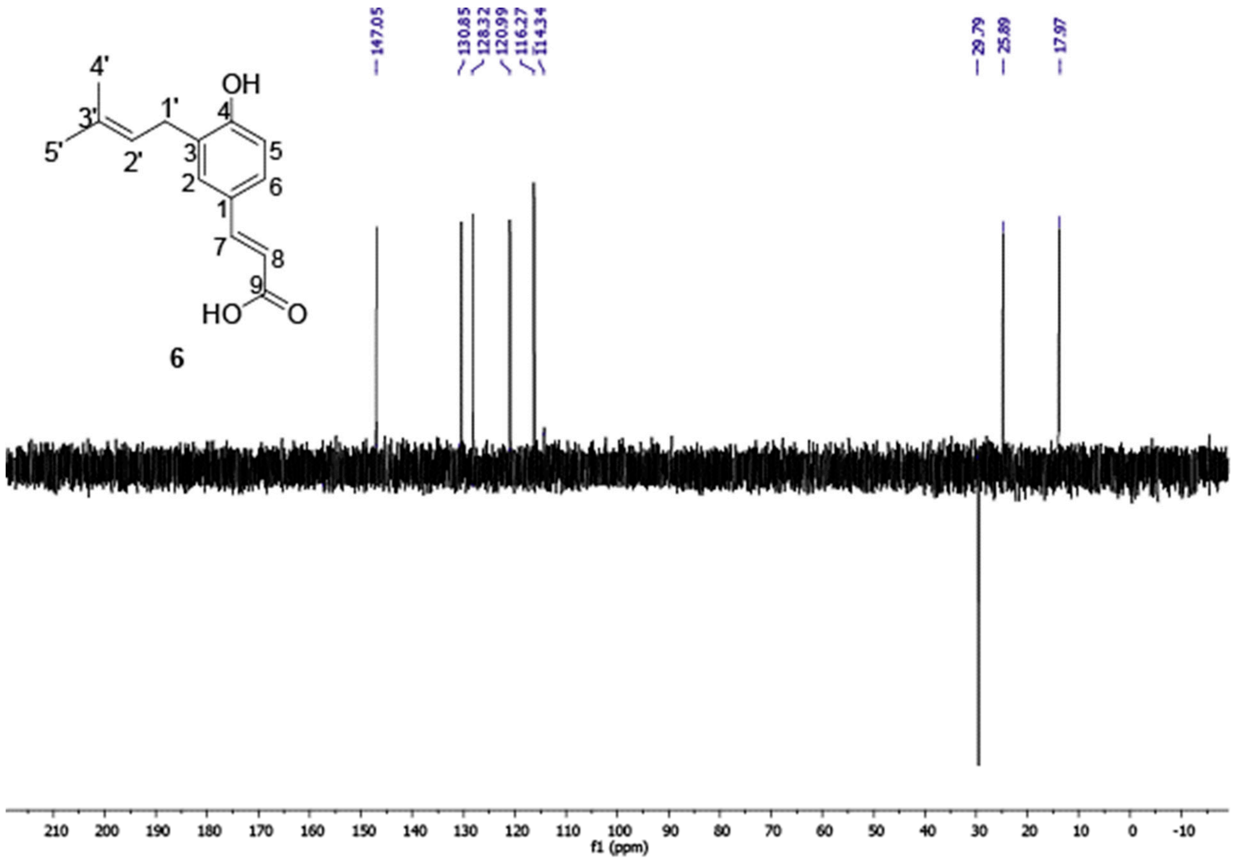

Figure S23. DEPT 135 spectrum of compound **6** (100 MHz, $CDCl_3$, TMS)

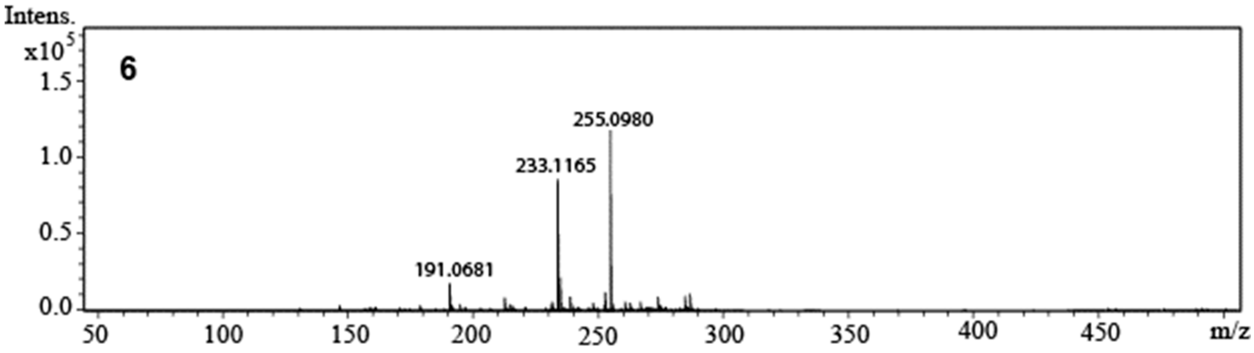

Figure S24. ESI(+)-MS spectrum of compound **6** (Q-TOF).

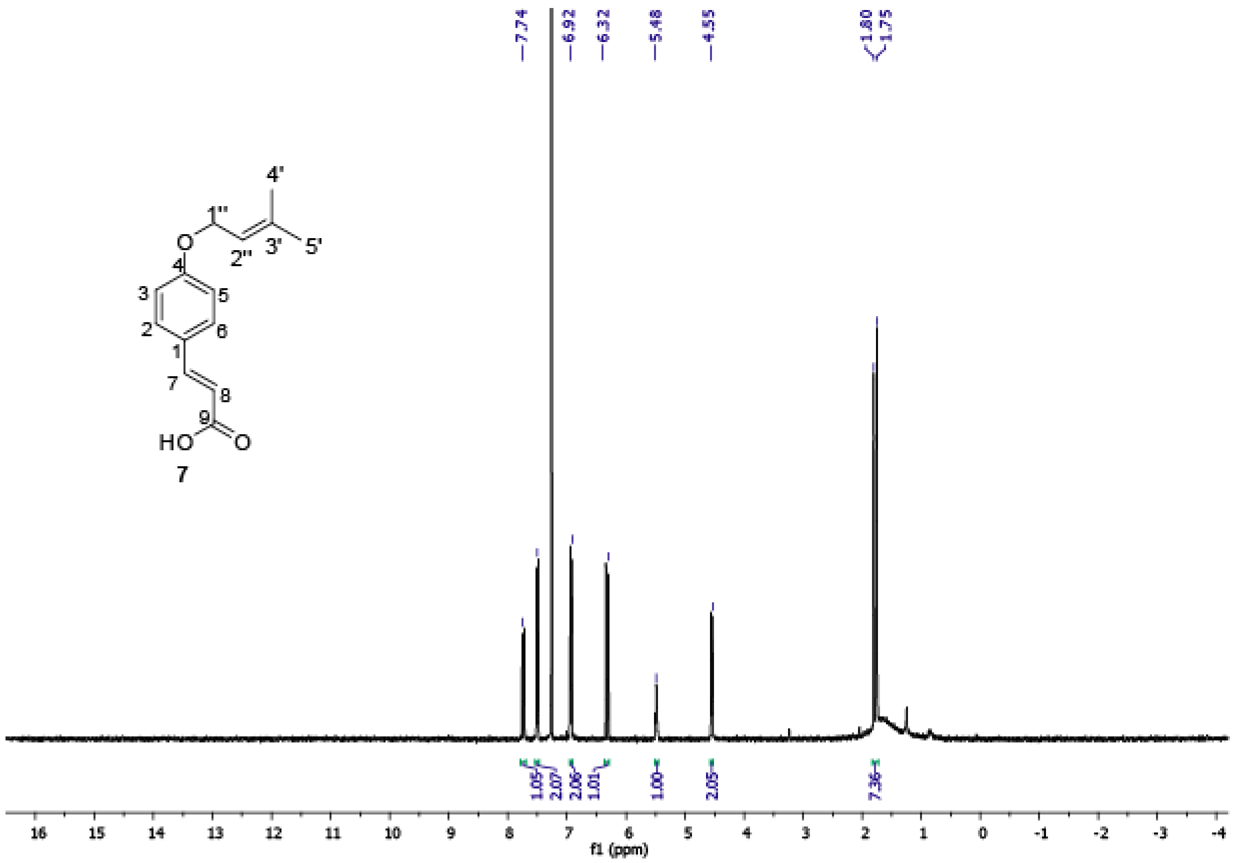

Figure S25. ^{1}H NMR spectrum of compound **7** (400 MHz, $CDCl_3$, TMS).

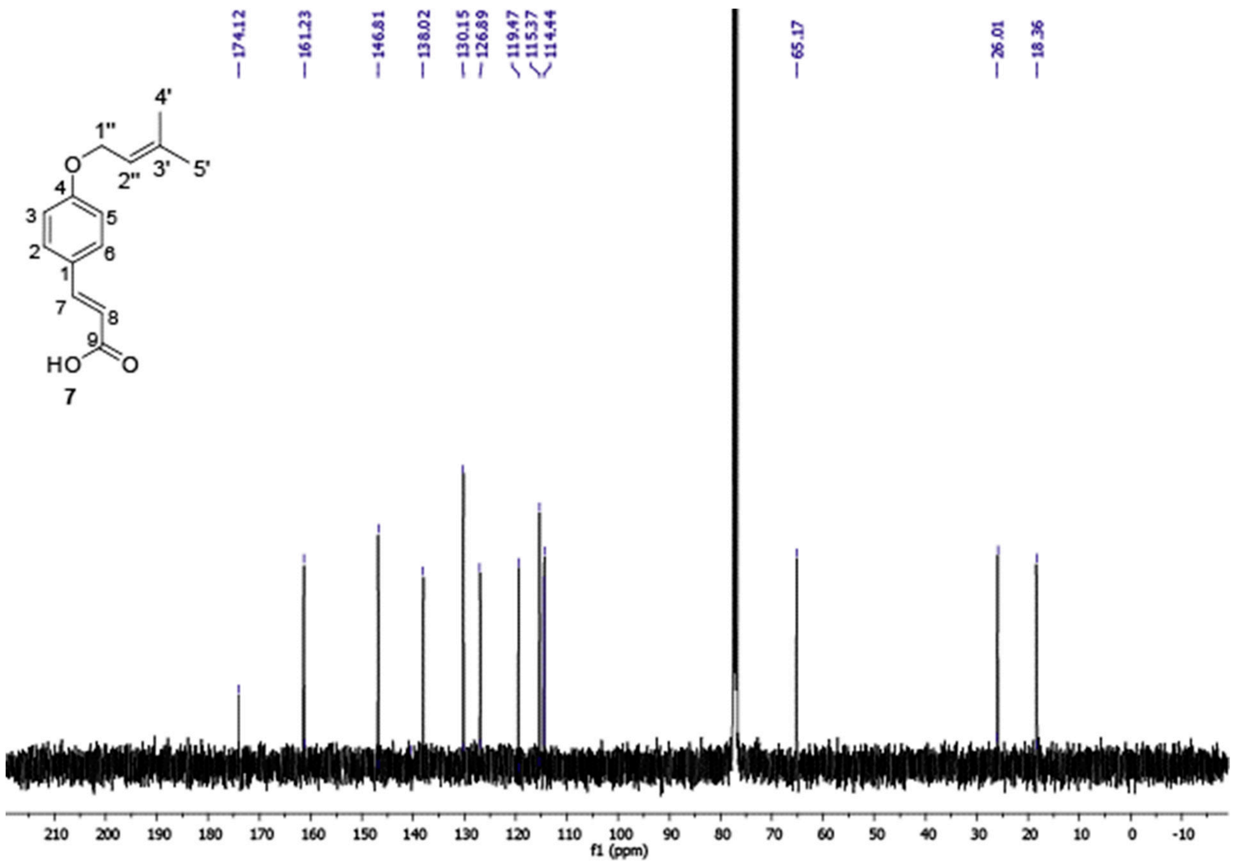

Figure S26. ^{13}C NMR spectrum of compound **7** (400 MHz, $CDCl_3$, TMS).

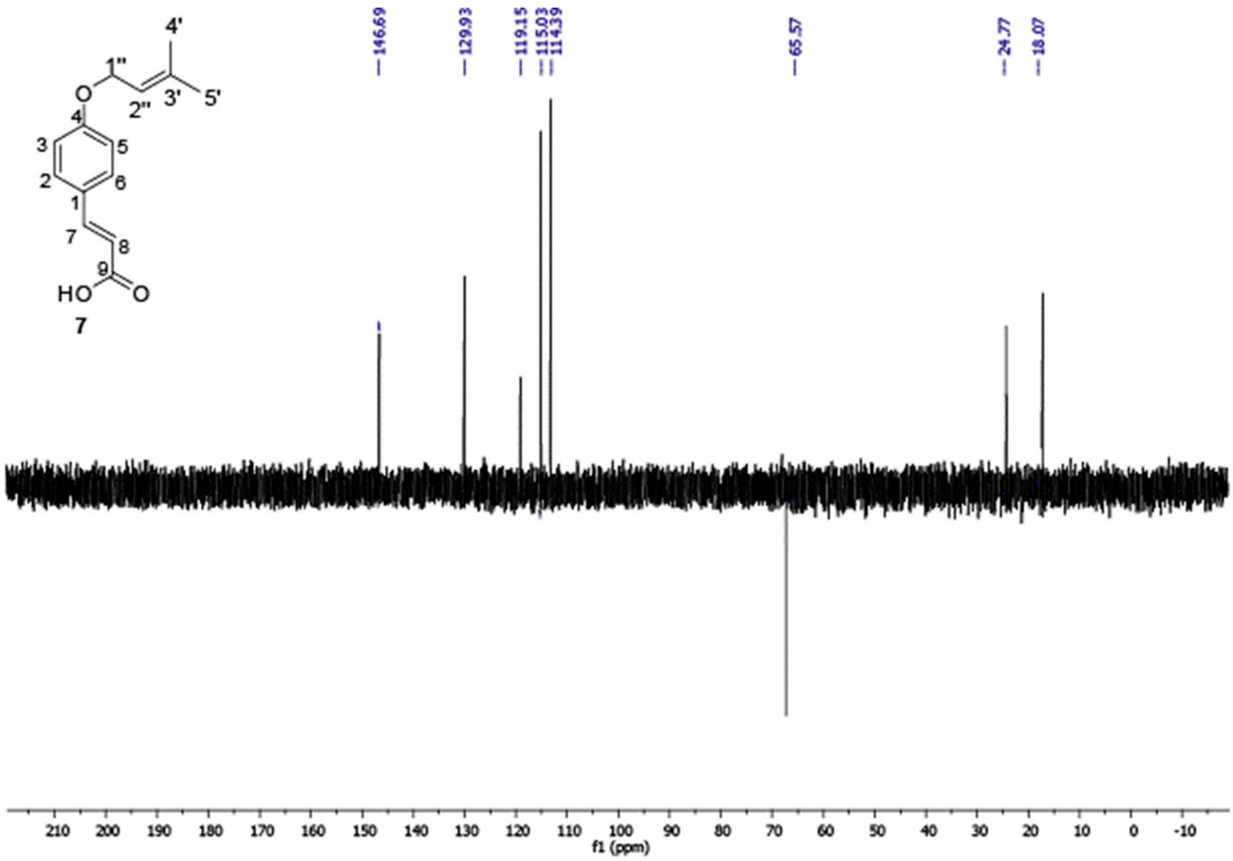

Figure S27. DEPT 135 spectrum of compound **7** (100 MHz, $CDCl_3$, TMS)

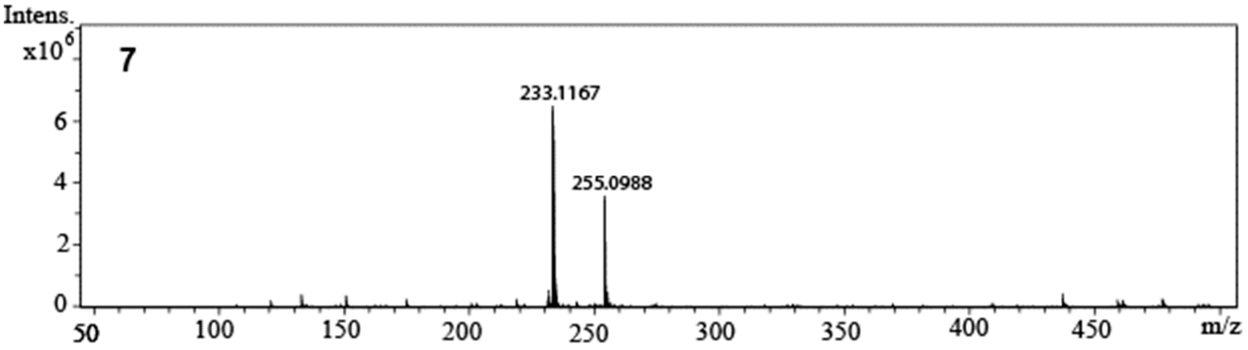

Figure S28. ESI(+)-MS spectrum of compound **7** (Q-TOF).

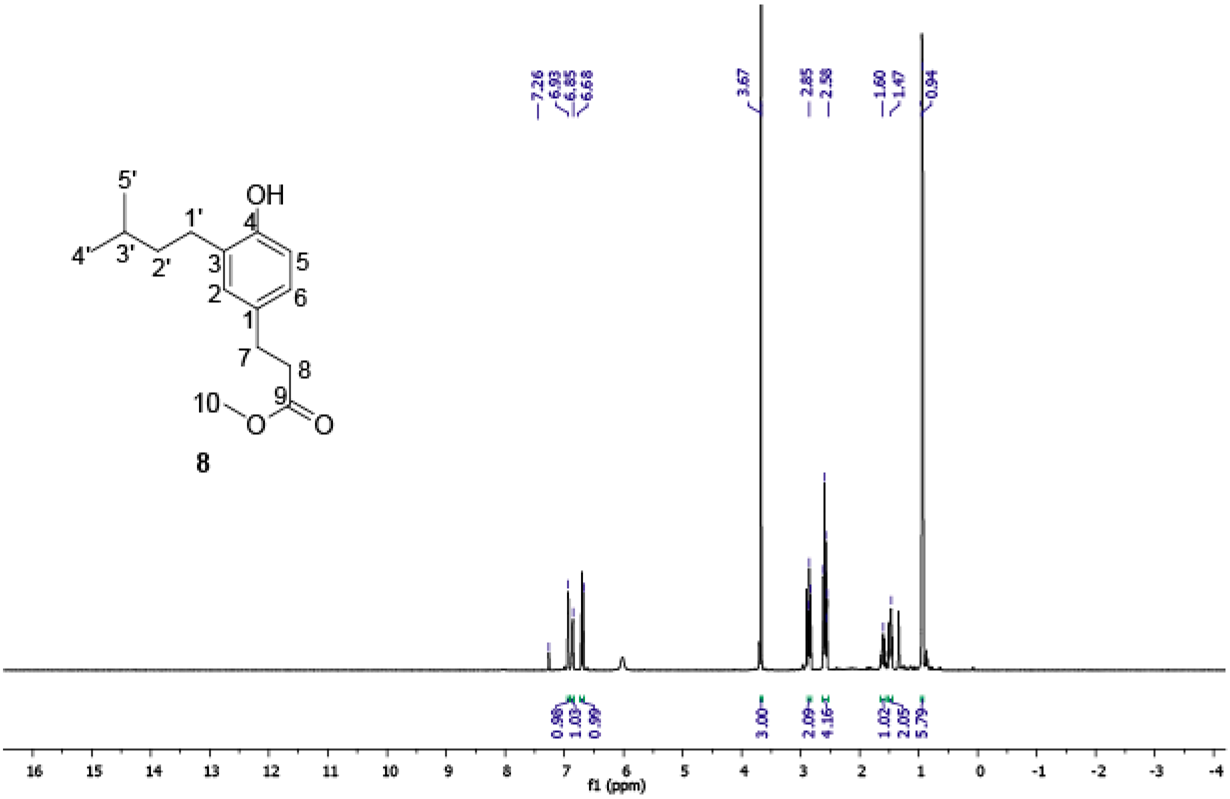

Figure S29. ^{1}H NMR spectrum of compound **8** (400 MHz, $CDCl_3$, TMS).

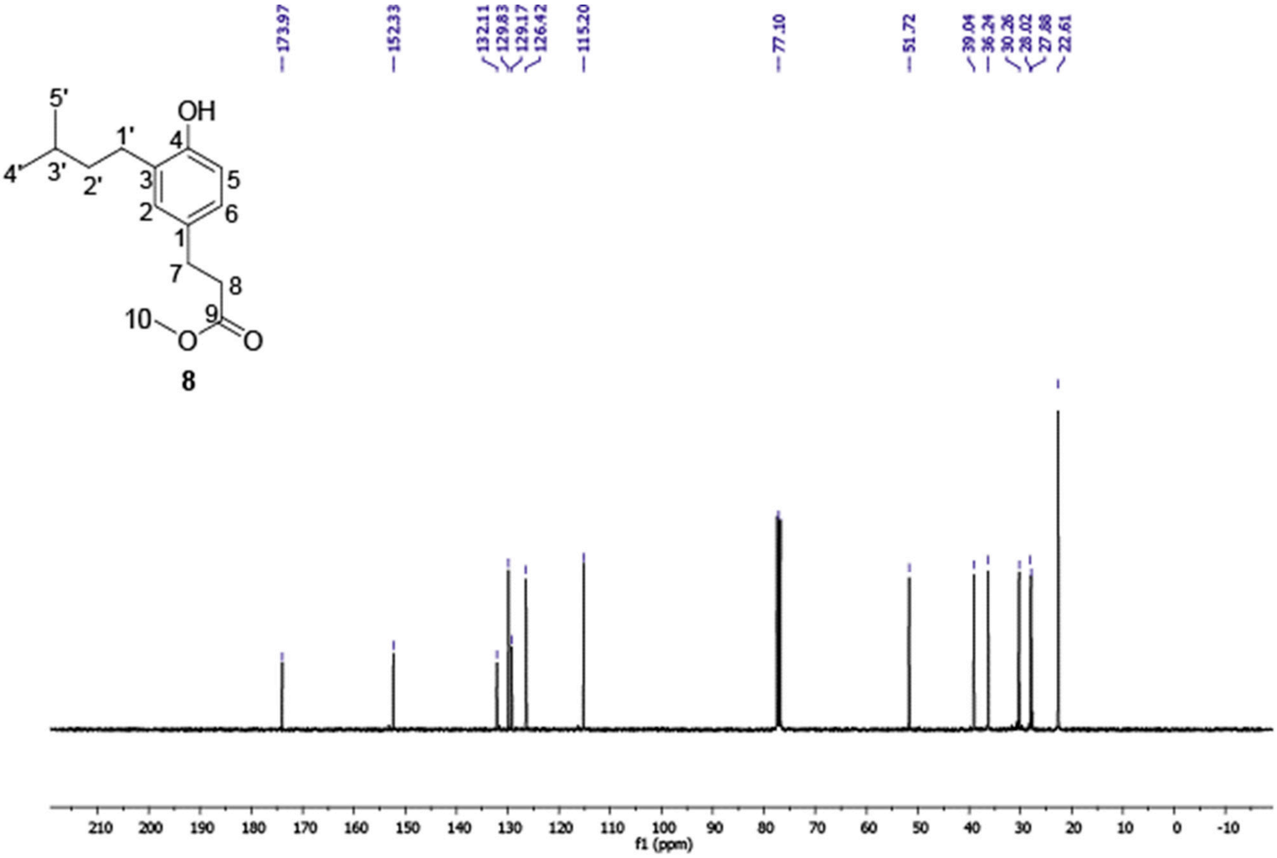

Figure S30. ^{13}C NMR spectrum of compound **8** (400 MHz, $CDCl_3$, TMS).

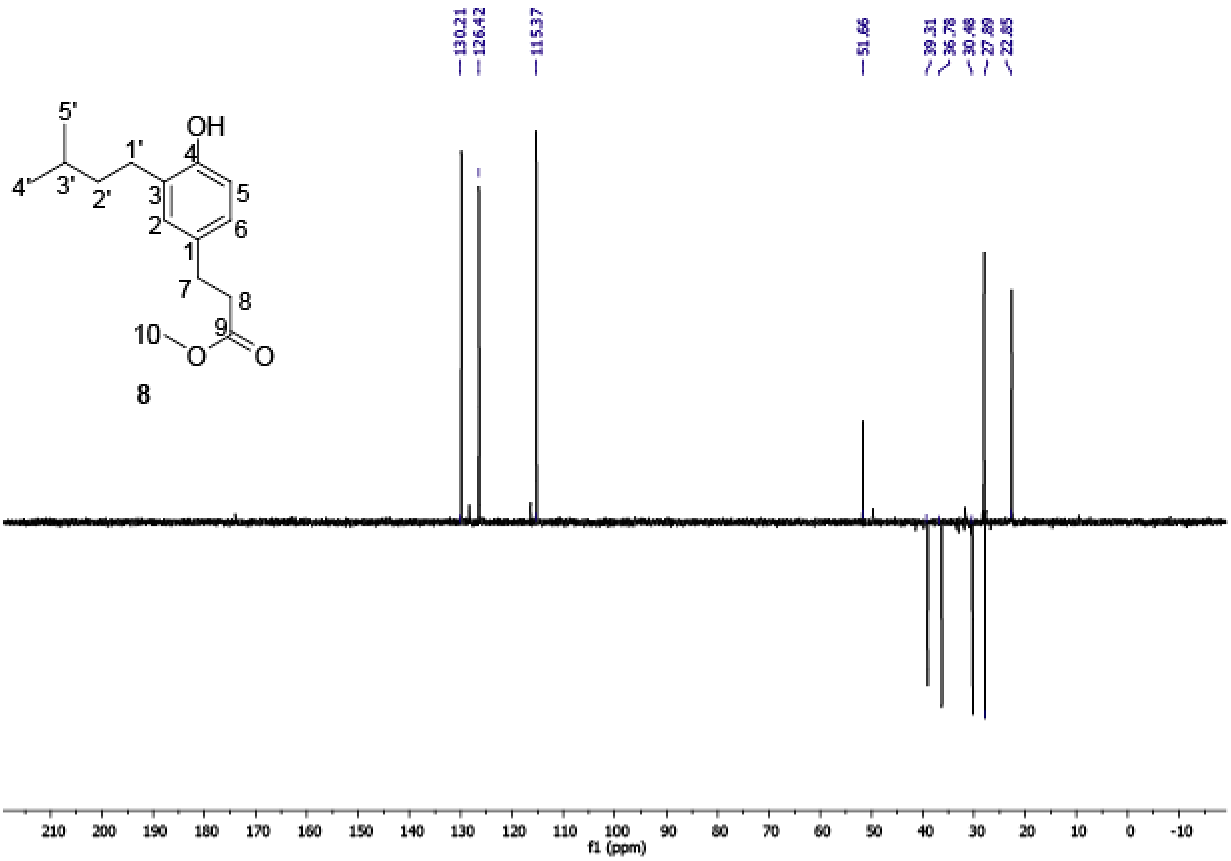

Figure S31. DEPT 135 spectrum of compound **8** (100 MHz, $CDCl_3$, TMS)

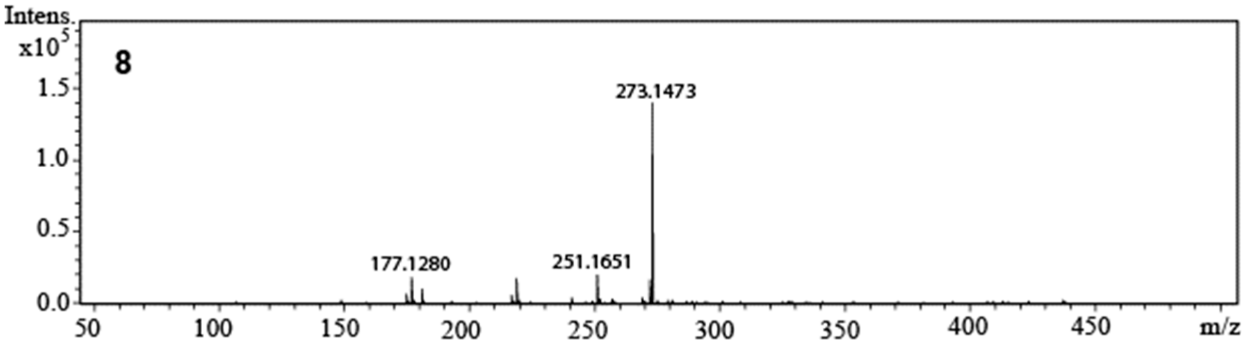

Figure S32. ESI(+)-MS spectrum of compound **8** (Q-TOF).

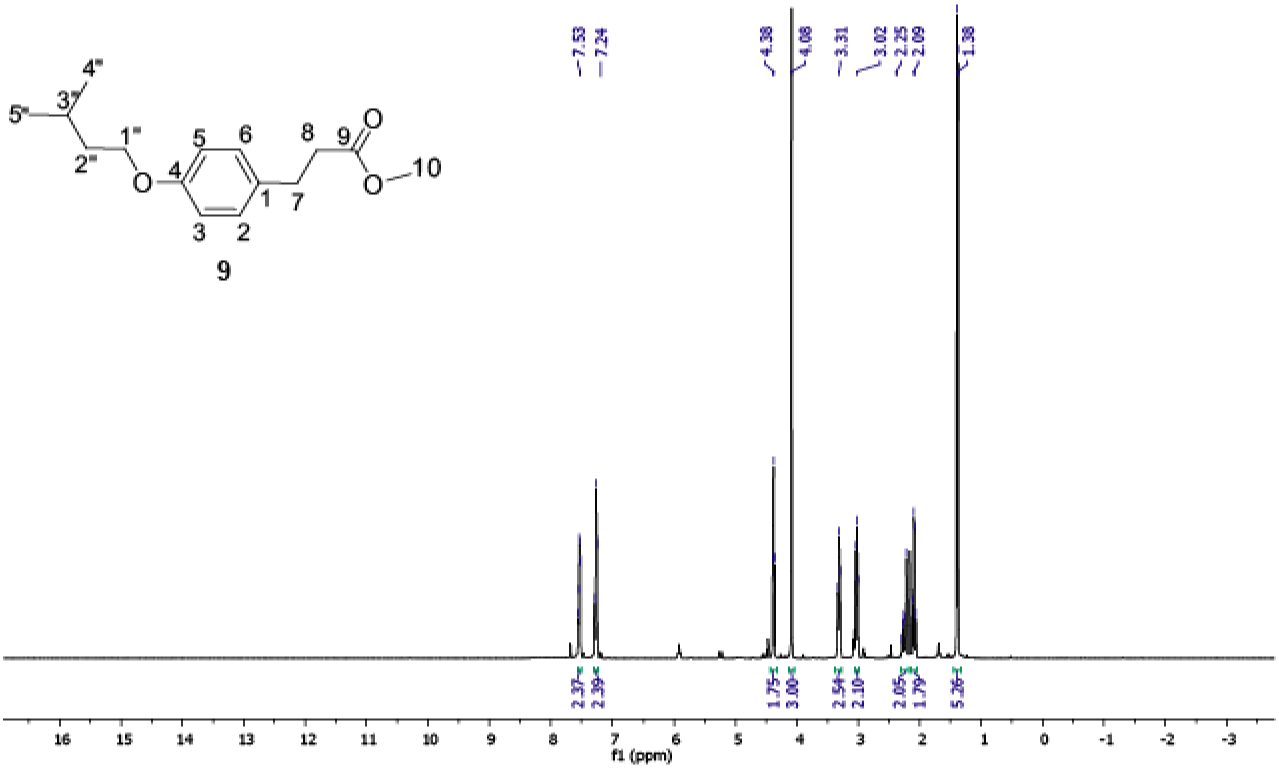

Figure S33. ^{1}H NMR spectrum of compound **9** (400 MHz, $CDCl_3$, TMS).

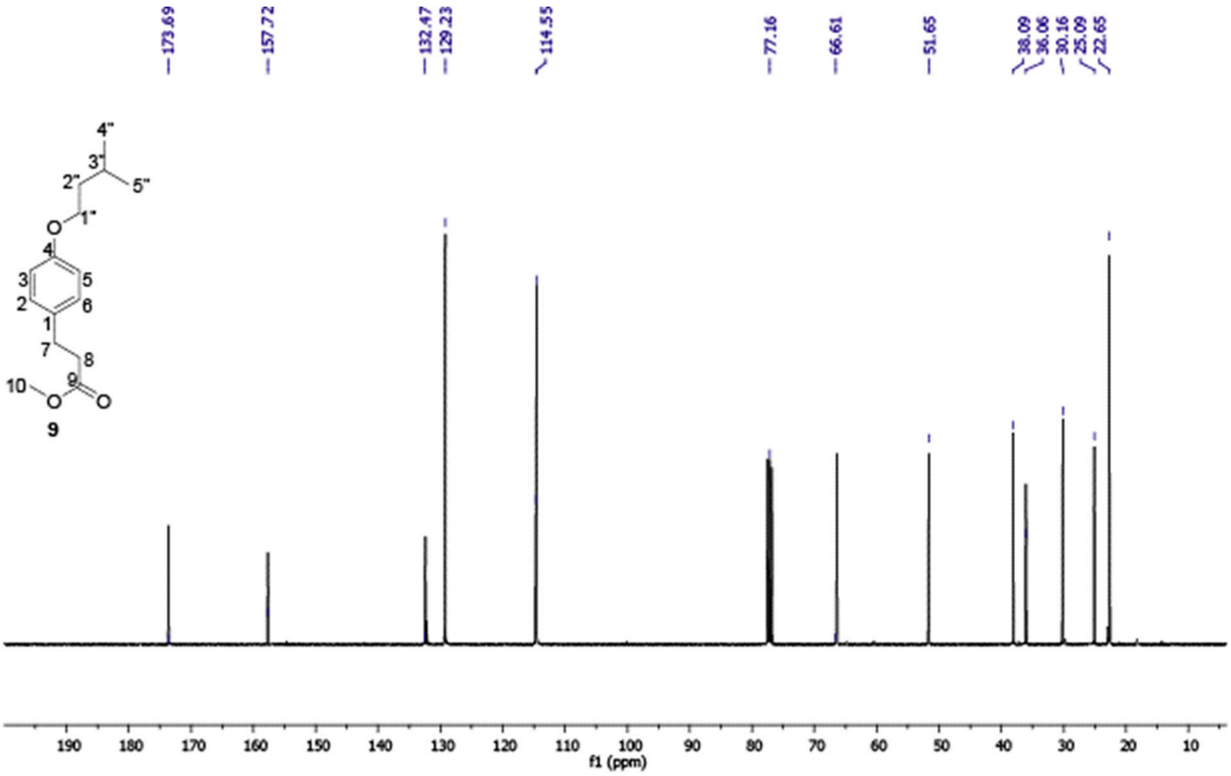

Figure S34. ^{13}C NMR spectrum of compound **9** (400 MHz, $CDCl_3$, TMS).

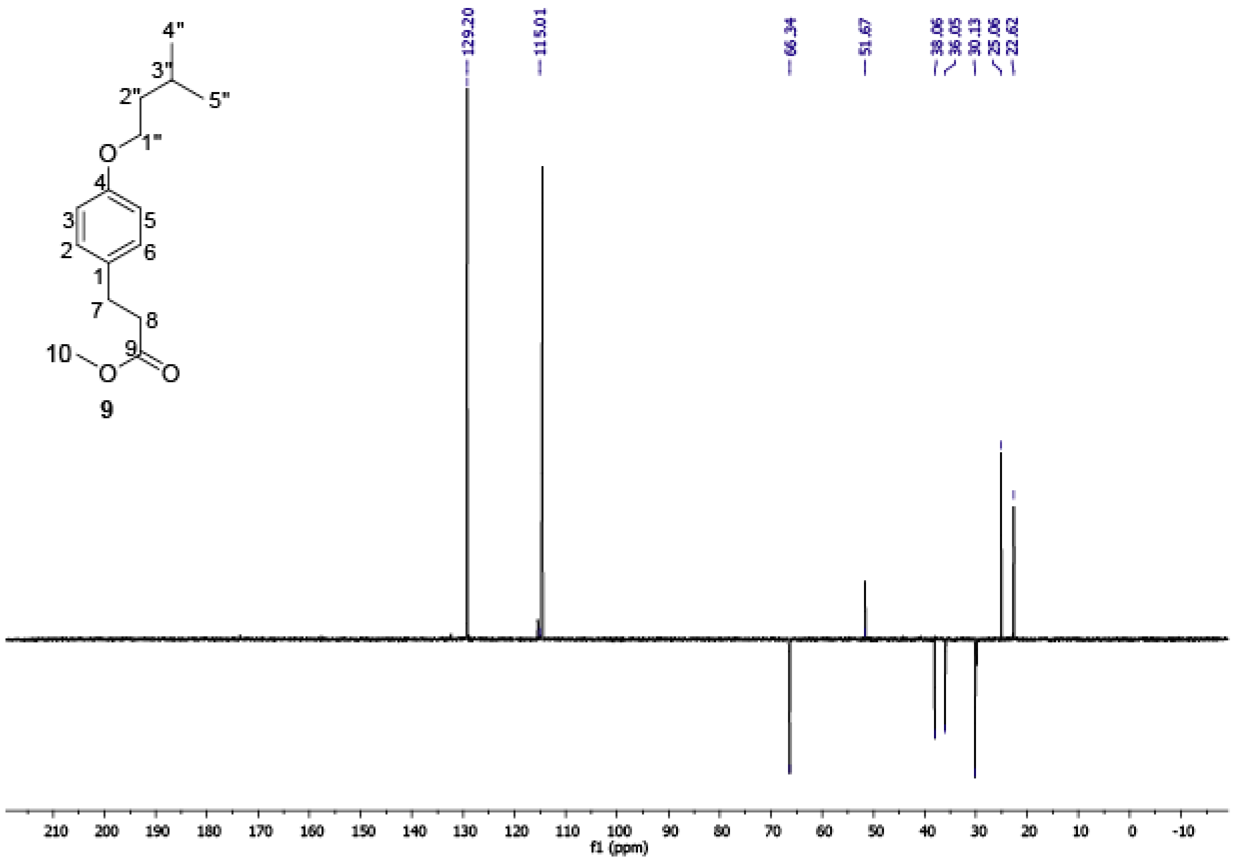

Figure S35. DEPT 135 spectrum of compound **9** (100 MHz, $CDCl_3$, TMS)

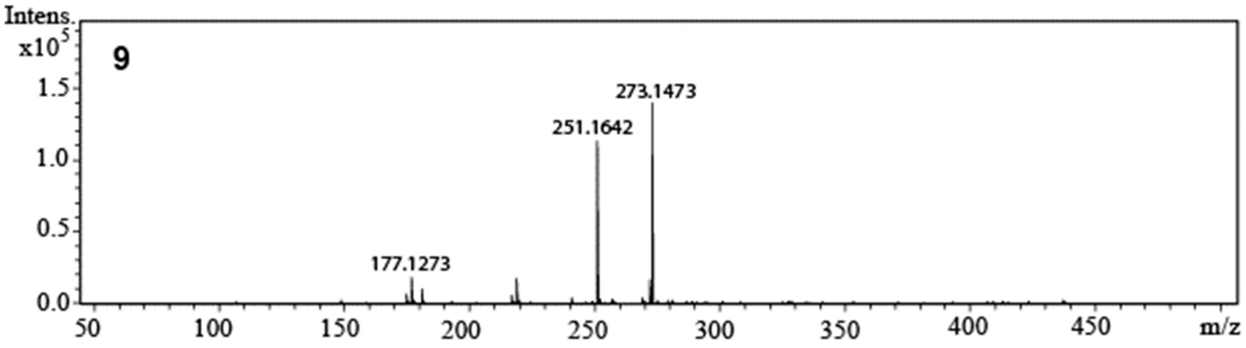

Figure S36. ESI(+)-MS spectrum of compound **9** (Q-TOF).

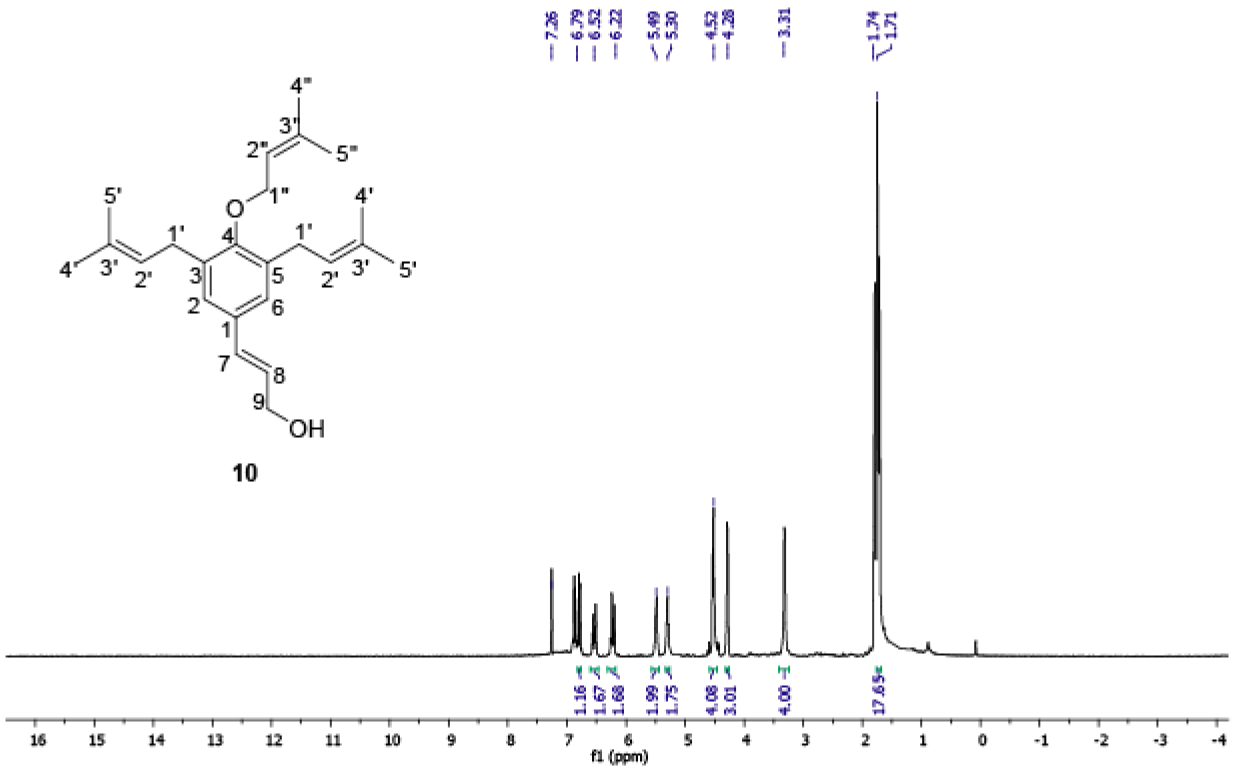

Figure S37. ^{1}H NMR spectrum of compound **10** (400 MHz, $CDCl_3$, TMS).

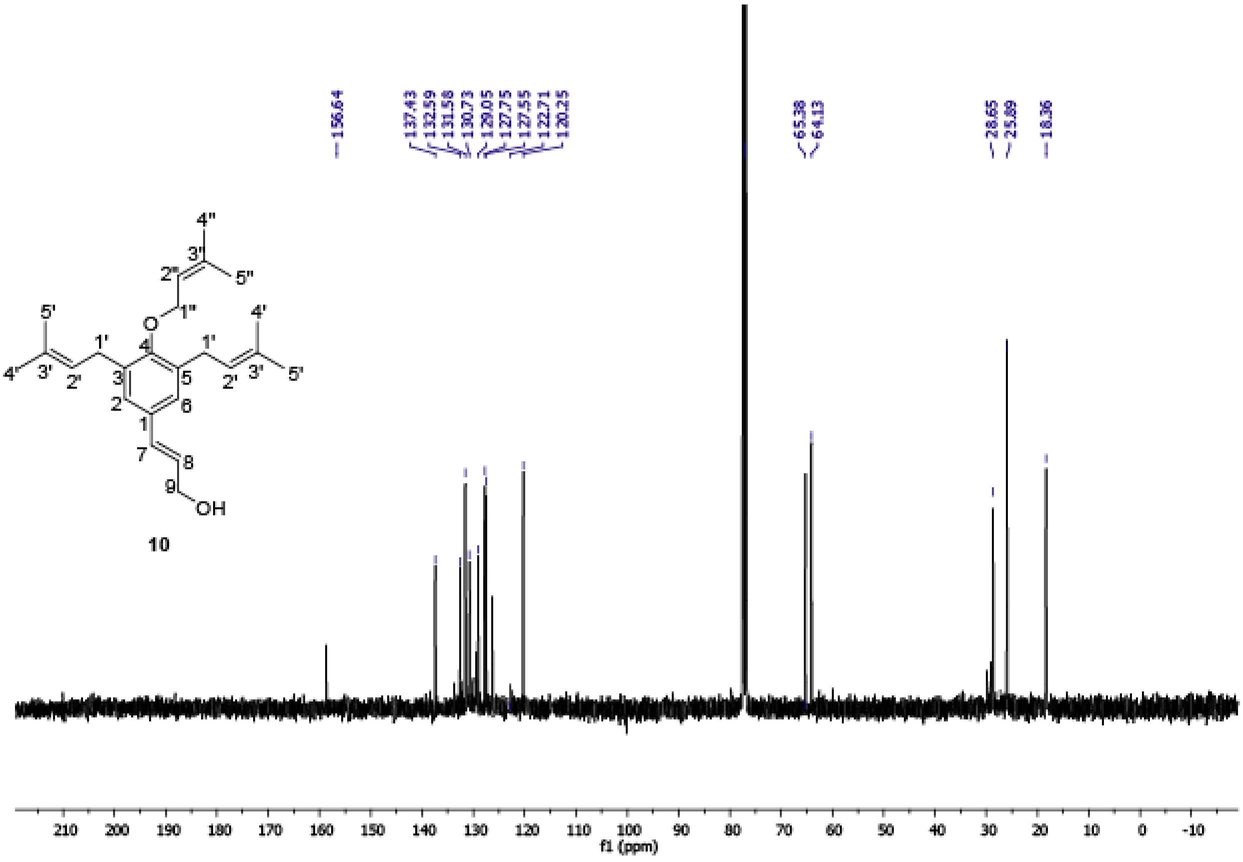

Figure S38. ^{13}C NMR spectrum of compound **10** (400 MHz, $CDCl_3$, TMS).

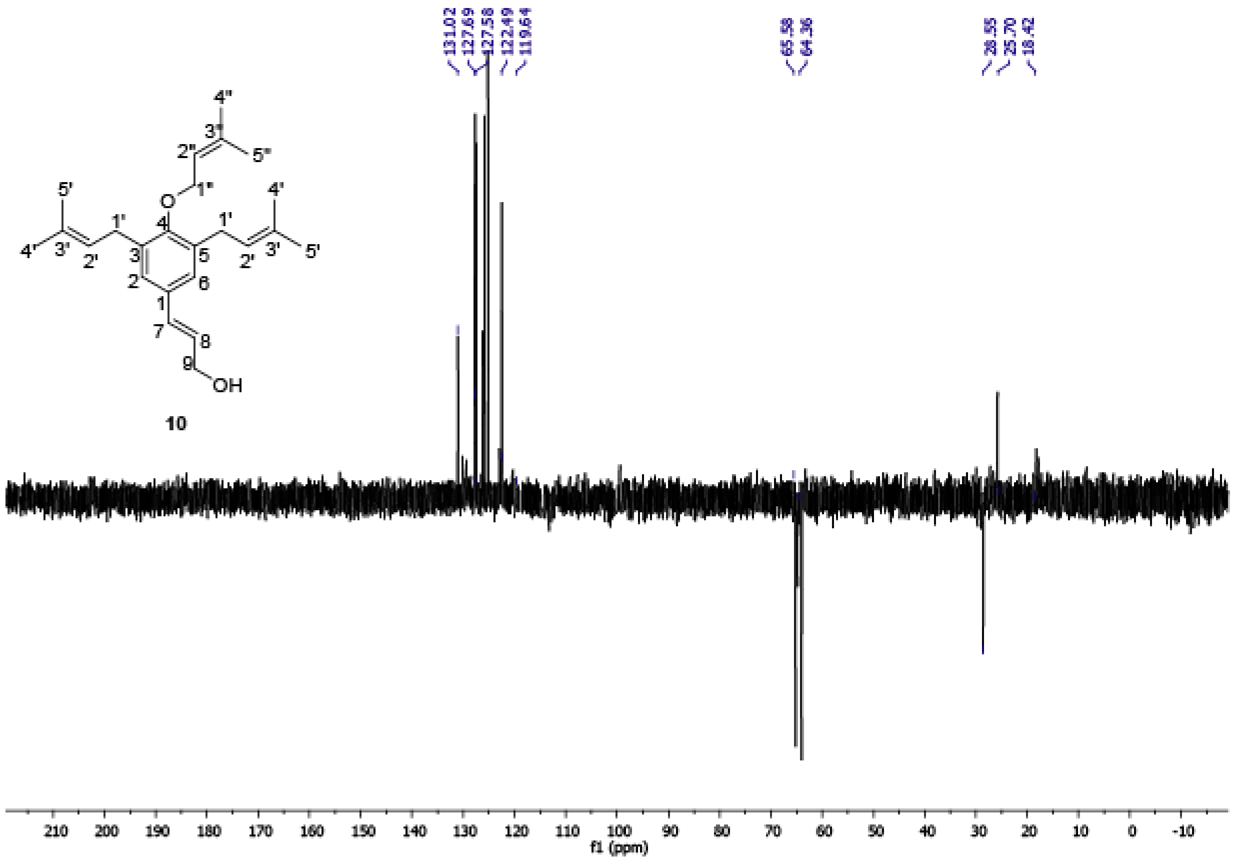

Figure S39. DEPT 135 spectrum of compound **10** (100 MHz, $CDCl_3$, TMS)

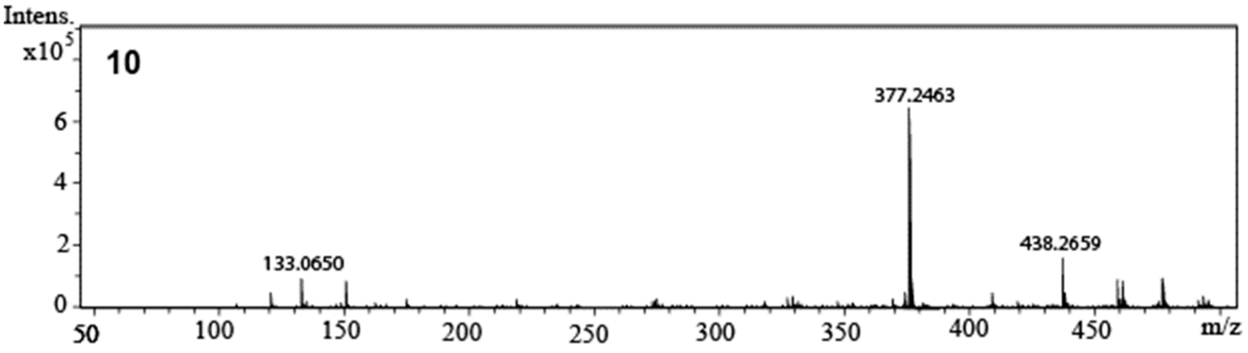

Figure S40. ESI(+)-MS spectrum of compound **10** (Q-TOF).

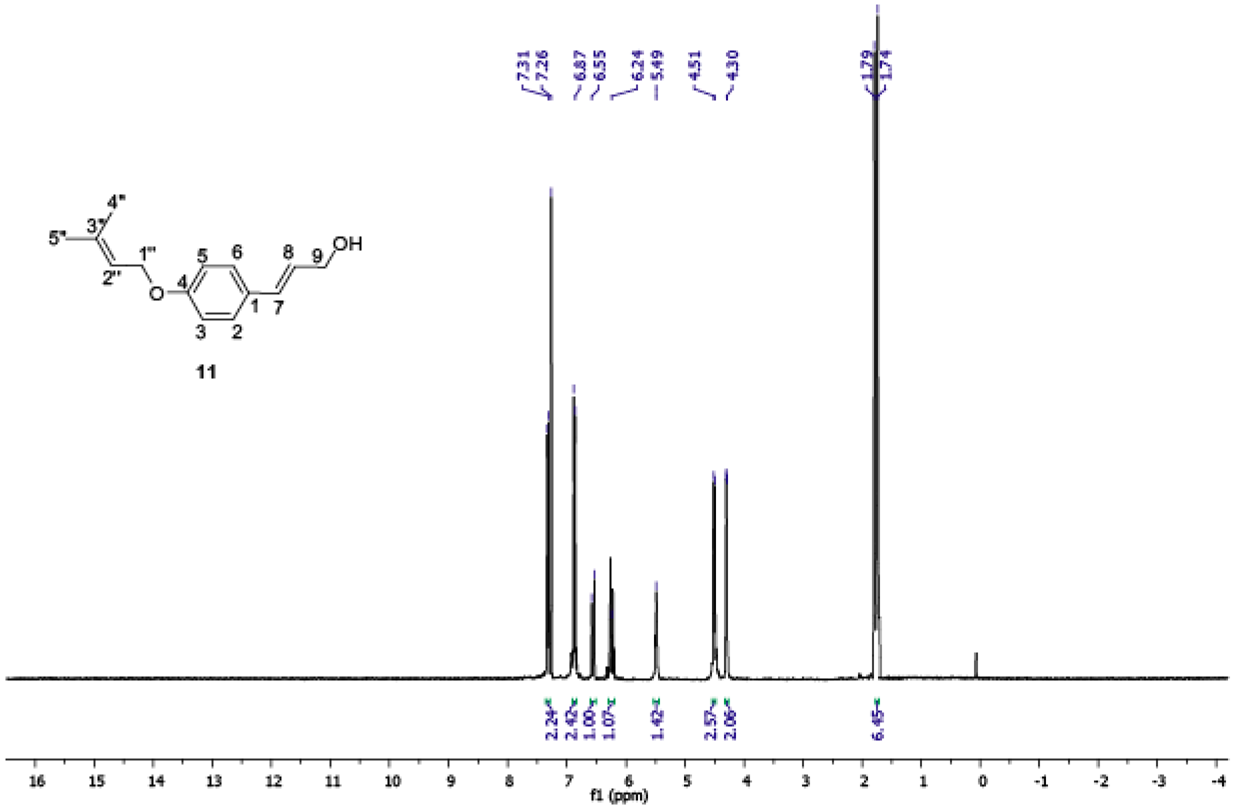

Figure S41. ^{1}H NMR spectrum of compound **11** (400 MHz, $CDCl_3$, TMS).

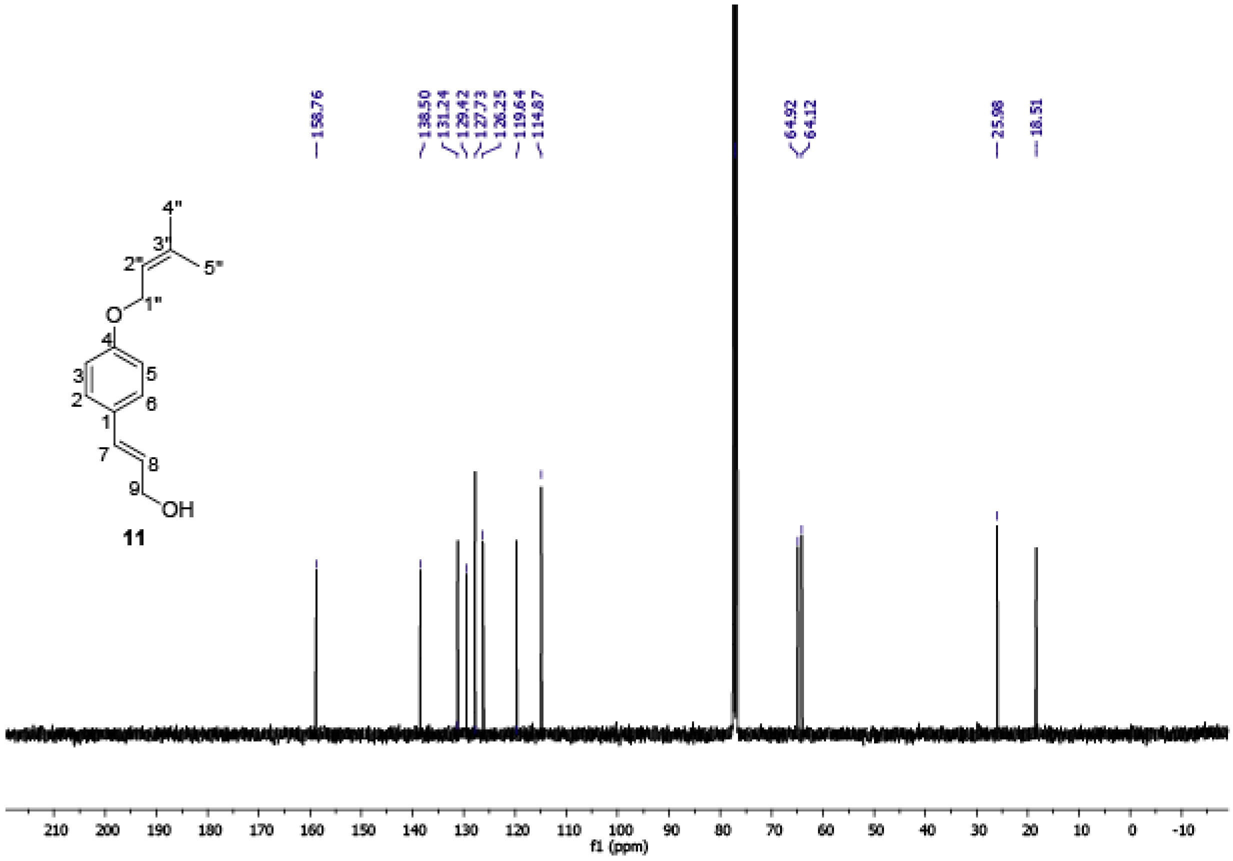

Figure S42. ^{13}C NMR spectrum of compound **11** (400 MHz, $CDCl_3$, TMS).

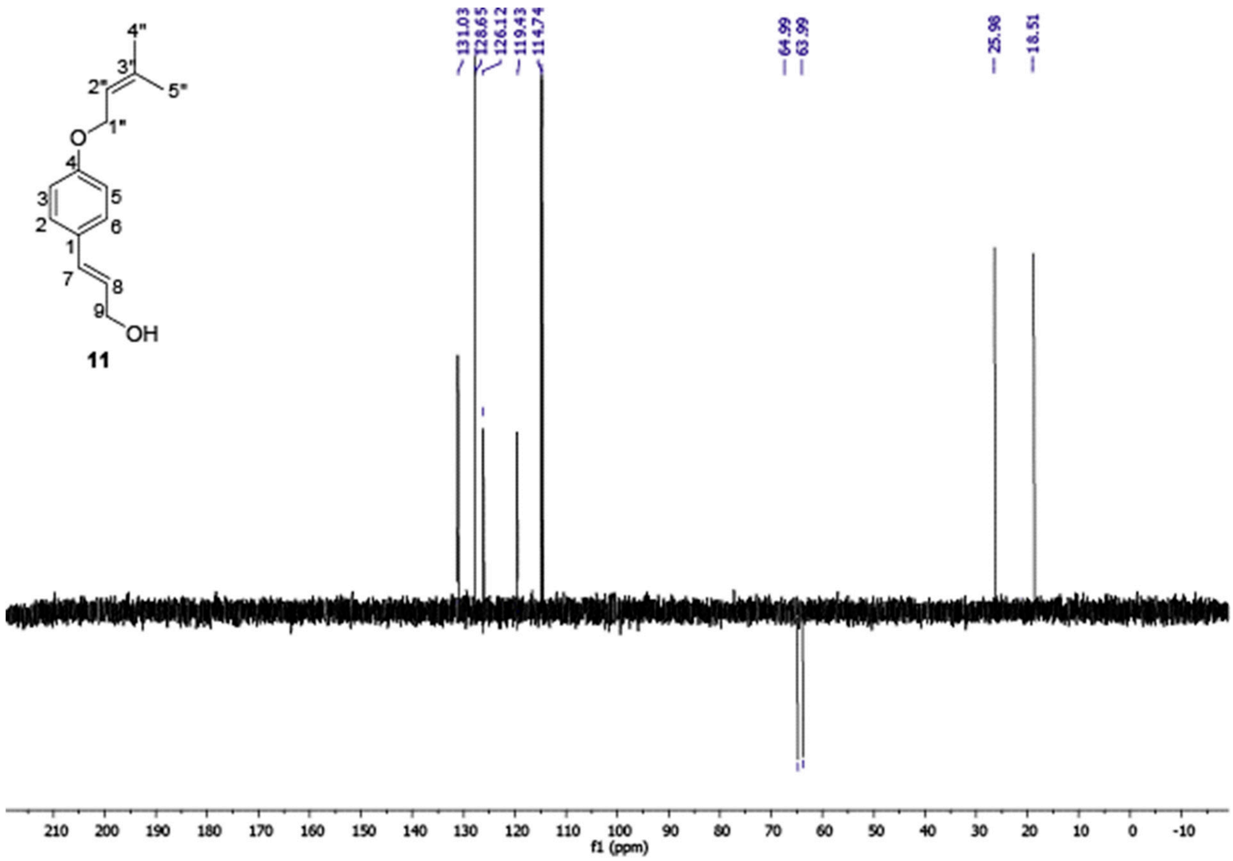

Figure S43. DEPT 135 spectrum of compound **11** (100 MHz, $CDCl_3$, TMS).

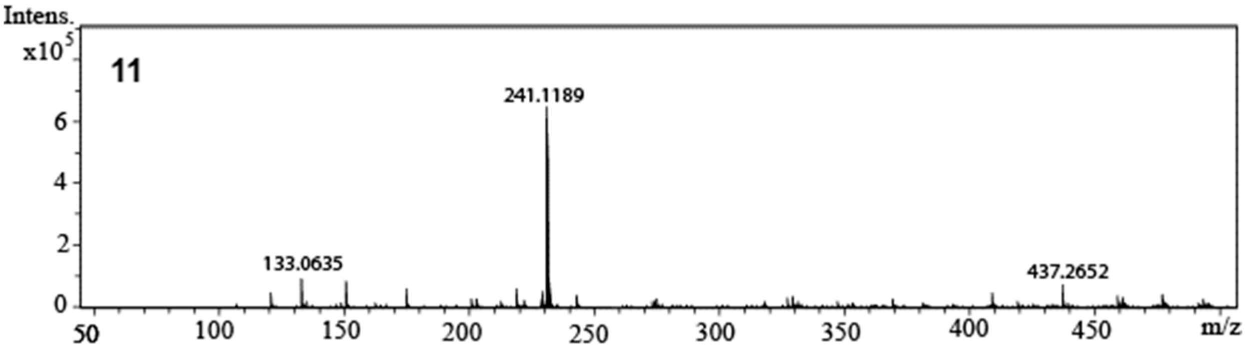

Figure S44. ESI(+)-MS spectrum of compound **11** (Q-TOF).

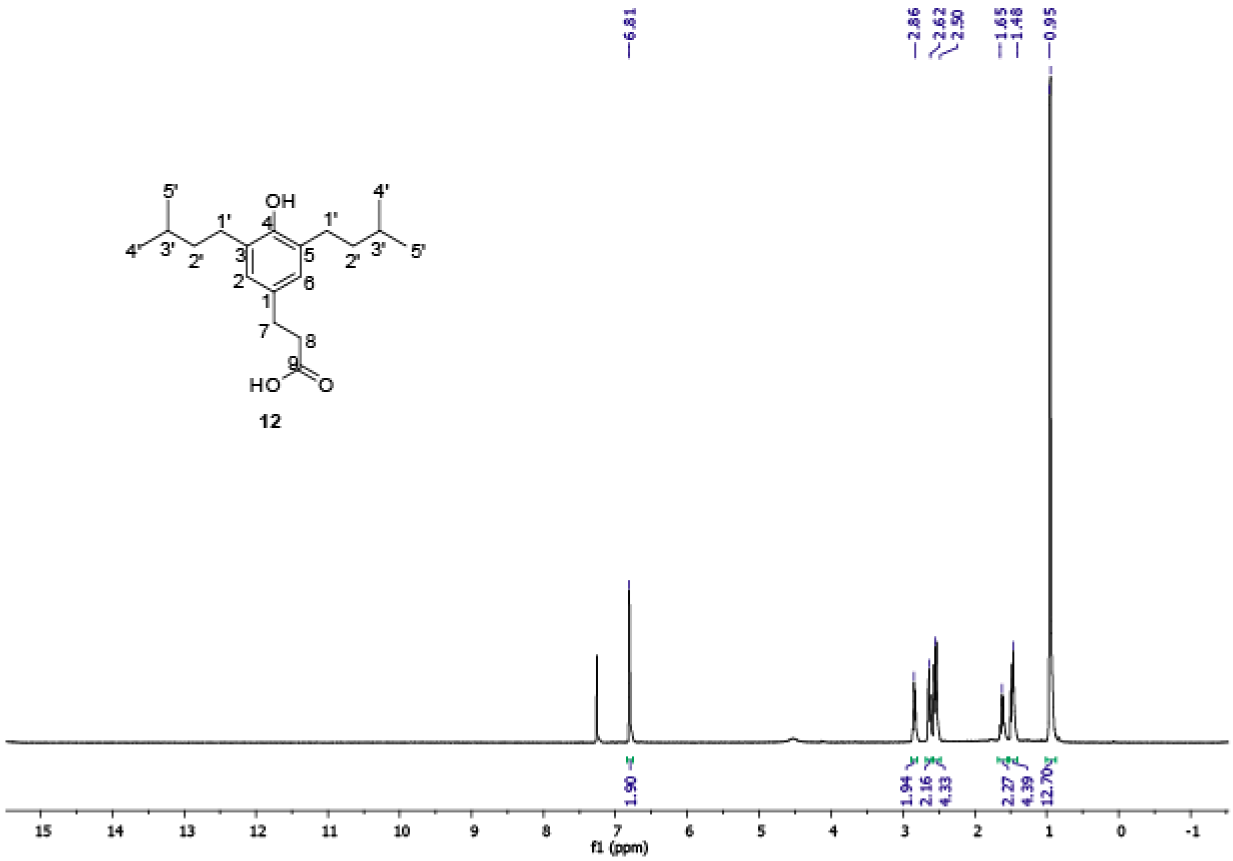

Figure S45. ^{1}H NMR spectrum of compound **12** (400 MHz, $CDCl_3$, TMS).

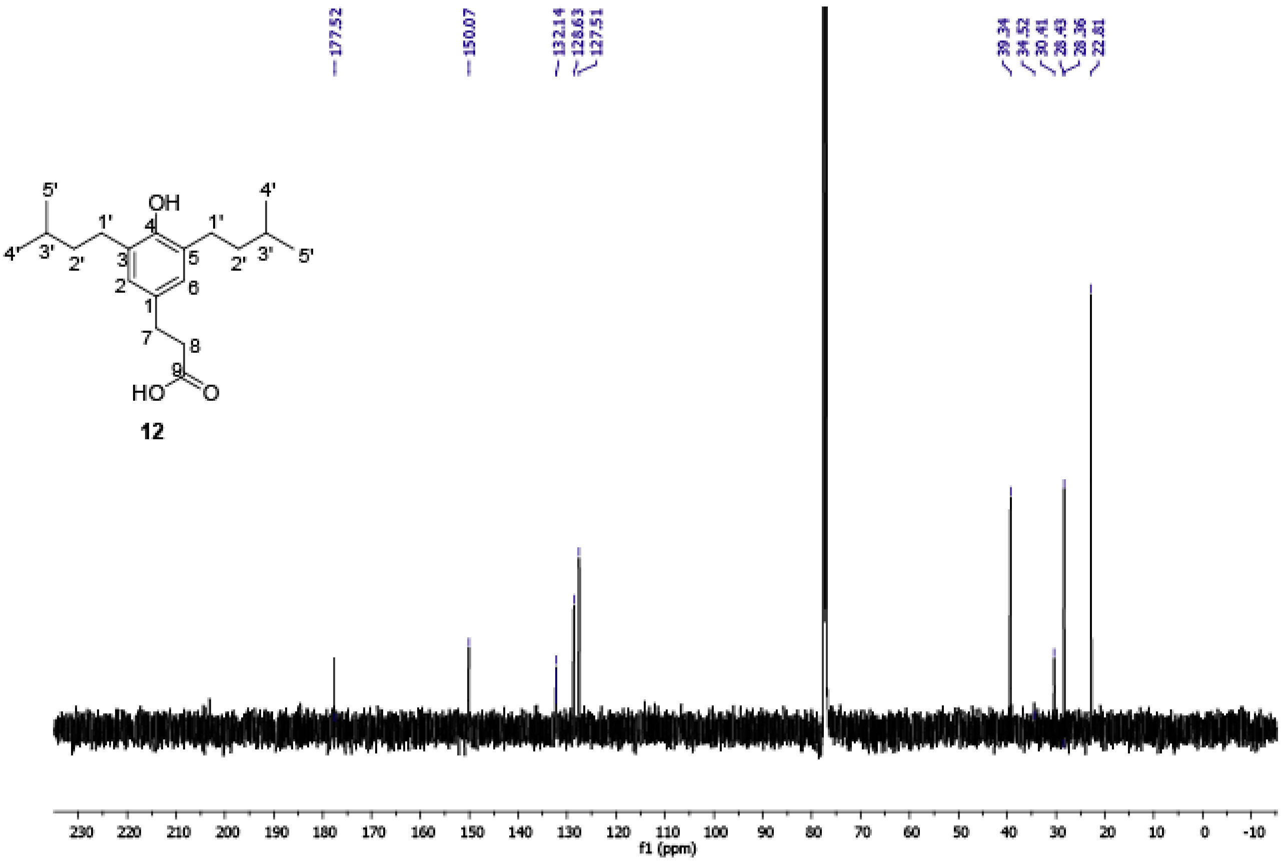

‘**Figure S46.** ^{13}C NMR spectrum of compound **12** (400 MHz, $CDCl_3$, TMS).

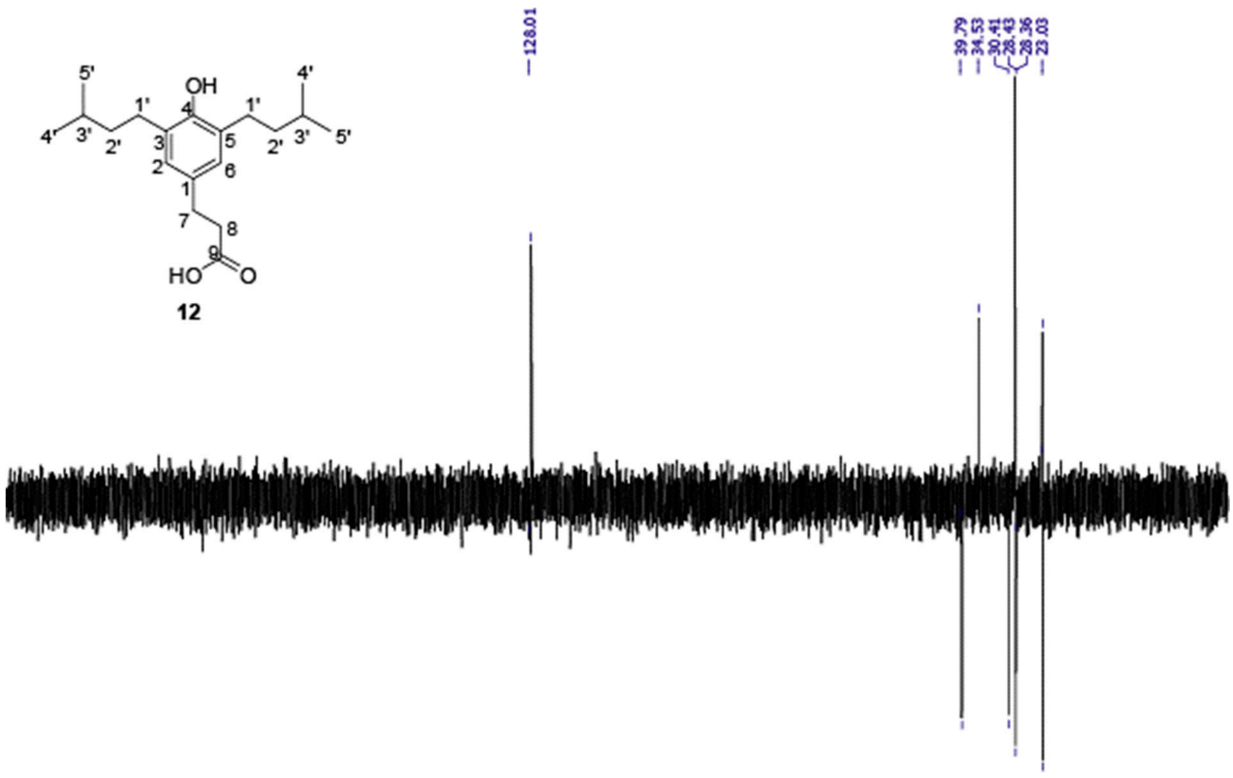

Figure S47. DEPT 135 spectrum of compound **12** (100 MHz, $CDCl_3$, TMS)

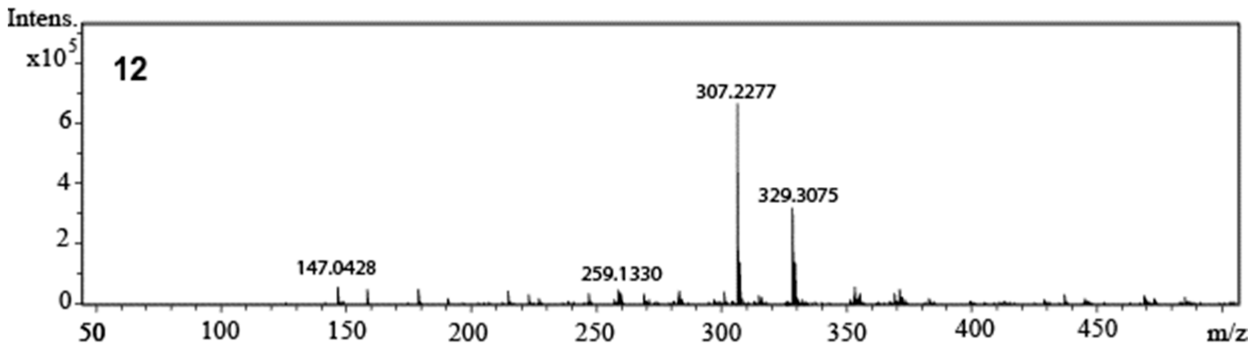

Figure S48. ESI(+)-MS spectrum of compound **12** (Q-TOF).

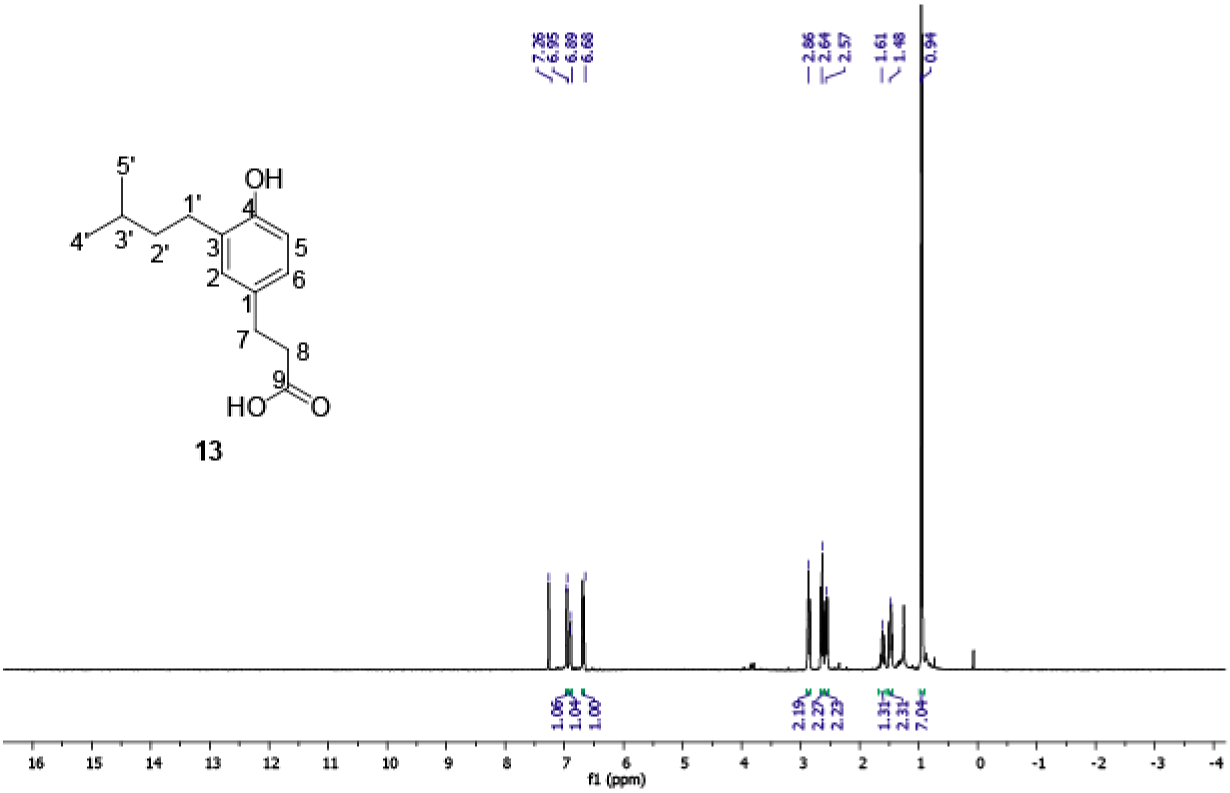

Figure S49. ^{1}H NMR spectrum of compound **13** (400 MHz, $CDCl_3$, TMS).

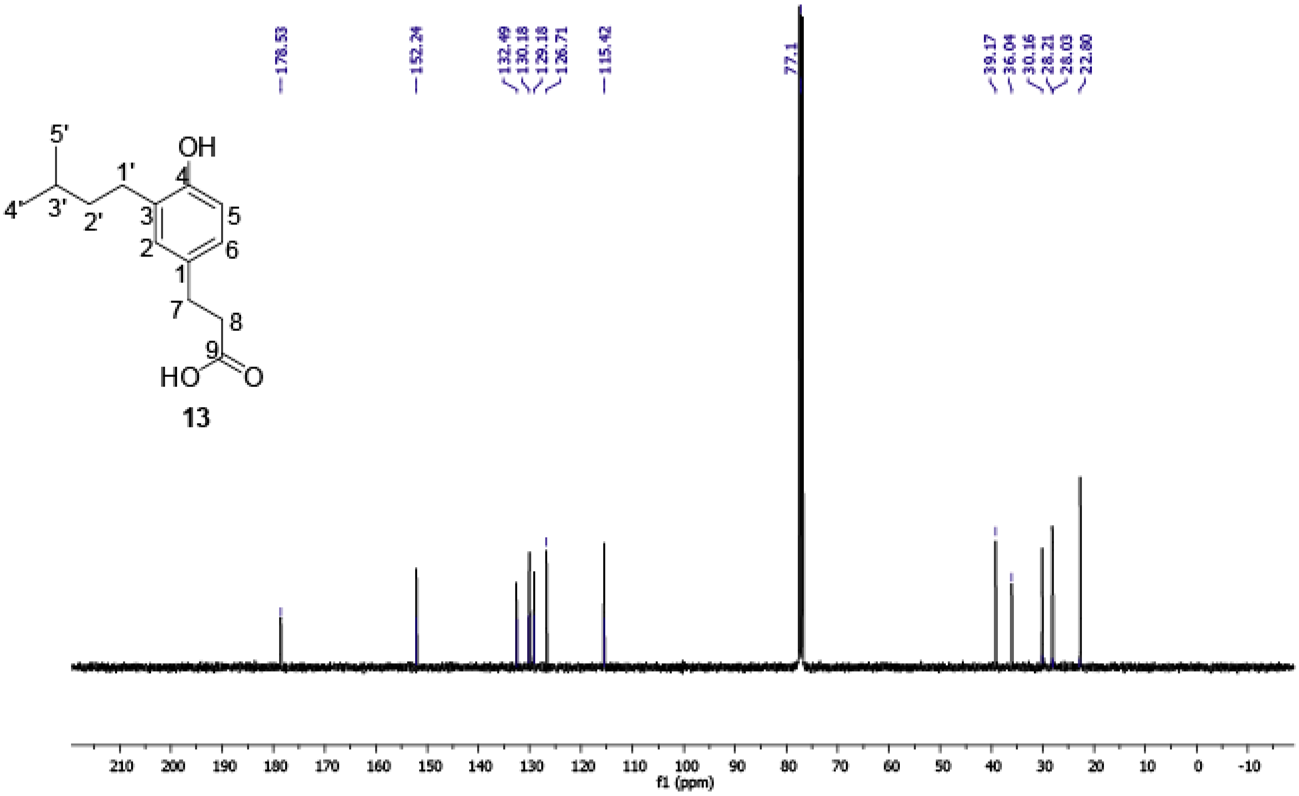

Figure S50. ^{13}C NMR spectrum of compound **13** (400 MHz, $CDCl_3$, TMS).

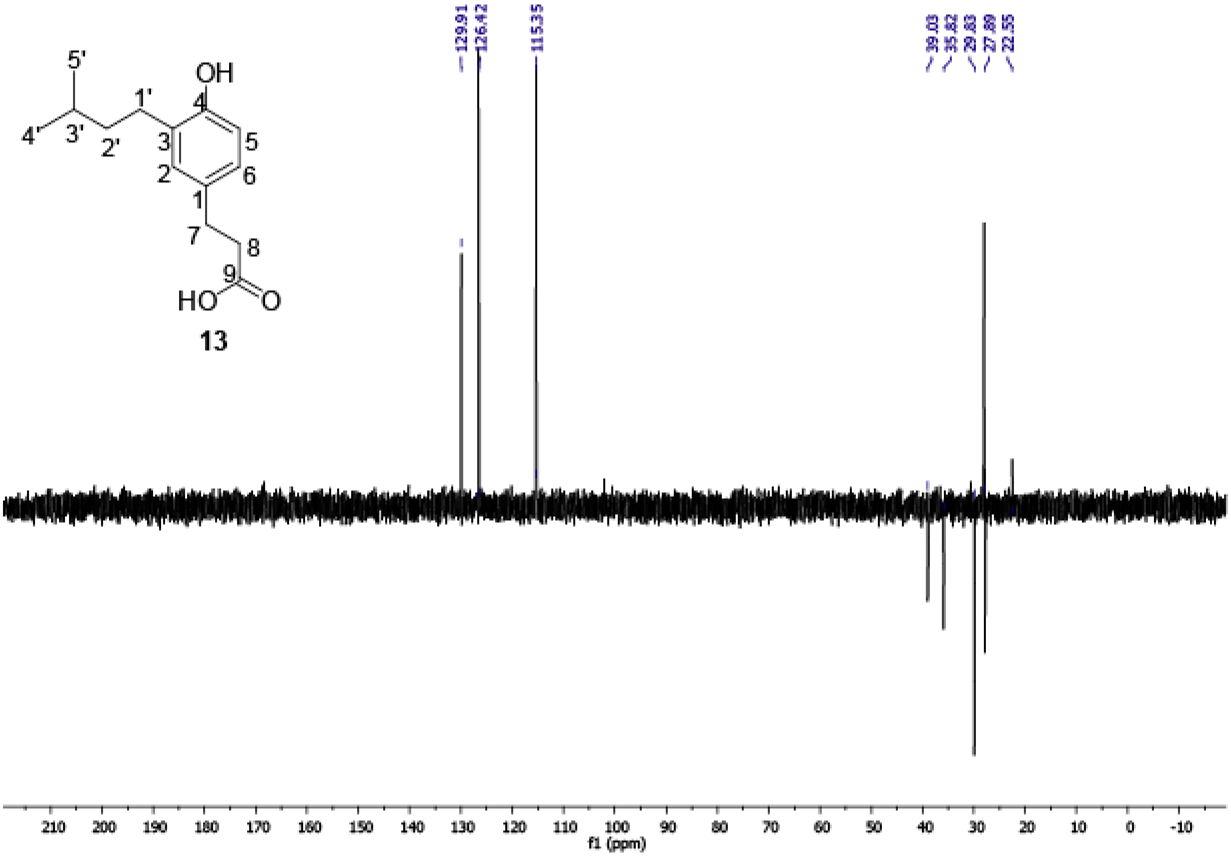

Figure S51. DEPT 135 spectrum of compound **13** (100 MHz, $CDCl_3$, TMS)

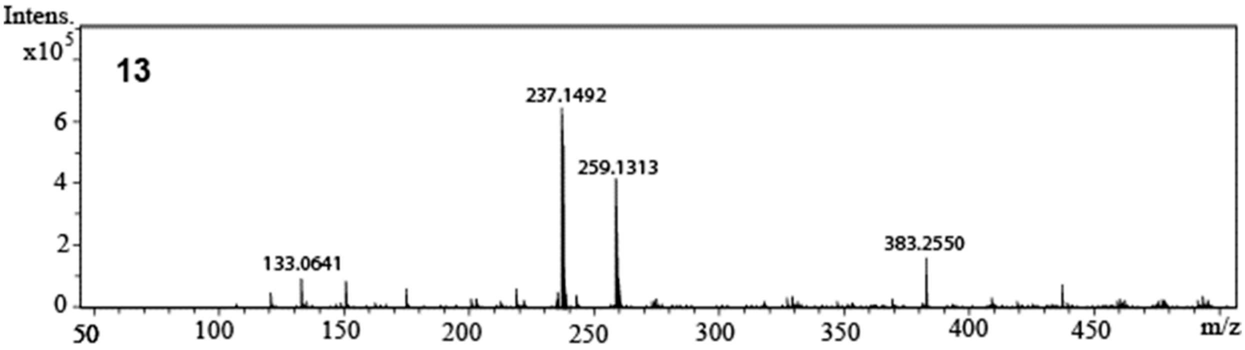

Figure S52. ESI(+)-MS spectrum of compound **13** (Q-TOF).

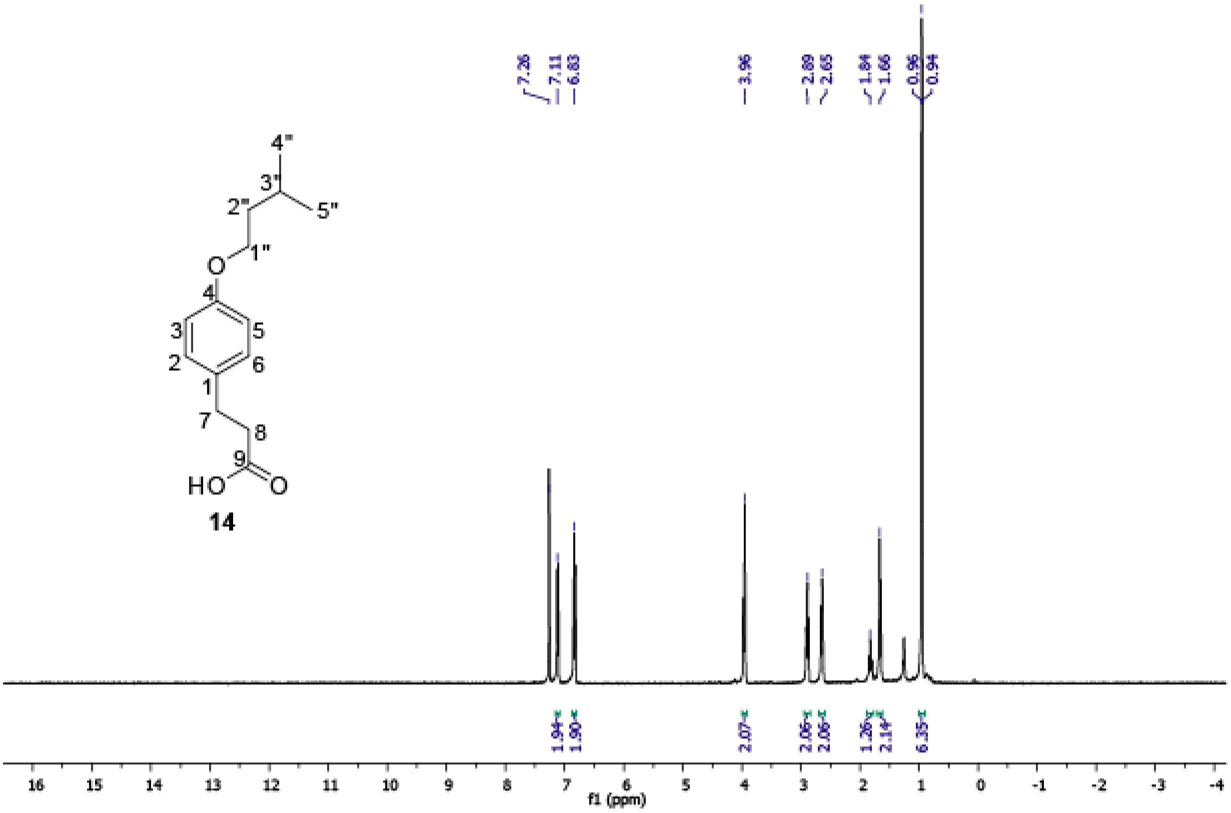

Figure S53. ^{1}H NMR spectrum of compound **14** (400 MHz, $CDCl_3$, TMS).

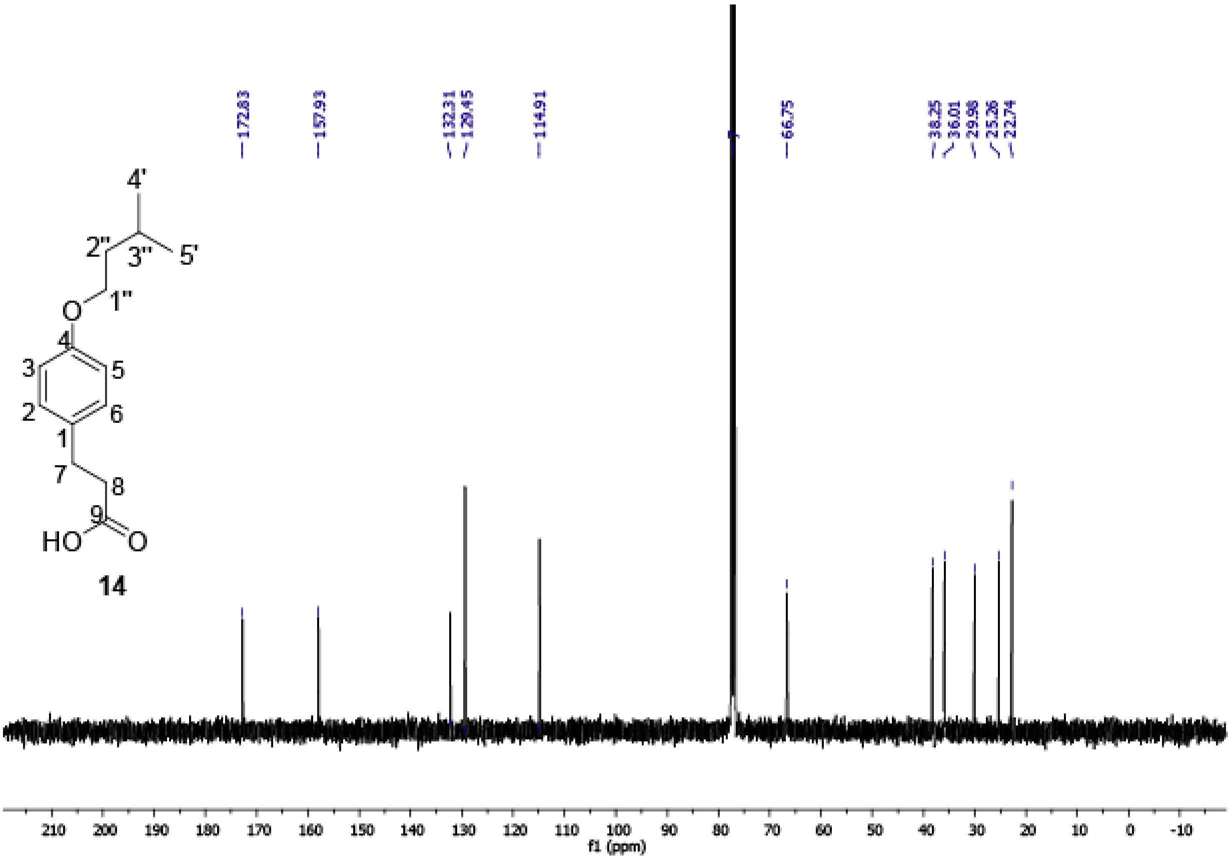

Figure S54. ^{13}C NMR spectrum of compound **14** (400 MHz, $CDCl_3$, TMS).

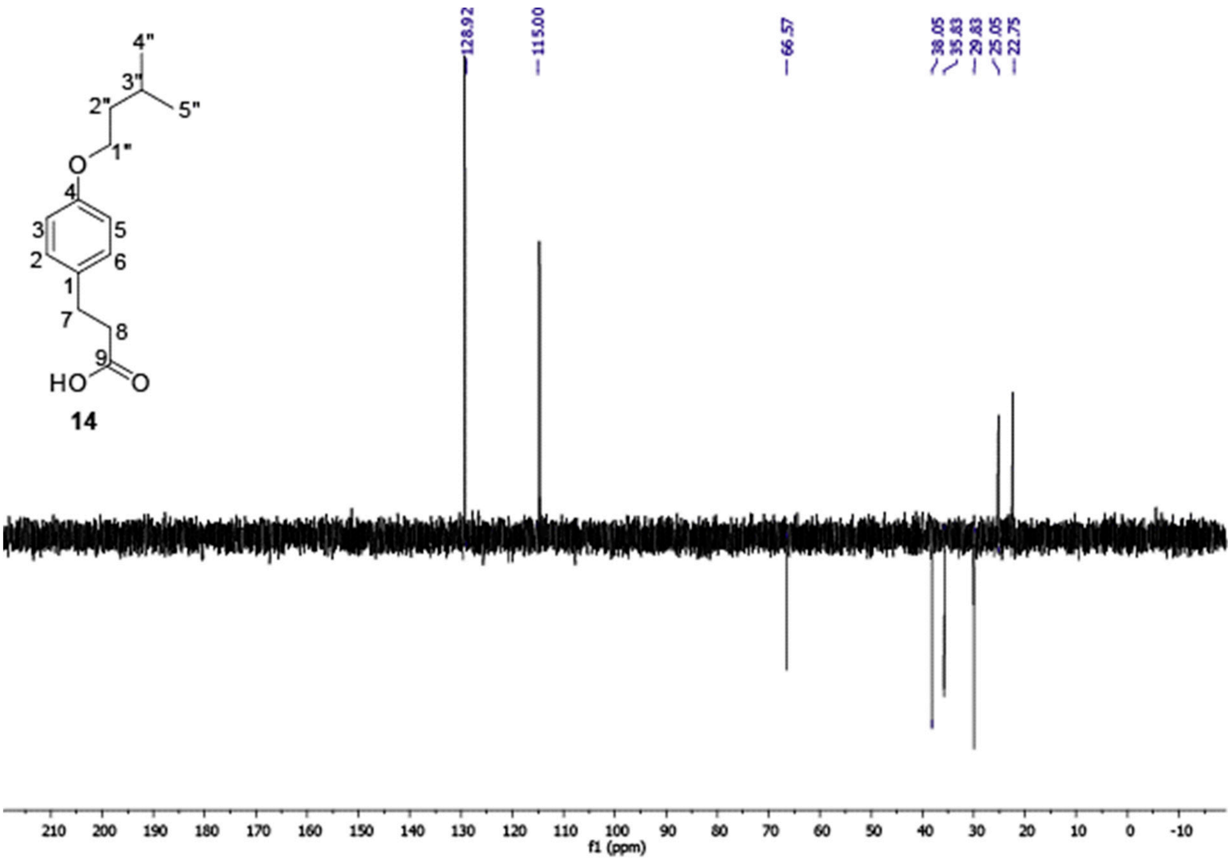

Figure S55. DEPT 135 spectrum of compound **14** (100 MHz, $CDCl_3$, TMS)

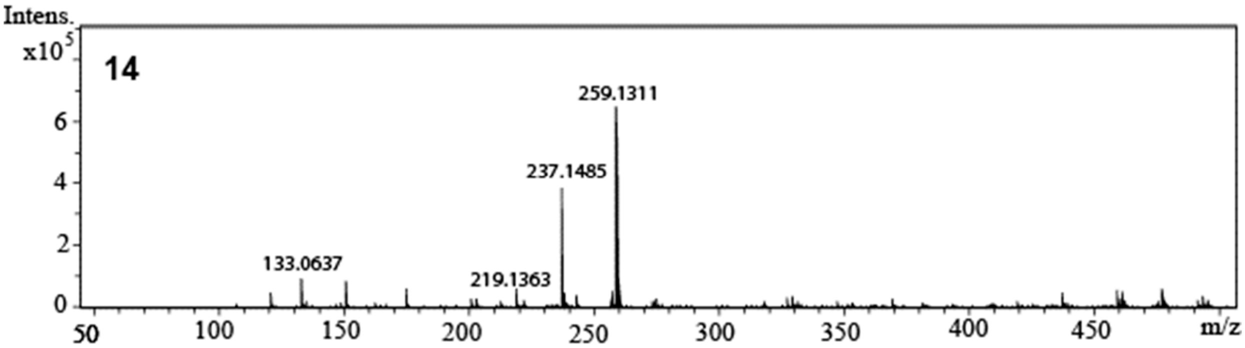

Figure S56. ESI(+)-MS spectrum of compound **143** (Q-TOF).
